# 4-(*N*-Alkyl- and -Acyl-amino)-1,2,4-triazole-3-thione Analogs as Metallo-β-Lactamase Inhibitors: Impact of 4-Linker on Potency and Spectrum of Inhibition

**DOI:** 10.3390/biom10081094

**Published:** 2020-07-23

**Authors:** Laurent Gavara, Federica Verdirosa, Alice Legru, Paola Sandra Mercuri, Lionel Nauton, Laurent Sevaille, Georges Feller, Dorothée Berthomieu, Filomena Sannio, Francesca Marcoccia, Silvia Tanfoni, Filomena De Luca, Nohad Gresh, Moreno Galleni, Jean-Denis Docquier, Jean-François Hernandez

**Affiliations:** 1 Institut des Biomolécules Max Mousseron, UMR5247 CNRS, Université de Montpellier, ENSCM, Faculté de Pharmacie, 34093 Montpellier, France; alice.legru@umontpellier.fr (A.L.); laurent.sevaille@curie.fr (L.S.); 2 Dipartimento di Biotecnologie Mediche, Università di Siena, I-53100 Siena, Italy; federica.verdirosa@student.unisi.it (F.V.); filomena.sannio@unisi.it (F.S.); francesca.marcoccia@unisi.it (F.M.); tanfoni.silvia@gmail.com (S.T.); deluca19@unisi.it (F.D.L.); 3 Laboratoire des Macromolécules Biologiques, Centre d’Ingénierie des Protéines-InBioS, Université de Liège, Institute of Chemistry B6 a, Sart-Tilman, 4000 Liège, Belgium; pmercuri@uliege.be (P.S.M.); mgalleni@uliege.be (M.G.); 4 Institut de Chimie de Clermont-Ferrand, Université Clermont-Auvergne, CNRS, SIGMA Clermont, 63000 Clermont-Ferrand, France; lionel.nauton@uca.fr; 5 Laboratoire de Biochimie, Centre d’Ingénierie des Protéines-InBioS, Université de Liège, B6, Sart-Tilman, 4000 Liège, Belgium; gfeller@uliege.be; 6 Institut Charles Gerhardt, UMR5253, CNRS, Université de Montpellier, ENSCM, Cedex 5, 34296 Montpellier, France; dorothee.berthomieu@enscm.fr; 7 Laboratoire de Chimie Théorique, UMR7616, Sorbonne Université, CNRS, 75252 Paris, France; gresh@lct.jussieu.fr

**Keywords:** metallo-β-lactamase, 1,2,4-triazole-3-thione, bacterial resistance, β-lactam antibiotics, enzyme inhibitors

## Abstract

To fight the increasingly worrying bacterial resistance to antibiotics, the discovery and development of new therapeutics is urgently needed. Here, we report on a new series of 1,2,4-triazole-3-thione compounds as inhibitors of metallo-β-lactamases (MBLs), which represent major resistance determinants to β-lactams, and especially carbapenems, in Gram-negative bacteria. These molecules are stable analogs of 4-amino-1,2,4-triazole-derived Schiff bases, where the hydrazone-like bond has been reduced (hydrazine series) or the 4-amino group has been acylated (hydrazide series); the synthesis and physicochemical properties thereof are described. The inhibitory potency was determined on the most clinically relevant acquired MBLs (IMP-, VIM-, and NDM-types subclass B1 MBLs). When compared with the previously reported hydrazone series, hydrazine but not hydrazide analogs showed similarly potent inhibitory activity on VIM-type enzymes, especially VIM-2 and VIM-4, with *K*_i_ values in the micromolar to submicromolar range. One of these showed broad-spectrum inhibition as it also significantly inhibited VIM-1 and NDM-1. Restoration of β-lactam activity in microbiological assays was observed for one selected compound. Finally, the binding to the VIM-2 active site was evaluated by isothermal titration calorimetry and a modeling study explored the effect of the linker structure on the mode of binding with this MBL.

## 1. Introduction

In the early 1900s, bacterial infections represented one of the most significant causes of death in the world. This plague has been circumvented by the development of several classes of antibiotics, including the β-lactams, which show optimal efficacy and selectivity. This therapeutic class is characterized by a four-membered β-lactam ring which is crucial for its antibacterial activity. Indeed, this ring, also named azetidinone, is involved in the inhibition of the bacterial cell wall synthesis by mimicking the D-Ala-D-Ala moiety of the natural substrate of the Penicillin-Binding Proteins (PBPs) in the reticulation process [1]. β-lactam antibiotics include four sub-families: penicillins, cephalosporins, monobactams, and carbapenems. The latter were specifically developed for the treatment of infections caused by multidrug-resistant Gram-negative opportunistic pathogens, and are exclusively used in the hospital setting, commonly as a last resort therapeutic option.

Due to the natural selective pressure exerted by exogenous agents, bacteria developed a plethora of resistance mechanisms to allow their survival in selective conditions and, consequently, significantly compromise the therapeutic efficacy of antibiotics [2,3]. Multidrug-resistant bacteria, now widespread in the clinical setting and potentially causing life-threatening infections, commonly exhibit a combination of resistance mechanisms, acting synergistically to both increase the level of resistance to individual drugs and broaden the resistance spectrum [4,5]. In response to this deadly threat, and the fast evolution of MDR strains towards pan-drug resistance phenotypes, a global awareness recently emerged and several governmental and private action plans were established. The WHO identified a list of priority pathogens against which new antibacterials would be extremely desirable that includes carbapenem-resistant Enterobacteriaceae, *Pseudomonas aeruginosa*, and *Acinetobacter baumannii* [6,7,8,9].

In Gram-negative bacteria, the major mechanism of resistance to β-lactams is mediated by the production of β-lactamases (BLs), enzymes able to hydrolyze the amide bond of the azetidinone (β-lactam) ring [10,11]. They are classified into four molecular classes (A–D) according to their structural properties and catalytic mechanism. Serine-β-lactamases (SBLs), belonging to classes A, C, and D, are related to serine hydrolase enzymes and are commonly inhibited by clavulanate, avibactam, and vaborbactam [12]. Three decades ago, and shortly after the introduction of carbapenems into clinical practice, acquired metallo-β-lactamases (MBLs), belonging to Ambler’s class B, emerged in Gram-negative opportunistic pathogens [13,14]. They are characterized by the presence of one or two zinc atoms in the active site. The ions act as Lewis acids that increase the electrophilicity of the azetidinone ring while promoting the depolarization of a water molecule to afford the hydroxide nucleophile [13]. The diffusion and prevalence of MBL-producing clinical isolates has significantly increased in the last twenty years, and they are now recognized as one of the most worrying threats to the success of antibacterial chemotherapy. Indeed, MBLs are able to inactivate a wide range of β-lactam substrates, including carbapenems. MBLs can be subdivided into three subclasses according to the nature and position of metal-coordinating residues and the zinc requirement. Subclass B1 enzymes account for the most prevalent and clinically-relevant MBLs [14,15]. Among them, VIM-type variants, which are most widely spread in Europe and in North America, are able to hydrolyze almost all marketed β-lactams [16]. Because of a different catalytic mechanism compared to SBLs, the marketed SBL inhibitors are ineffective against MBLs. There is currently no approved MBL inhibitor, despite the fact that they would represent a significant and desirable addition for the treatment of difficult infections in the clinical setting. The main difficulties are the identification of a broad-spectrum inhibitor and, because most inhibitors contain a zinc-coordinating group, the risk for insufficient selectivity toward MBLs with off-target effects on important human metalloenzymes. Among numerous reported series of MBL inhibitors [17], the largest families of compounds possess a thiol group (see, e.g., [18,19]) or at least two carboxylate groups, such as succinate analogs [20], dipicolinates [21], or aspergillomarasmine [22]. Although these groups could afford potent inhibitors because of strong Zn coordination, the risk for insufficient selectivity is high [23]. Despite intense research efforts, only one compound has reached clinical development (i.e., VNRX-5133), a pan-spectrum inhibitor active on both SBLs and MBLs with a cyclic boronate structure [24,25,26,27]. A few other lead compounds that could efficiently restore bacterial sensitivity to carbapenems, including in in vivo infection models, were also reported [28,29,30].

**Figure 1:**
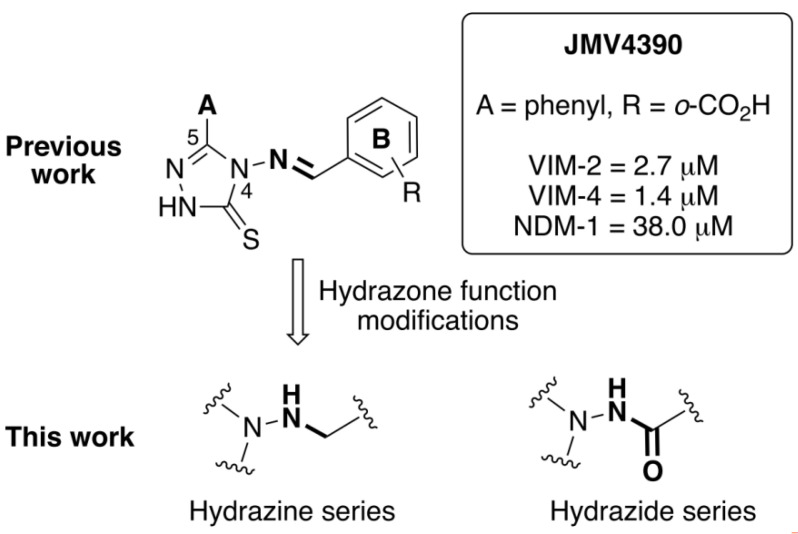
Previous work on hydrazone-like compounds and design of new stable series (*K*_i_ values are given for Schiff base JMV4390 (see the unpublished work in [37])).

In this context, the development of selective and potent inhibitors of MBLs is clearly a medicinal challenge that is crucial to maintain the efficacy of our last line of defense against bacterial pathogens [31,32]. Based on previous work by L. Olsen et al. [33] and L. Nauton et al. [34], we have recently described new series of MBL inhibitors composed of an original dizinc-coordinating scaffold, the 1,2,4-triazole-3-thione heterocycle, which was shown to simultaneously coordinate the two active site zincs through a nitrogen of the triazole ring and the sulfur atom (see the works in [34,35,36] and the unpublished work in [37]). In particular, modulation of the substituents of Schiff base analogs at positions 5 and 4 led to the discovery of potent MBL inhibitors such as JMV 4390 (Figure 1). In this series, a carboxylic group in the ortho position of the phenyl ring B was found to be associated with broad-spectrum inhibition of MBLs (i.e., targeting at least the three most prevalent MBLs, IMP-1, VIM-2, and NDM-1). However, such compounds showed a limited antibacterial activity in combination with β-lactams, likely due to a limited penetration into the bacterial periplasm, but also potentially by the hydrolytic sensitivity of the hydrazone-like bond leading to subsequent formation of the less potent 4-amino-1,2,4-triazole-3-thione fragment [35]. The hydrolysis can be spontaneous in aqueous media or may be promoted by the catalytic action mediated by enzymes [34]. To solve this potential instability issue, we decided to replace the hydrazone function by more stable linkers. Two types of modifications were investigated: (i) the hydrazine-like series resulting from the reduction of the corresponding Schiff base derivative and (ii) the hydrazide-like series. Although the latter are still potentially susceptible to hydrolysis, the hydrazide-like function is more chemically stable (Figure 1). In the first series, the carbon adjacent to the extra-cyclic nitrogen is sp^3^, whereas it is sp^2^ in the second series as in the hydrazone-like function. The compounds were evaluated for their inhibitory activity on relevant MBLs, their ability to restore susceptibility to antibiotics in whole cell assays, as well as their mode of binding.

## 2. Materials and Methods

### 2.1. Chemistry

#### 2.1.1. Typical Procedure of Hydrazone Reduction (Synthesis of Hydrazines **1**–**12**)

To a solution of a hydrazone compound [37] (0.5 mmol, 1 equiv.) in methanol (5 mL) was added sodium borohydride (189 mg, 5 mmol, 10 equiv.) under inert atmosphere. The mixture was heated at reflux for 1 h and evaporated. The residue was finally purified by chromatography on silica gel to yield the corresponding hydrazine compound.

#### 2.1.2. Typical Procedure for Succinic Anhydride Condensation (Synthesis of Succinimides **13**–**16**)

A mixture of a 4-amino-1,2,4-triazole-3-thione compound [35] (1 equiv., 1 mmol) and cyclic anhydride (2 equiv., 2 mmol) in pyridine (1 mL) was refluxed for 1 h. The solution was then diluted in EtOAc (20 mL) and washed with water (4 × 30 mL). The organic layer was dried over MgSO_4_ and evaporated to give a crude powder. The resulting succinimide was purified by recrystallization from a mixture of EtOAc-Hexane.

#### 2.1.3. Typical Procedure of Succinimide Ring-Opening Reaction (Synthesis of Hydrazides **17**–**20**)

A succinimide (1 equiv., 0.5 mmol) was added to a solution of KOH (330 mg, 10 equiv., 5 mmol) in a mixture of EtOH-H_2_O (2-1/6 mL) and the reaction was stirred at room temperature for 2 h. The mixture was acidified by an aqueous solution of KHSO_4_ 1 N (10 mL) and extracted by EtOAc (3 × 20 mL). The combined organic layers were dried over MgSO_4_ and evaporated to give a crude powder. Recrystallization from EtOAc led to the pure hydrazide compound.

### 2.2. Metallo-β-Lactamase Inhibition Assays

#### 2.2.1. VIM-Type Enzymes: NDM-1 and IMP-1

The inhibition potency of the compounds has been assessed with a reporter substrate method and specifically by measuring the rate of hydrolysis of the reporter substrate (such as 150 μM imipenem, 150 μM meropenem, 120 μM cefotaxime, or 100 μM nitrocefin) at 30 °C in 50 mM HEPES (Promega, Madison, WI, USA) buffer (pH 7.5) in the absence and presence of several concentrations of the inhibitor (final concentration: 0.5–1000 µM). Compounds were prepared in DMSO at a final concentration of 50 mM prior to dilution in the assay buffer. The reaction rate was measured as the variation of absorbance observed upon substrate hydrolysis in a UV-Vis spectrophotometer or microplate reader at a wavelength of 300 nm (imipenem, meropenem), 260 nm (cefotaxime), or 482 nm (nitrocefin) and in the presence of a purified MBL enzyme (such as VIM-1, VIM-2, VIM-4, NDM-1, and IMP-1, at a final concentration ranging from 1 to 70 nM).

These enzymes were overproduced in derivatives of *E. coli* BL21(DE3) carrying a pET-9 a (IMP-1, VIM-1, VIM-2, and VIM-4) or pET-15 b (NDM-1) vector into which the metallo-β-lactamase-encoding open reading frame was cloned. Bacterial strains were grown in the auto-inducing medium, ZYP-5052, supplemented with 50 μg/mL kanamycin for 24 h at 37 °C [38]. IMP-1, VIM-1, VIM-2, and VIM-4 were purified by ion-exchange and gel filtration chromatography using an AKTA Purifier platform (GE Healthcare, Uppsala, Sweden) as previously described [39]. NDM-1 (residues 37-270) was produced as an amino-terminal 6 xHis-tag fusion protein and was purified using affinity chromatography. After growth for 26 h at 25 °C, bacterial cells were harvested by centrifugation (10,000× *g*, 30 min, 4 °C), resuspended in 10 mM HEPES buffer (pH 7.5) containing 0.15 M NaCl (buffer A) in one-fifth of the original culture volume, and lysed by sonication. Cellular debris was removed by centrifugation (12,000× *g*, 40 min, 4 °C). The clarified sample was loaded on a HisTrap HP column (bed volume, 5 mL; GE Healthcare), followed by a wash step (25 mL) with buffer A containing 20 mM imidazole. Proteins were eluted using buffer A containing 350 mM imidazole. The resulting protein sample was desalted (HiPrep 26/10 Desalting column, GE Healthcare) using 20 mM triethanolamine (pH 7.2) supplemented with 50 μM ZnSO_4_ and concentrated using an Amicon Ultra-15 (MW cut-off, 10 kDa; Millipore, Burlington, MA, USA). The concentrated sample was loaded on a Superdex 75 prep grade column (XK16/100 column; bed volume, 140 mL; GE Healthcare) and the proteins eluted with buffer A containing 50 μM ZnSO_4_. The active fractions were pooled and stored at −20 °C. The purity of the protein preparations was estimated >95% by SDS-PAGE analysis. The authenticity of the enzyme preparations was confirmed by electron spray ionization mass spectrometry, as previously described [40].

The inhibition constants (*K*_i_) were determined on the basis of a model of competitive inhibition by analyzing the dependence of the ratio *v*_0_/*v*_i_ (*v*_0_, hydrolysis velocity in the absence of inhibitor; *v*_i_, hydrolysis velocity in the presence of inhibitor) as a function of [I] as already described [41]. The slope of the plot of *v*_0_/*v*_i_ vs. [I], which corresponds to *K*_m_^S^/(*K*_m_^S^ + [S])*K_i_* (where *K*_m_^S^ is the *K*_m_ value of the reporter substrate and [S] its concentration in the reaction mixture) and allowed the calculation of the *K_i_* value. Alternatively, a Dixon plot analysis was carried out by measuring the initial hydrolysis rates in the presence of variable concentrations of inhibitor and substrate. This allowed *K_i_* values to be determined and supported the hypothesis that the various compounds behaved as competitive inhibitors of the various tested enzymes. The assays were performed in triplicate.

#### 2.2.2. CphA and L1

Compounds were prepared as 10 mM solutions in DMSO before dilution with appropriate buffers. Buffers were 25 mM Hepes pH 7.5 for CphA or 25 mM Hepes pH 7.5, 50 µM ZnCl_2_ for L1. The rates in the absence of inhibitor were not modified by addition of 1% DMSO. CphA and L1 enzymes, purified as previously described [34,42,43], were used at concentrations between 0.2 and 0.8 nM, respectively. The enzyme and the inhibitor (100 µM) were pre-incubated for 30 min in a volume of 495 µL at room temperature. The hydrolysis of a solution of 100 µM imipenem for CphA was monitored by following the absorbance variation at 300 nm using a Specord 50 PLUS spectrophotometer (Analytik Jena, Jena, Germany). For L1, the reporter substrate was nitrocefin (100 µM). The enzyme activity was measured by following the absorbance variation at 482 nm. The substrate hydrolysis was done at 30 °C. The activity was tested by measuring the initial rates in three samples without inhibitor that allowed to determine the percentage of residual β-lactamase activity in the presence of inhibitors. If the residual activity in the presence of 100 µM inhibitor was <30%, the initial rate conditions were used to study the inhibition in the presence of increasing concentrations of compounds (from 1 to 100 µM) and to determine the competitive inhibition constant *K*_i_. The Hanes linearization of the Henri–Michaelis equation was used, and *K*_i_ was calculated on the basis of the following equation, v = (Vmax.S)/[S + Km(1 + I/Ki)]. The assays were performed in triplicates.

### 2.3. Microbiological Assays

The potential activity of selected compounds was assessed by the agar disk-diffusion method, according to the CLSI (Clinical Laboratory Standard Institute) recommendations, using Mueller–Hinton medium [44]. The inhibitor (dissolved in DMSO) was added (maximum volume of 15 μL) to a commercially available disk containing a defined amount of a β-lactam antibiotic (30 μg of cefoxitin (Oxoid, Milan, Italy)). After incubation (18 h at 35 °C), the diameter of the growth inhibition zone was measured and compared to that obtained in the absence of inhibitor. DMSO and EDTA (220 μg) were used as negative and positive controls, respectively. Experiments were performed in triplicate.

These tests were performed on an isogenic laboratory strain obtained after transforming *Escherichia coli* LZ2310 strain with a derivative of the high copy number plasmid pLB-II carrying the cloned *bla*_VIM-2_ gene [45] harboring a cloned gene encoding the functional metallo-β-lactamase. Strain LZ2310 is a triple knockout mutant derived from K12 in which the genes encoding for components of the three major efflux pump systems were inactivated (genotype, *∆norE ∆mdfA N43 acrA1*) [46] and was used to generate an hyperpermeable background for MBL production and investigate the potential contribution of such mechanisms to inhibitor efflux.

### 2.4. Isothermal Titration Calorimetry Analysis of Binding to VIM-2

ITC titrations were performed on a MicroCal ITC200 (GE-Malvern) equipped with a 200 µL Hastelloy sample cell and an automated 40 µL glass syringe rotating at 1000 rpm. VIM-2 in 10 mM Hepes-NaOH, 0.15 M NaCl, 50 µM ZnSO_4_, pH 7.5 was diluted to the desired concentration with the same buffer and was brought to a DMSO concentration identical to that of the injected compound. The tested compound was solubilized in DMSO at 20 mM concentration and was diluted to 200 µM with the enzyme buffer, resulting in a final DMSO concentration of 1%.

In a standard experiment, VIM-2 (19 µM) was titrated by one initiating injection (0.5 µL) followed by 19 injections (2 µL) of compound (200 µM) at an interval of 150 s. Dilution heat of compound injections into buffer, at the corresponding DMSO concentration, were subtracted from raw data. The data so obtained were fitted via nonlinear least squares minimization method to determine binding stoichiometry (*n*), association constant (*K*_a_), and change in enthalpy of binding (*∆H°_b_*) using ORIGIN 7 software v.7 (OriginLab, Northampton, MA, USA). In the fitting procedure, the compound concentration was lowered in order to provide n around 1 and to take compound solubility into account under ITC conditions. The Gibbs free energy of binding, *∆G°_b_*, was calculated from *K*_a_ (*∆G°_b_* = −*RT* ln *K*_a_) and the entropic term, T*∆S°_b_*, was derived from the Gibbs–Helmholtz equation using the experimental *∆H°_b_* value (*∆G°_b_* = *∆H°_b_* − *T∆S°_b_*).

### 2.5. Modeling Study

The geometric optimization of the compound structures was done using Gaussian 16 at DFT level of theory with B3 LYP hybrid functional and 3–21 g basis set. The resulting structures were registered as pdb files. The molecules were then the object of charge calculation using the AM1-BCC model, and the files were registered as mol2 format using the Dockprep module of Chimera software (San Francisco, CA, USA). Then, docking studies were performed with AutoDock 4.2.6 [47]. Files for the docking were prepared from (i) the structure of complex VIM-2/JMV4690 (pdb code 6 YRP) [37], which was treated as follows, water molecules (with the exception of structural waters 5 and 177), small molecules (acetate, DMSO, and ethylene glycol), and the third zinc ion were removed; the protein was protonated with Dockprep module of Chimera; charges were calculated using the AM1-BCC model and the file was registered as mol2 format; (ii) compounds and protein pdbqt files were prepared with AutoDockTools (ADT) [48]. For the protein, no protein side-chains were allowed to move.

Molecular graphics and analyses were performed with UCSF Chimera, developed by the Resource of Biocomputing, Visualization and Informatics at the University of California, San Francisco, with support from NIH P41-GM103311.

## 3. Results and Discussion

### 3.1. Chemistry

**Scheme 1:**
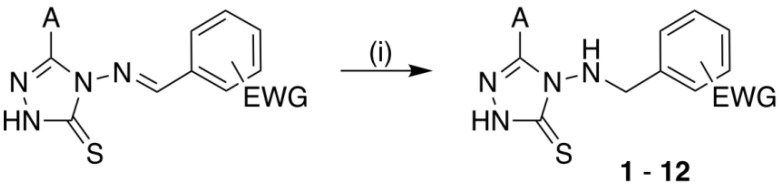
Reduction of the hydrazone-like bond of Schiff base analogs. Reagents and conditions: (i) NaBH_4_ (10 eq.), MeOH, reflux. EWG: electron withdrawing group.

In the hydrazine series, compounds **1**–**12** were obtained by the reduction of the corresponding Schiff base analogs (Scheme 1). The synthesis of the precursors has already been described [37]. Briefly, these were obtained by condensation of a 4-amino-1,2,4-triazole-3-thione compound [35] with a benzaldehyde derivative to form the hydrazone-like bond of the Schiff base analogs. The subsequent reduction was investigated under several conditions, and only the use of excess NaBH_4_ in refluxing methanol allowed obtaining the desired compounds **1**–**12**. Nevertheless, the reduction was efficient only when an electron withdrawing group (EWG) was present on the aromatic ring B in *ortho* or *para* positions. In this case, the electron delocalization allowed hydrazone reduction. In the absence of such a group, only starting material was recovered in standard conditions. When using stronger reductive agents like LiAlH_4_, BH_3_.NMe_3_ complex, Mg, or HSiEt_3_ [49], only degradation products were noticed. This may be explained by the presence of another reducible function on the molecule, the carbon-sulfur double bond. Therefore, all compounds contained a B phenyl ring substituted by a carboxylic group (at *ortho* position, **1**–**8**, **11**, and **12**, or *para* position, **10**) with the exception of compound **9** (nitro group at ortho position).

The synthesis of the second series where the hydrazone-like linker was replaced by a hydrazide-like group was first attempted by direct acylation of the 4-amino group of 4-amino-triazole-thione derivatives by acyl chlorides or carboxylic acids in the presence of various coupling agents. Unfortunately, this pathway led to a complex mixture of regioisomers because of the high chemical reactivity of the hetero atoms forming the triazole-thione moiety.

**Scheme 2:**
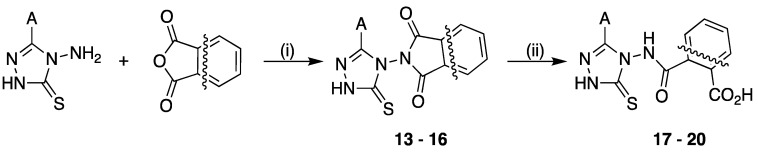
Synthetic pathway toward compounds **17**–**19** and **20** of the hydrazide-like series starting from phthalic anhydride or succinic anhydride, respectively, and from the corresponding 4-amino-1,2,4-triazole-3-thione derivatives. Reagents and conditions: (**i**) pyridine, reflux; (**ii**) KOH (10 eq.), EtOH, H_2_O, RT.

To prevent any side reactions, a selective pathway was developed through a two-step procedure from the 4-amino-triazole-thione compounds (Scheme 2). The first step consisted of the condensation between the 4-amino group and cyclic acid anhydrides (phthalic or succinic anhydrides) in refluxing pyridine to give the corresponding succinimide intermediates **13**–**16**. No by-products were observed in these conditions. In the second step, the imide ring was opened under basic conditions in a mixture of ethanol and water, at room temperature, leading to the hydrazide-like compounds **17**–**20** after an acidic workup procedure.

All synthetic compounds were characterized by NMR spectroscopy, LCMS, and HRMS. More than 95% of purity was determined by HPLC for each final compound. The procedures, yields, and physicochemical properties are described in the Supporting Information.

### 3.2. MBL Inhibition

The inhibitory potency of all synthesized compounds was first assessed against a representative panel of three different MBLs including the three most prevalent enzymes, IMP-1, VIM-2, and NDM-1. Inhibition data are shown in Table 1 and Table 2.

In the reduced hydrazone series, as a proof of concept, compound **1** (phenyl in 5, *o*-benzoic in 4), the reduced analog of one of the best previously described Schiff base compounds JMV4390 (Figure 1) [37], was evaluated first and showed a similar inhibitory activity on VIM-2 (1.4 vs. 2.7 μM). Nevertheless, compound **1** was inactive on NDM-1, which is in contrast with the activity of the parent Schiff base (JMV4390). In order to investigate the structure–activity relationships and to further assess the effect of hydrazone-like reduction, a focused library of compounds has been designed based on a panel of hydrazone-like compounds that we previously reported to possess different spectra of inhibition (i.e., significant activity toward 0, 1, 2, or 3 among the tested MBLs) [37]. Most compounds (i.e., **1**–**8**, **11**, and **12**) possessed an *ortho*-benzoyl group as B ring, and therefore only differed by their substituent at position 5. This substituent was an aromatic group either directly attached to the triazole ring (**1**–**5**) or separated by one (**6**, **7**) or two (**8**, **11**, **12**) atom(s) linker. The introduction of a methyl group at the *ortho* position of the phenyl ring (compound **2**) slightly decreased the VIM-2 inhibitory potency compared to compound **1**. Compound **2** also behaved as its hydrazone-like counterpart, which was moderately or not active against IMP-1 and NDM-1, respectively [37]. In the hydrazone-like series, we previously demonstrated that increasing the size of the aryl substituent at position 5 led to stronger VIM-2 inhibition and a broader spectrum of inhibition toward several MBLs [37]. Compounds **3** (*meta*-biphenyl) and **4** (naphth-2-yl) indeed inhibited VIM-2, with *K*_i_ values in a similar range as those measured for the corresponding hydrazone-like analogs. The activity against IMP-1 and NDM-1 was slightly decreased (compound **3**, 76 vs. 17 μM and 8.2 vs. 4.2 μM on IMP-1 and NDM-1, respectively) or lost (compound **4**). A similar result was obtained for the indole-containing compound **5**.

Separating the aryl substituent in 5 by one methylene group (compounds **6** and **7**) showed similar activities against VIM-2 compared to the corresponding analogs **1** and **4**, respectively, and to their Schiff base analogs. However, again, reducing the hydrazone-like bond led to the loss of IMP-1 and NDM-1 inhibition. In particular, compound **7** (naphtha-2-ylmethyl) strongly inhibited VIM-2 only, whereas its hydrazone-like counterpart showed a broad spectrum of inhibition. Separating the 5-aryl substituent by an additional atom was even more disappointing as compounds **8** (phenethyl), **11** (benzhydrylmethyl), and **12** (naphth-2-yloxymethyl) were inactive, with the exception of **11**, which moderately inhibited NDM-1. In addition, changing the 4-substituent in compound **8** by an *o*-nitrophenyl (**9**) or a *p*-benzoic (**10**) moiety was inefficient.

Overall, replacing the hydrazone-like group of Schiff base inhibitors [37] with its reduced form resulted in a narrower spectrum of inhibition, with VIM-2 being the only enzyme among the three most clinically relevant MBLs to be inhibited by compounds **1**–**4**, **6** and **7**, with the exception of NDM-1 for compound **3** (*K*_i_ value of 8.2 μM). In fact, whereas several Schiff base analogs were broad-spectrum MBL inhibitors, significant inhibition of VIM enzymes was more frequently observed than inhibition of IMP-1 or NDM-1 in this previous series [37]. In addition, in the Schiff base series, IMP-1 inhibition was highly dependent on the nature of the aryl substituent at position 4, and an *o*-benzoic moiety mostly yielded inactive or moderately potent IMP inhibitors. This likely reflects the different organization of the residues constituting the L1 loop, whose structure data revealed to be very different between IMP-type enzymes and VIM- and NDM-type enzymes. In the case of NDM-1, potent inhibition in the Schiff base series was also dependent on the size of the substituent at position 5, with biaryl moieties being highly favorable. Unfortunately, the presence of such groups in the reduced series (i.e., compounds **3**–**5**, **7**, **11**, and **12**) was efficient only in the case of a *m*-biphenyl (compound **3**) and, in a more limited extent, a benzhydrylmethyl (compound **11**) substituent. Overall, compound **3** was the most potent compound on both enzymes in this series.

Hydrazide-containing compounds **17**–**20** were also evaluated against the same MBLs (Table 2). Only compound **19** with a *m*-biphenyl substitution at position 5 showed broad-spectrum, though moderate, inhibition of all tested enzymes. Compared to compound **17**, this result supported the interest of a biaryl substituent at this position, but the hydrazone-to-hydrazide change led to drastic decrease in activity (see activities of the corresponding Schiff bases in Table 1). The alkanoic analog **20** showed no significant activity, indicating that the aryl group was involved in binding and/or in correctly orienting the carboxylic acid function into the active site. The global lack of inhibition of the hydrazide series might be explained by a modification of molecule conformation due to the constraint provided by the carbonyl group (see below). To note, the succinimide intermediates **13**–**16** were also tested but no significant inhibition could be measured.

The most active inhibitors were subjected to further characterization, including for their inhibition of additional MBLs, the VIM-type MBLs variants VIM-1 and VIM-4, the subclass B2 *Aeromonas hydrophila* CphA, and the subclass B3 *Stenotrophomonas maltophilia* L1 (compounds **1**–**4**, **6**, **7**, and **19**, Table 3). Compound **1** only showed moderate activity against the B2 enzyme CphA, and therefore appeared rather selective toward VIM-2. The lack of inhibition against VIM-1 and VIM-4 (a Ser228 Arg variant of VIM-1) is likely due to the different nature of second shell residues (mainly at positions 224 and 228) whose role in substrate recognition and catalysis was previously investigated [41,50]. Compound **2** showed a slightly broader spectrum as it inhibited both VIM-2 and VIM-4 with similar potencies, while being a modest inhibitor of CphA and L1 enzymes (Table 3). Compounds **3** and **7** also significantly inhibited VIM-1 and VIM-4 but to a lesser extent than their Schiff base analogs [37]. Compound **4** was also found to moderately inhibit VIM-4. Overall, the detrimental effect of Schiff base reduction was also observed for inhibition of these MBLs. Finally, the hydrazide-like compound **19** moderately inhibited VIM-4 in the same range as IMP-1, VIM-2, and NDM-1 enzymes. Compound **3** remarkably showed the lowest *K_i_* values for the clinically relevant NDM-1, VIM-1, VIM-2, and VIM-4 enzymes, while showing moderate inhibition of IMP-1. This broad spectrum of inhibition potentially relies in the increased hydrophobicity of its biphenyl substituent, likely to create favorable interactions with the L1 loop residues which define a hydrophobic patch on one side of the active site in subclass B1 metallo-β-lactamases [45,51].

### 3.3. Microbiological Assays

The ability to restore the activity of β-lactam antibiotics was evaluated on selected VIM-2 inhibitors **1**–**4**, **6**, and **7**, showing *K*_i_ values ranging 0.24 to 8.5 3M, using a disk diffusion assay. This assay was performed on an isogenic VIM-2-producing laboratory *Escherichia coli* strain, obtained by transforming the LZ2310 strain with the pLB-II plasmid into which the *bla*_VIM-2_ gene was cloned [45]. This strain is a triple knockout mutant of K12 in which genes encoding components of the major efflux pumps NorE, MdfA, and AcrA were inactivated. This microbiological assay was chosen to limit the potential efflux of the compounds, which could impair sufficient accumulation in the periplasm. A variable quantity of the compounds was added to a 30 μg cefoxitin disk and the inhibition zone diameter was measured (Table 4). The metal chelator EDTA was used as the positive control. None of the tested compounds showed intrinsic antibacterial activity.

Whereas the less potent VIM-2 inhibitors **2**, **4**, **6**, and **7** did not show any remarkable activity, compound **1** showed a dose-dependent increase of the antibiotic activity and was even able to restore cefoxitin susceptibility (according to EUCAST clinical breakpoints) when tested at 48 μg. The activity of compound **1** was similar to that exhibited by its Schiff base analog JMV4390 [37], indicating that the introduction of a stable linker in place of the hydrazone-like function did not affect the activity in whole cell assays. However, and despite being a more potent VIM-2 inhibitor, compound **3** was devoid of activity. A similar lack of restoration activity was observed when tested on a VIM-4-producing laboratory strain, while also exhibiting a low *K_i_* value (0.57 μM) on that enzyme. It is noteworthy that a similar result was obtained in the Schiff base series as substituting the 5-phenyl group of JMV4390 generally abolished the activity in this test [37]. Overall, these results suggest that the main issue would rely in the limited ability of these compounds to penetrate through the bacterial outer membrane and efficiently accumulate in the periplasm, as is often the case in Gram-negative pathogens. Further chemical modifications are currently being explored on this scaffold to potentially identify compounds with substantially improved in vitro activity in cell-based assays.

### 3.4. Isothermal Titration Calorimetry

**Figure 2:**
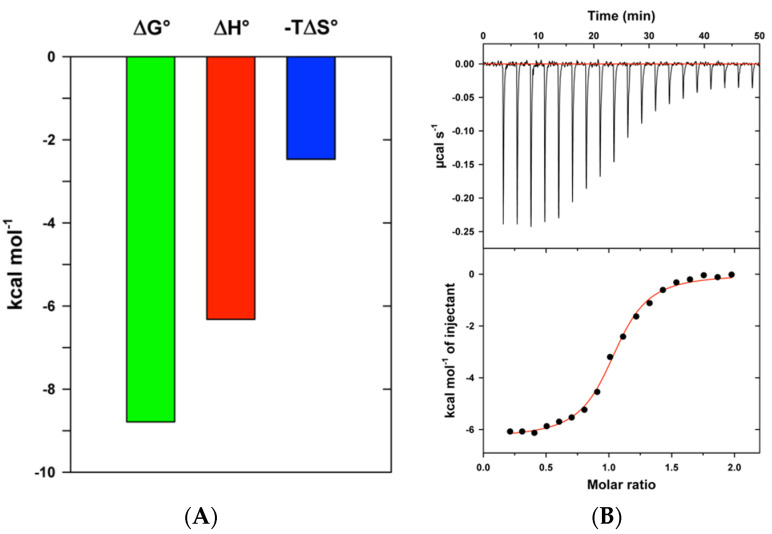
(**A**) VIM-2 binding energetics of compound **1**. Data are from Table 5. (**B**) Isothermal titration calorimetry of VIM-2 by compound **1** at 25 °C. Upper panel: exothermic microcalorimetric trace of compound injections into VIM-2 solution (18.7 µM). Lower panel: Wiseman plot of heat releases versus molar ratio of injectant/protein in the cell and nonlinear fit of the binding isotherm for *n* equivalent binding sites. The binding enthalpy corresponds to the amplitude of the transition curve, *K_a_* is derived from the slope of the transition and the stoichiometry *n* is determined at the transition midpoint.

To validate the binding mode of compounds to VIM-2, isothermal titration calorimetry was performed with compound **1**. The thermodynamic parameters of binding are given in Table 5 and are also depicted in Figure 2A for a more visual inspection. An ITC thermogram is shown in Figure 2B.

The experiment showed a stoichiometric relationship between VIM-2 and the inhibitor, which clearly indicated a 1/1 association. Compound **1** mainly behaved as a Schiff base containing the same *o*-benzoic 5-substituent. Its binding was enthalpy-driven (the enthalpic term is the major contributor to *∆G°_b_* and therefore to affinity). The favorable enthalpy contribution is an indication of specific interactions between binding partners and reflects ligand specificity and selectivity. Compound **1** also displayed a favorable entropic contribution, which is regarded as resulting from the release of organized water molecules at the surface of individual partners to the bulk solvents. Overall, this suggests that favorable enthalpic interactions, mainly electrostatic, occurring in the complex were replacing preexisting bonds established with the solvent water [52,53]. Accordingly, the net enthalpic effect is reduced due to desolvation and, as a result, the affinity remained moderate.

### 3.5. Modeling Study

To help rationalize the relative VIM-2 inhibitory activities of hydrazone- vs. hydrazine- vs. hydrazide-containing compounds, we investigated the putative binding mode of the hydrazone JMV4390, the hydrazine **1** (JMV5062), and the hydrazide **17** (JMV7100) within VIM-2 active site using molecular modeling experiments.

The docking experiments were performed with a VIM-2 model generated from 6 YRP available in the Protein data Bank [37] using AutoDock 4.2. 6 YRP is the crystallographic structure of the complex formed between VIM-2 and the hydrazone-like compound JMV4690 (*K*_i_ = 0.7 μM), which differs from JMV4390 by possessing a 2-hydroxy-5-methoxy-phenyl group at position 5 (Figure S1). In addition to the double coordination of the active site zinc ions by the triazole-thione moiety, this structure is characterized by several H bonds involving the two carboxylate oxygens, which interact with the main chain NH of Asn233 and two structural waters (named 5 and 177), and the methoxy group, which interacts with the indole NH of Trp87. π-stacking interactions were also observed between the 5- and 4-aromatic rings and Trp87 and His263, respectively.

**Figure 3:**
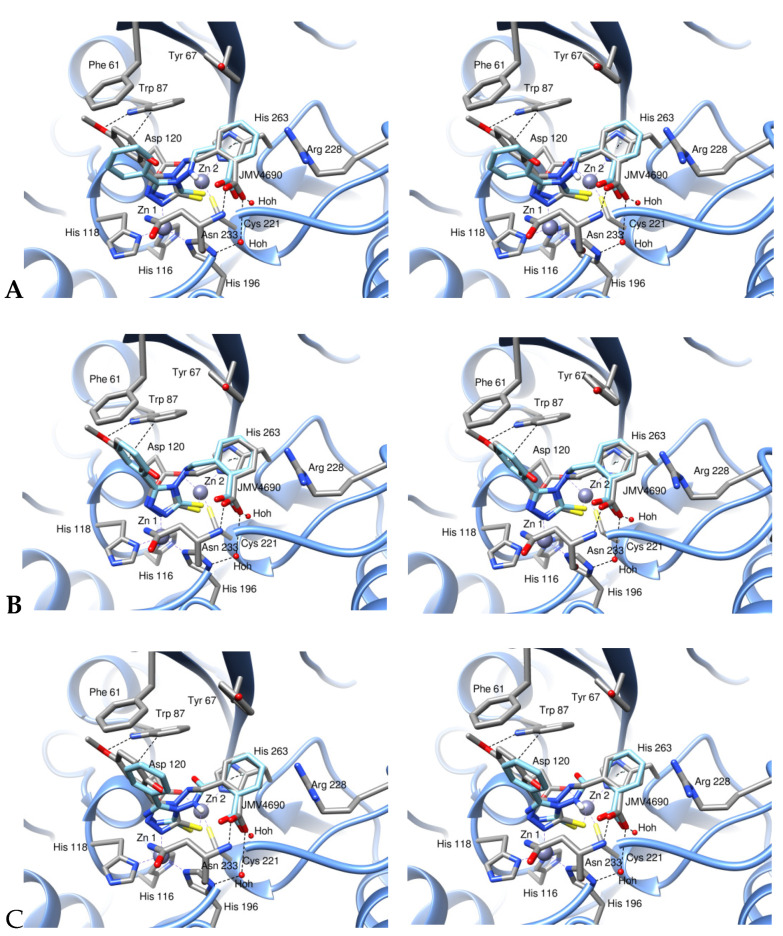
Stereo views showing the binding mode of compounds JMV4390 (**A**), **1** (**B**), and **17** (**C**) in VIM-2 studied by molecular modeling. Compounds (light blue) were superimposed with JMV4690 for comparison. In the case of compound **17**, this pose shows the expected positioning of the carboxylate group within the water pocket but the rigid hydrazide does not allow the N^2^ atom of the triazole to correctly coordinate Zn1 (distance of 2.44 Å instead of 1.96–2.19 Å for other compounds, Table S1). The images were produced using UCSF Chimera [54].

As none of the docked compounds possess a methoxy group on the 5-phenyl substituent, no H bond could be established with Trp87, probably explaining the superiority of JMV4690 in VIM-2 inhibition. However, apart from this, both compounds, JMV4390 and **1**, could bind in the VIM-2 active site as JMV4690 with their triazole-thione moiety double-coordinating the two zinc ions and their carboxylate group being involved in a H bond network with Asn233 and two water molecules (Figure 3A,B). In contrast, the rigid hydrazide-like bond of compound **17** did not allow to simultaneously establish these two sets of essential interactions (Figure 3C), explaining the low inhibitory activity of this compound. The Table S1 presents the free energies of best poses and estimated *K*_i_ values for all four compounds, which were globally consistent with experimental values.

## 4. Conclusions

The 1,2,4-triazole-3-thione heterocycle has emerged as an effective and original Zn-binding scaffold for the design of MBL inhibitors, supported by its specific double Zn coordination [34]. Compared to usual Zn ligands (e.g., thiol), this scaffold presents higher hindrance and lower flexibility because of the double coordination and is not as strong a Zn^2+^ ligand. These properties should offer a higher probability to spare human metalloenzymes. Furthermore, dizinc enzymes are much less abundant in humans than monozinc ones. Originally identified from structure-based pharmacophore design [33], several experimental or in silico library screenings randomly retrieved this heterocycle [55,56,57], and various series have been reported [35,37,58,59,60]. *S*-Alkylated analogs have also been developed, but with a different binding mode because of masked sulfur [61,62]. To date, the most potent series was the Schiff base one [37], which afforded several micromolar to sub-micromolar broad-spectrum inhibitors of MBLs, including IMP-1, VIM-2, and NDM-1. Although some compounds showed an interesting ability to restore the activity of a β-lactam antibiotic in in vitro antibacterial susceptibility assays on MBL-producing strains, potential issues, such as poor penetration and/or hydrolytic susceptibility of their hydrazone-like bond, could also represent a limitation of the Schiff base analogs. To address this issue, we first reduced the hydrazone to hydrazine, this reduction being possible only with Schiff bases bearing aryl moieties with electron withdrawing groups. Fortunately, it includes the most favorable substituent at position 4 (i.e., *o*-benzoic). Overall, going from hydrazone to hydrazine led to a general decrease in inhibition potency against all tested MBLs, with the exception of VIM-2 and VIM-4, therefore narrowing the inhibition spectrum to these enzymes. This effect obviously came from the different geometry and flexibility of hydrazine vs. hydrazone link. An exception was compound **3**, which, in addition to VIM-2 and VIM-4, also significantly inhibited VIM-1 and NDM-1. Finally, the substitution of the hydrazone-like bond by a hydrazide-like function led to a poorly active series, although the geometry of the two moieties was expected to be similar. The modeling study suggested that, in contrast to the hydrazine analogs, the rigid hydrazide compounds could not establish both essential interactions made by a hydrazone inhibitor in VIM-2 active site.

Nevertheless, this study led to the identification of a potent and selective inhibitor of VIM-2 (**1**) able to restore the susceptibility of a VIM-2-producing recombinant *E. coli* strain to a cephalosporin. Novel insights into the structural requirements for the design of MBLs inhibitors have been reached thanks to binding mode studies by ITC and modeling study. Based on these results and previous studies [35,37], the development of new stable inhibitors with different links is ongoing and will be reported in due course.

## Figures and Tables

**Table 1:** Inhibitory potencies of compounds **1**–**12** of the hydrazine series against representative MBLs ^1^.

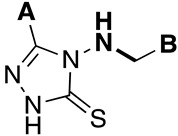			*K*_i_ Value (μM)		
Compound	A	B	IMP-1	VIM-2	NDM-1
**1**	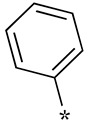	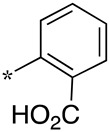	-^2^ *(-)*	1.40 ± 0.02 *(2.7)*	- *(38)*
**2**	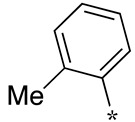	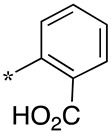	- *(58)*	8.0 ± 0.7 *(3.0)*	- *(-)*
**3**	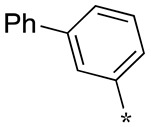	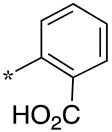	76 ± 13 *(18)*	0.24 ± 0.02 *(0.20)*	8.2 ± 0.7 *(4.2)*
**4**	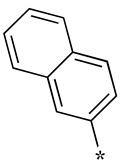	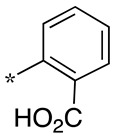	- *(6.9)*	4.2 ± 0.9 *(0.7)*	- *(2.3)*
**5**	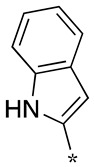	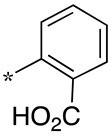	- *(55)*	- *(-)*	- *(9.4)*
**6**	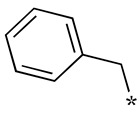	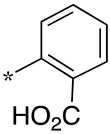	- *(28)*	8.5 ± 2.3 *(3.2)*	- *(42)*
**7**	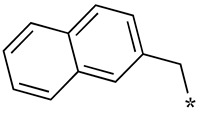	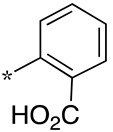	- *(34)*	1.8 ± 0.2 *(2.4)*	- *(1.6)*
**8**	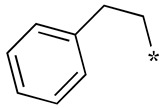	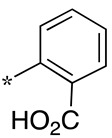	- *(-)*	- *(7.0)*	- *(-)*
**9**	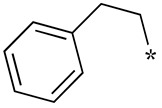	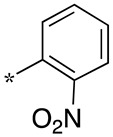	- *(-)*	- *(-)*	- *(-)*
**10**	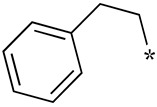	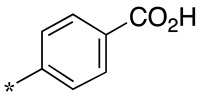	- *(11)*	- *(-)*	- *(-)*
**11**	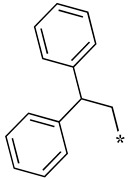	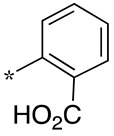	- *(-)*	- *(25)*	16.4 ± 2.6 *(2.7)*
**12**	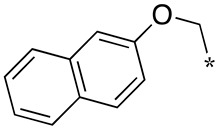	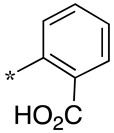	- *(-)*	- *(2.0)*	- *(2.4)*

^1^*K*_i_ values for the corresponding hydrazone-like compounds are presented between parentheses and in italics for comparison (data from the work in [37], except for analogs of compounds **5** and **6** (this work)). ^2^ <40% of inhibition in the presence of 100 μM inhibitor.

**Table 2:** Inhibitory potencies of compounds **17**–**20** of the hydrazide-like series against representative MBLs.

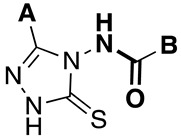			Percentage of Inhibition at 100 μM Inhibitor ^1^		
Compound	A	B	IMP-1	VIM-2	NDM-1
**17**	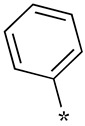	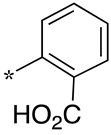	-^2^	-	-
**18**	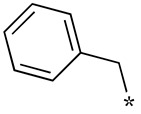	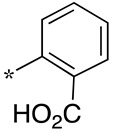	-	-	-
**19**	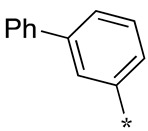	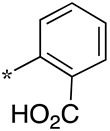	40	36	42
**20**	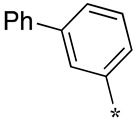	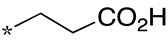	-	-	-

^1^ All experiments were performed in triplicate. ^2^ <20% of inhibition in the presence of 100 μM inhibitor.

**Table 3:** Inhibitory potencies of compounds **1**–**4**, **6**, **7**, and **19** against additional MBLs.

Compounds	*K*_i_ Values (μM) or Percentage of Inhibition at 100 μM Inhibitor			
	VIM-1	VIM-4	CphA	L1
**1**	-^1^	-	60%	-
**2**	-	10 ± 1	100 ± 10	110 ± 10
**3**	3.6 ± 0.2	0.57 ± 0.03	nd	nd
**4**	32%	6.3 ± 0.7	nd	-
**6**	-	-	nd	-
**7**	19.5 ± 1.3	4.7 ± 0.3	nd	nd
**19**	-	41%	nd	nd

^1^ <30% of inhibition in the presence of 100 μM inhibitor. nd: Not Determined. All the experiments were performed in triplicate.

**Table 4:** In vitro activity of selected VIM-2 inhibitors measured by disk diffusion on *E. coli* LZ2310(pLBII-VIM-2) ^1^.

Compound Added to Cefoxitin Disk ^2^	*K*_i_ (μM) on VIM-2	Inhibition Zone Diameter (mm) ^3^
none	-	10
EDTA (220 μg)	-	28
DMSO (100%)	-	10
**1** (16 μg)	1.4	13
**1** (32 μg)	1.4	16
**1** (48 μg)	1.4	20
**2** (40 μg)	8.0	11
**3** (120 μg)	0.24	11
**4** (120 μg)	4.2	10
**6** (40 μg)	8.5	10
**7** (120 μg)	1.8	11

^1^ The recombinant MBL-producing *E. coli* was obtained by transforming strain LZ2310 (triple knockout mutant of efflux pumps) with a pLB-II-derivative plasmid carrying the cloned *bla*_VIM-2_ gene (the parental strain was fully susceptible to cefoxitin: inhibition zone diameter, 28 mm). ^2^ The compounds were dissolved in DMSO. DMSO was used as a control and did not affect the diameter of the growth inhibition zone. Two-hundred-and-twenty micrograms of EDTA restored full susceptibility to the antibiotic. When tested alone, the compounds did not show any antibacterial activity. ^3^ EUCAST resistance breakpoint = 19 mm.

**Table 5:** Thermodynamic parameters of compound **1** binding to VIM-2 at 25 °C.

Compound	n ^1^	K_a_ (μM^−1^)	K_d_ (nM)	∆G°_b_ (kcal.mol^−1^)	∆H°_b_ (kcal.mol^−1^)	T∆S°_b_ (kcal.mol^−1^)
**1**	1.01 ± 0.01	2.8 ± 0.4	358	−8.8	−6.3 ± 0.1	2.5

^1^ n, stoichiometry of binding.

## Supplementary Materials

The following are available online at https://www.mdpi.com/2218-273X/10/8/1094/s1, NMR and MS characterization of compounds; Figure S1: Crystallographic structure of the complex between VIM-2 and JMV4690 used for docking experiments and docking of JMV4690; Table S1: Docking data for compounds JMV4690, 4390, **1** and **17**; ^1^H and ^13^C NMR spectra of molecules.
