# Supplementary material for: 4-(N-Alkyl- and -Acyl-amino)-1,2,4-triazole-3-thione Analogs as Metallo-β-Lactamase Inhibitors: Impact of 4-Linker on Potency and Spectrum of Inhibition
Source: Biomolecules. 2020 Jul 23;10(8):1094. doi: 10.3390/biom10081094 (PMC7465886; doi:10.3390/biom10081094)
Supplement: Supplementary file 1 [file biomolecules-10-01094-s001.pdf]

Supporting Information

4-(*N*-Alkyl- and *N*-acyl-amino)-1,2,4-triazole-3-thione analogues as metallo-β-lactamase inhibitors: impact of 4-linker on potency and spectrum of inhibition

Table of content

General methods………2

NMR and MS characterization of compounds……………………………………………………………………….2

Figure S1…….7

Table S1…….7

^{1}H and ^{13}C NMR spectra……………………………………………………………………………………………8

General methods

NMR. ^{1}H NMR and ^{13}C NMR spectra were recorded with a 400 or 500 MHz instrument in [D$_6$]DMSO solutions unless otherwise indicated. Splitting patterns in the ^{1}H NMR spectra are designated as follows: s, singlet; d, doublet; t, triplet; q, quartet; m, multiplet; br, broad. Samples for reverse-phase (RP) HPLC were prepared in an acetonitrile / water (50:50 v/v) mixture. **IR spectra** were collected on a Perkin Elmer Spectrum One apparatus. **RP HPLC** analyses were performed with a Chromolith SpeedRod C18 column (0.46x5 cm) by means of a linear gradient (0–100%) of 0.1% trifluoroacetic acid (TFA)/acetonitrile in 0.1% aqueous TFA over 5 min at a flow rate of 3 mL.min^{-1}. **LC–MS** analyses were performed with a Waters Alliance 2690 HPLC coupled to a Waters-Micromass ZQ spectrometer (electrospray ionization mode, ESI+). Samples were separated by using a RP C18 monolithic Onyx Phenomenex 2.5x0.46 cm column. The flow rate was set to 3 mL.min^{-1} with eluent A (water/0.1% formic acid), and a gradient of 0 to 100% of eluent B (acetonitrile/ 0.1% formic acid) in A over 3 min was then applied. Positive-ion electrospray mass spectra were acquired at a solvent flow rate of 100–500 µL.min^{-1}. Nitrogen was used as both the nebulizing and drying gas. The data were obtained in a scan mode in 0.1 s intervals; 10 scans were summed up to get the final spectrum. **TLC.** Thin-layer chromatography was performed on aluminum-backed sheets of silica gel F254 (0.2 mm), which were visualized under v=254 nm light. **Purification.** Column chromatography was performed by using Merck silica gel 60 of particle size 40– 63 mm. **HR-MS** were registered on a JEOL JMS-SX-102A mass spectrometer. **Mp.** Melting points were determined on a capillary melting point apparatus.

Physico-chemical properties of compounds

Compounds 1-12 derived from hydrazone reduction

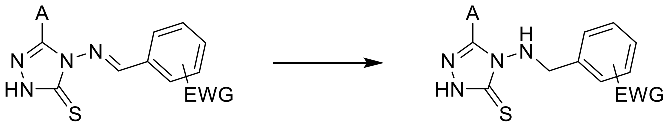

1-12

2-{[(3-Phenyl-5-sulfanylidene-4,5-dihydro-1H-1,2,4-triazol-4-yl)amino]methyl}benzoic acid - 1: White powder; Yield: 69%; LC-MS t_R: 1.45 min; *m/z* (ES+) 327.1 (M+H$^+$); Mp = 161-162 °C; IR (ATR): 3082, 2932, 1683 cm^{-1}; ^{1}H NMR (400 MHz, DMSO-*d*$_6$): 13.92 (bs, 1H), 12.94 (bs, 1H), 7.88-7.84 (m, 2H), 7.73 (dd, *J* = 8, 1.2 Hz, 1H), 7.51-7.36 (m, 4H), 7.30 (td, *J* = 7.6, 1.2 Hz, 1H), 7.19 (d, *J* = 7.6 Hz, 1H), 6.77 (t, *J* = 4 Hz, 1H), 4.55 (d, *J* = 4 Hz, 2H); ^{13}C NMR (100 MHz, DMSO-*d*$_6$): 168.1, 166.2, 148.8, 136.9, 131.5, 130.7, 130.4, 130.3 (2C), 128.3, 127.5, 127.5, 125.2, 50.8; HRMS (ESI+) calcd for $C_{16}H_{15}N_4O_2S$ (M+H)$^+$ 327.0916, found 327.0919.

***o*-{[5-Thioxo-3-(*o*-tolyl)-1,4-dihydro-1,2,4-triazol-4-ylamino]methyl}benzoic acid - 2:** White powder; Yield: 35%; LC-MS t_R: 1.47 min; *m/z* (ES+) 341.2 (M+H$^+$); Mp = 193-194 °C; IR (ATR): 3269, 2826, 1682 cm^{-1}; ^{1}H NMR (500 MHz, DMSO-*d*$_6$): 13.86 (bs, 1H), 12.77 (bs, 1H), 7.70-7.66 (m, 1H), 7.38 (tt, *J* = 7,

1.5 Hz, 1H); 7.29-7.18 (m, 5H), 6.93-6.90 (m, 1H), 6.78 (bs, 1H), 4.39 (s, 2H), 2.12 (s, 3H); ^{13}C NMR (125 MHz, DMSO-d_6): 168.1, 165.4, 149.9, 137.4, 137.3, 131.2 (2C), 130.8, 130.2, 130.1 (2C), 130.0, 127.3, 125.4, 124.8, 50.9, 19.4; HRMS (ESI+) calcd for $C_{17}H_{17}N_4O_2S$ $(M+H)^+$ 341.1067, found 341.1073.

***o*-{[3-(3-Biphenylyl)-5-thioxo-1,4-dihydro-1,2,4-triazol-4-ylamino]methyl}benzoic acid - 3:** White powder; Yield: 81%; LC-MS t_R: 1.76 min; *m/z* (ES+) 403.0 $(M+H^+)$; Mp = 193-194 °C; IR (ATR): 3116, 2948, 1680 cm^{-1}; ^{1}H NMR (500 MHz, DMSO-d_6): 13.98 (bs, 1H), 12.94 (bs, 1H), 8.08 (t, *J* = 1.5 Hz, 1H), 7.83 (dt, *J* = 7.5, 1.5 Hz, 1H), 7.77-7.71 (m, 2H), 7.58-7.19 (m, 9H), 6.87 (t, *J* = 6.0 Hz, 1H), 4.60 (d, *J* = 6.0 Hz, 2H); ^{13}C NMR (125 MHz, DMSO-d_6): 168.0, 166.4, 148.8, 140.2, 139.2, 137.2, 131.6, 130.4, 130.3, 130.2, 129.1, 128.9, 128.6, 127.7, 127.4, 126.7, 126.6, 125.9, 125.7, 50.9; HRMS (ESI+) calcd for $C_{22}H_{19}N_4O_2S$ $(M+H)^+$ 403.1229, found 403.1233.

2-({[3-(Naphthalen-2-yl)-5-sulfanylidene-4,5-dihydro-1H-1,2,4-triazol-4-yl]amino}methyl)benzoic acid - 4: White powder; Yield: 45%; LC-MS t_R: 1.65 min; *m/z* (ES+) 377.2 $(M+H^+)$; Mp = 213-214 °C; IR (ATR): 3216, 2921, 2849, 1687 cm^{-1}; ^{1}H NMR (400 MHz, DMSO-d_6): 14.01 (bs, 1H), 12.98 (bs, 1H), 8.60 (s, 1H), 7.94 (bs, 3H), 7.89 (d, *J* = 8.8 Hz, 1H), 7.70 (d, *J* = 8.8 Hz, 1H), 7.63-7.54 (m, 2H), 7.39-7.34 (m, 1H), 7.29-7.23 (m, 2H), 6.90-6.84 (m, 1H), 4.63 (d, *J* = 5.2 Hz, 2H); ^{13}C NMR (100 MHz, DMSO-d_6): 168.1, 166.5, 148.6, 137.1, 133.2, 132.0, 131.5, 130.8, 130.6, 130.3, 128.6, 127.9, 127.6, 127.5, 127.4, 127.3, 126.6, 124.1, 122.6, 50.9; HRMS (ESI+) calcd for $C_{20}H_{17}N_4O_2S$ $(M+H)^+$ 377.1072, found 377.1070.

***o*-{[3-(1*H*-Indol-2-yl)-5-thioxo-1,4-dihydro-1,2,4-triazol-4-ylamino]methyl}benzoic acid - 5:** White powder; Yield: 47%; LC-MS t_R: 1.60 min; *m/z* (ES+) 366.1 $(M+H^+)$; Mp = 242-243 °C; ^{1}H NMR (500 MHz, DMSO-d_6): 14.01 (bs, 1H), 13.15 (bs, 1H), 11.69 (bs, 1H), 7.84 (d, *J* = 7.0 Hz, 1H), 7.57-7.42 (m, 4H), 7.37 (t, *J* = 7.5 Hz, 1H), 7.27 (d, *J* = 1.0 Hz, 1H), 7.19 (t, *J* = 7.5 Hz, 1H), 7.03 (t, *J* = 7.5 Hz, 1H), 6.84 (t, *J* = 7.0 Hz, 1H), 6.53 (s, 1H), 4.69 (d, *J* = 5.5 Hz, 2H); ^{13}C NMR (125 MHz, DMSO-d_6): 168.4, 166.2, 143.9, 137.0, 136.6, 131.8, 130.7, 130.4, 130.3, 127.8, 127.1, 123.4, 122.2, 121.1, 119.1, 111.8, 104.1, 50.7; HRMS (ESI+) calcd for $C_{18}H_{16}N_5O_2S$ $(M+H)^+$ 366.1025, found 366.1028.

2-{[(3-Benzyl-5-sulfanylidene-4,5-dihydro-1H-1,2,4-triazol-4-yl)amino]methyl}benzoic acid - 6: White powder; Yield: 89%; LC-MS t_R: 1.51 min; *m/z* (ES+) 341.2 $(M+H^+)$; Mp = 158-159 °C; ^{1}H NMR (400 MHz, DMSO-d_6): 13.56 (bs,1H), 13.11 (bs, 1H), 7.85 (dd, *J* = 7.6, 1.2 Hz, 1H), 7.51 (td, *J* = 7.6, 1.2 Hz, 1H), 7.43 (td, *J* = 7.6, 1.2 Hz, 1H), 7.31-7.18 (m, 4H), 7.15-7.10 (m, 2H), 6.53 (bs, 1H), 4.56 (s, 2H), 3.68 (s, 2H); ^{13}C NMR (100 MHz, DMSO-d_6): 168.5, 165.6, 151.6, 137.1, 135.2, 131.5, 130.9, 130.2, 128.7, 128.3, 127.8, 126.6, 99.4, 50.3, 29.6; HRMS (ESI+) calcd for $C_{17}H_{17}N_4O_2S$ $(M+H)^+$ 341.1072, found 341.1074.

***o*-({3-[(2-Naphthyl)methyl]-5-thioxo-1,4-dihydro-1,2,4-triazol-4-ylamino}methyl)benzoic acid - 7:** White powder; Yield: 47%; LC-MS t_R: 1.70 min; *m/z* (ES+) 391.1 $(M+H^+)$; Mp = 202-203 °C; ^{1}H NMR (500 MHz, DMSO-d_6): 13.60 (bs, 1H), 13.06 (bs, 1H), 7.87-7.77 (m, 4H), 7.62 (s, 1H), 7.52-7.41 (m, 4H), 7.31-7.24 (m, 2H), 6.57 (bs, 1H), 4.60 (s, 2H), 3.86 (s, 2H); ^{13}C NMR (125 MHz, DMSO-d_6): 168.6, 165.7, 151.6, 137.1, 132.8 (2C), 131.8, 131.6, 131.5, 131.0, 130.2, 127.8, 127.7, 127.4, 127.3, 127.2, 127.1, 126.1, 125.7, 50.3, 45.4; HRMS (ESI+) calcd for $C_{21}H_{19}N_4O_2S$ $(M+H)^+$ 391.1229, found 391.1230.

***o*-[(3-Phenethyl-5-thioxo-1,4-dihydro-1,2,4-triazol-4-ylamino)methyl]benzoic acid - 8:** White powder; Yield: 71%; LC-MS t_R: 1.58 min; *m/z* (ES+) 355.1 $(M+H^+)$; Mp = 164-165 °C; ^{1}H NMR (500 MHz, DMSO-d_6): 13.48 (bs, 1H), 13.06 (bs, 1H), 7.84 (d, *J* = 7.5 Hz, 1H), 7.52-7.39 (m, 2H), 7.33-7.09 (m, 6H),

6.55 (bs, 1H), 4.61(bs, 2H), 2.78 (t, *J* = 7.8 Hz, 2H), 2.56 (t, *J* = 7.8 Hz, 2H); ^{13}C NMR (125 MHz, DMSO-*d*$_6$): 168.5, 165.5, 151.8, 140.1, 137.4, 131.5 (2C), 131.0, 130.3, 128.2, 128.1, 127.8, 126.0, 50.4, 31.0, 25.3; HRMS (ESI+) calcd for $C_{18}H_{19}N_4O_2S$ $(M+H)^+$ 355.1229, found 355.1229.

4-{[(*o*-Nitrophenyl)methyl]amino}-5-phenethyl-2,4-dihydro-1,2,4-triazole-3-thione - 9 Light yellow powder; Yield: 66%; LC-MS t_R: 1.77 min; *m/z* (ES+) 356.0 $(M+H^+)$; Mp = 153-154 °C; ^{1}H NMR (500 MHz, DMSO-*d*$_6$): 13.54 (bs, 1H), 7.99 (td, *J* = 8.0, 1.0 Hz, 1H), 7.68 (td, *J* = 7.5, 1.0 Hz, 1H), 7.59 (t, *J* = 7.5 Hz, 1H), 7.51 (d, *J* = 7.5 Hz, 1H), 7.28-7.09 (m, 5H), 6.75 (t, *J* = 10.0 Hz, 1H), 4.59 (d, *J* = 10.0 Hz, 2H), 2.78 (t, *J* = 7.8 Hz, 2H), 2.55 (t, *J* = 7.8 Hz, 2H); ^{13}C NMR (125 MHz, DMSO-*d*$_6$): 166.3, 152.4, 149.8, 140.6, 133.9, 132.6, 131.6, 129.9, 128.8, 128.6, 126.6, 125.1, 49.4, 31.6, 25.8; HRMS (ESI+) calcd for $C_{17}H_{18}N_5O_2S$ $(M+H)^+$ 356.1181, found 356.1188.

***p*-[(3-Phenethyl-5-thioxo-1,4-dihydro-1,2,4-triazol-4-ylamino)methyl]benzoic acid - 10:** White powder; Yield: 55%; LC-MS t_R: 1.50 min; *m/z* (ES+) 355.1 $(M+H^+)$; Mp = 229-230 °C; ^{1}H NMR (500 MHz, DMSO-*d*$_6$): 13.55 (bs, 1H), 12.98 (bs, 1H), 7.90 (d, *J* = 8.4 Hz, 2H), 7.40 (d, *J* = 8.4 Hz, 2H), 7.24 (t, *J* = 7.5 Hz, 2H), 7.16 (t, *J* = 7.5 Hz, 1H), 7.07 (d, *J* = 7.2 Hz, 2H), 6.64 (t, *J* = 4.5 Hz, 1H), 4.34 (d, *J* = 4.5 Hz, 2H), 2.75 (t, *J* = 7.8 Hz, 2H), 2.53-2.49 (m, 2H); ^{13}C NMR (125 MHz, DMSO-*d*$_6$): 167.1, 165.6, 152.0, 141.5, 140.1, 129.4, 129.4, 129.3, 128.2, 128.1, 126.0, 51.6, 30.9, 25.5; HRMS (ESI+) calcd for $C_{18}H_{19}N_4O_2S$ $(M+H)^+$ 355.1229, found 355.1234.

2-({[3-(2,2-Diphenylethyl)-5-sulfanylidene-4,5-dihydro-1H-1,2,4-triazol-4-yl]amino}methyl)benzoic acid - 11: White powder; Yield: 45%; LC-MS t_R: 1.74 min; *m/z* (ES+) 431.2 $(M+H^+)$; Mp = 185-186 °C; ^{1}H NMR (400 MHz, DMSO-*d*$_6$): 13.38 (bs, 1H), 13.15 (bs, 1H), 7.86 (td, *J* = 7.6, 1.6 Hz, 1H), 7.48 (td, *J* = 7.4, 1.2 Hz, 1H), 7.34 (d, *J* = 7.2 Hz, 1H), 7.27-7.10 (m, 11H), 6.58 (bs, 1H), 4.63 (bs, 2H), 4.48 (t, *J* = 8.0 Hz, 1H), 3.03 (d, *J* = 8.0 Hz, 2H); ^{13}C NMR (100 MHz, DMSO-*d*$_6$): 168.5 ,165.4, 150.8, 143.4, 137.4, 131.5, 130.9, 130.2, 128.2, 127.8, 127.4, 127.3, 126.2, 50.4, 46.5, 29.3; HRMS (ESI+) calcd for $C_{24}H_{23}N_4O_2S$ $(M+H)^+$ 431.1542, found 431.1542.

***o*-({3-[(2-Naphthyloxy)methyl]-5-thioxo-1,4-dihydro-1,2,4-triazol-4-ylamino}methyl)benzoic acid - 12:** White powder; Yield: 58%; LC-MS t_R: 1.73 min; *m/z* (ES+) 407.0 $(M+H^+)$; Mp = 193-194 °C; ^{1}H NMR (500 MHz, DMSO-*d*$_6$): 13.87 (bs, 1H), 13.16 (bs, 1H), 7.85-7.77 (m, 4H), 7.48 (t, *J* = 7.5 Hz, 1H), 7.43-7.31 (m, 5H), 7.12 (dd, *J* = 9.0, 2.5 Hz, 1H), 6.80 (bs, 1H), 4.96 (s, 2H), 4.66 (s, 2H); ^{13}C NMR (125 MHz, DMSO-*d*$_6$): 168.6, 166.2, 155.1, 148.0, 137.1, 133.8, 131.5, 130.6, 130.2, 129.4 (2C), 128.7, 127.7, 127.4, 126.7, 126.4, 123.9, 118.2, 107.3, 58.7, 51.0; HRMS (ESI+) calcd for $C_{21}H_{19}N_4O_3S$ $(M+H)^+$ 407.1178, found 407.1182.

Compounds 13-16 derived from succinic anhydride condensation

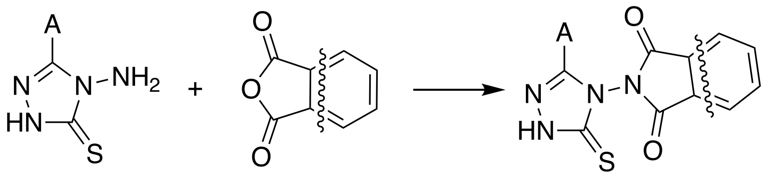

13 - 16

2-(3-Phenyl-5-thioxo-1,4-dihydro-1,2,4-triazol-4-yl)-2*H*-isoindole-1,3-dione - 13: White powder; Yield: 72%; LC-MS t_R: 1.60 min; *m/z* (ES+) 323.1 (M+H$^+$); Mp = 211-212 °C; ^{1}H NMR (500 MHz, DMSO-d_6): 14.78 (bs, 1H), 8.15-8.11 (m, 2H), 8.08-8.03 (m, 2H), 7.69-7.66 (m, 2H), 7.61-7.56 (m, 1H), 7.55-7.50 (m, 2H); ^{13}C NMR (125 MHz, DMSO-d_6): 167.1, 163.5, 150.2, 136.4, 131.8, 129.5, 128.9, 127.0, 125.0, 123.2; HRMS (ESI+) calcd for $C_{16}H_{11}N_4O_2S$ (M+H)$^+$ 323.0597, found 323.0605.

2-(3-Benzyl-5-thioxo-1,4-dihydro-1,2,4-triazol-4-yl)-2*H*-isoindole-1,3-dione - 14: White powder; Yield: 54%; LC-MS t_R: 1.59 min; *m/z* (ES+) 337.1 (M+H$^+$); Mp = 229-230 °C; ^{1}H NMR (500 MHz, DMSO-d_6): 14.34 (bs, 1H), 8.12-8.07 (m, 2H), 8.05-8.01 (m, 2H), 7.27-7.17 (m, 5H), 4.12 (s, 2H); ^{13}C NMR (125 MHz, DMSO-d_6): 166.3, 162.6, 151.8, 136.0, 134.1, 129.2, 128.8, 128.5, 127.1, 124.6, 29.5; HRMS (ESI+) calcd for $C_{17}H_{13}N_4O_2S$ (M+H)$^+$ 337.0754, found 337.0753.

2-[3-(3-Biphenylyl)-5-thioxo-1,4-dihydro-1,2,4-triazol-4-yl]-2*H*-isoindole-1,3-dione - 15: Pink powder, Yield: 62%; LC-MS t_R: 1.90 min; *m/z* (ES+) 399.1 (M+H$^+$); Mp = 199-200 °C; IR (ATR): 3034, 2947, 1751 cm^{-1}; ^{1}H NMR (500 MHz, DMSO-d_6): 14.81 (bs, 1H), 8.17-8.13 (m, 2H), 8.08-8.04 (m, 2H), 7.90-7.86 (m, 2H), 7.65-7.57 (m, 4H), 7.48-7.43 (m, 2H), 7.41-7.37 (m, 1H); ^{13}C NMR (125 MHz, DMSO-d_6): 167.1, 163.5, 150.2, 141.2, 138.6, 136.5, 130.4, 130.1, 129.1, 128.9, 128.2, 126.7, 125.9, 125.2, 125.0, 123.9; HRMS (ESI+) calcd for $C_{22}H_{15}N_4O_2S$ (M+H)$^+$ 399.0910, found 399.0916.

1-[3-(3-Biphenylyl)-5-thioxo-1,4-dihydro-1,2,4-triazol-4-yl]-2,5-pyrrolidinedione - 16: Off-white powder; Yield: 67%; LC-MS t_R: 1.66 min; *m/z* (ES+) 351.1 (M+H$^+$); Mp = 221-222 °C; ^{1}H NMR (500 MHz, DMSO-d_6): 14.71 (bs, 1H), 7.98-7.92 (m, 1H), 7.79-7.76 (m, 1H), 7.68-7.65 (m, 3H), 7.53-7.47 (m, 3H), 7.45-7.41 (m, 1H), 3.08 (bs, 4H); ^{13}C NMR (125 MHz, DMSO-d_6): 173.6, 172..6, 166.8, 149.7, 141.3, 138.7, 130.4, 129.2, 128.2, 126.8, 125.7, 125.2, 123.9, 28.7, 26.8; HRMS (ESI+) calcd for $C_{18}H_{15}N_4O_2S$ (M+H)$^+$ 351.0910, found 351.0924.

Compounds 17-20 obtained after succinimide ring-opening reaction

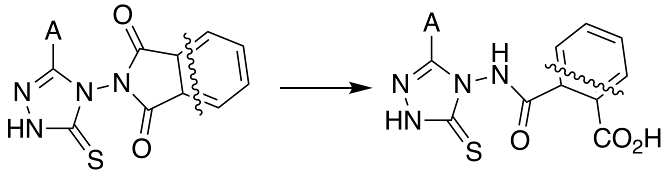

13 - 16 **17 - 20**

***o*-[(3-Phenyl-5-thioxo-1,4-dihydro-1,2,4-triazol-4-ylamino)carbonyl]benzoic acid - 17:** White powder; Yield: 11%; LC-MS t_R: 1.13 min; *m/z* (ES+) 341.0 (M+H$^+$); Mp = 177-178 °C; ^{1}H NMR (500 MHz, DMSO-d_6): 14.19 (bs, 2H), 13.31 (bs, 1H), 8.16-8.12 (m, 1H), 7.92-7.85 (m, 3H), 7.71-7.59 (m, 2H), 7.57-7.48 (m, 3H); ^{13}C NMR (125 MHz, DMSO-d_6): 167.8 (2C), 167.6, 150.3, 131.8, 130.9, 130.6, 130.0, 129.4, 128.9, 128.8, 128.5, 127.5, 124.5; HRMS (ESI+) calcd for $C_{16}H_{13}N_4O_3S$ (M+H)$^+$ 341.0703, found 341.0707.

***o*-[(3-Benzyl-5-thioxo-1,4-dihydro-1,2,4-triazol-4-ylamino)carbonyl]benzoic acid - 18:** White powder; Yield: 27%; LC-MS t_R: 1.19 min; *m/z* (ES+) 355.1 (M+H$^+$); Mp > 250 °C; ^{1}H NMR (500 MHz, DMSO-d_6): 13.80 (bs, 1H), 13.53 (bs, 1H), 7.94 (dd, *J* = 5 Hz, 1H), 7.85-7.74 (m, 2H), 7.72-7.69 (m, 1H),

7.63 (bs, 1H), 7.32-7.29 (m, 3H), 7.26-7.21 (m, 2H), 4.06 (s, 2H); HRMS (ESI+) calcd for $C_{17}H_{15}N_4O_3S$ $(M+H)^+$ 355.0859, found 355.0863.

***o*-{[3-(3-Biphenylyl)-5-thioxo-1,4-dihydro-1,2,4-triazol-4-ylamino]carbonyl}benzoic acid - 19:** White powder; Yield: 44%; LC-MS t_R: 1.52 min; *m/z* (ES+) 416.9 $(M+H^+)$; Mp = 180-181 °C; IR (ATR): 3073, 3031, 2921, 1692 cm^{-1}; ^1H NMR (500 MHz, DMSO-d_6): 14.31 (bs, 1H), 13.20 (bs, 1H), 11.96 (bs, 1H), 8.11 (d, *J* = 10 Hz, 1H), 8.08 (bs, 1H), 7.97 (d, *J* = 10 Hz, 1H), 7.89-7.83 (m, 2H), 7.74-7.61 (m, 5H), 7.47 (t, *J* = 7.5 Hz, 2H), 7.39 (t, *J* = 7.54 Hz, 1H); ^{13}C NMR (125 MHz, DMSO-d_6): 168.3, 167.5, 167.3, 150.4, 140.7, 139.2, 134.3, 131.9, 131.3, 131.0, 129.7, 129.6; 129.4, 129.1, 129.0, 127.9, 126.9, 126.6, 125.6, 125.0; HRMS (ESI+) calcd for $C_{22}H_{17}N_4O_3S$ $(M+H)^+$ 417.1016, found 417.1032.

4-[3-(3-Biphenylyl)-5-thioxo-1,4-dihydro-1,2,4-triazol-4-ylamino]-4-oxobutyric acid - 20: White powder; Yield: 31%; LC-MS t_R: 1.38 min; *m/z* (ES+) 369.1 $(M+H^+)$; Mp > 250 °C; ^1H NMR (500 MHz, DMSO-d_6): 14.19 (bs, 1H), 12.21 (bs, 1H), 11.60 (bs, 1H), 7.97 (bs, 1H), 7.86 (d, *J* = 5 Hz, 1H), 7.72-7.68 (m, 3H), 7.61 (t, *J* = 7.5 Hz, 1H), 7.50 (t, *J* = 7.5 Hz, 2H), 7.42 (t, *J* = 7.5 Hz, 1H), 2.61-2.56 (m, 2H), 2.51-2.45 (m, 2H); ^{13}C NMR (125 MHz, DMSO-d_6): 173.2, 171.0, 167.9, 150.1, 140.7, 139.0, 129.7, 129.2, 129.1, 128.0, 126.8, 126.2, 124.4, 125.2, 39.4, 28.4; HRMS (ESI+) calcd for $C_{18}H_{17}NO_3S$ $(M+H)^+$ 369.1016, found 369.1029.

A **B**

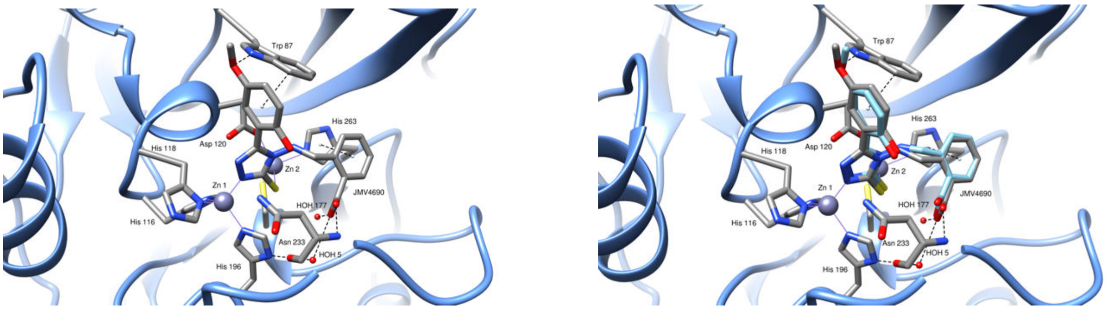

Figure S1. (A) View of the crystallographic structure of the complex between VIM-2 and JMV4690 used for docking experiments (6YRP.pdb, [Gavara L. et al. unpublished, 2020]). (B) Docking of JMV4690 (light blue) in VIM-2 using AutoDock 4.2. The docked pose of JMV4690 was superimposed with the experimental pose.

Table S1. Docking data for compounds JMV4690, JMV4390, **1** and **17**.[a]

Cpd	Exp. K_i (μM)	Best free energy (kcal/mol)	Estimated K_i (μM)	Percentage of docking solutions[c]	Distance N^2 – Zn1 (Å)	Distance S – Zn2 (Å)
JMV4690	0.7[b]	-8.54	0.55	13	1.96	2.27
JMV4390	2.7[b]	-7.54	2.98	6	2.19	1.91
**1**	1.4	-7.03	7.01	8	2.12	1.90
**17**	NI	-6.64	13.5	15	2.44	2.13

[a]Docking data were obtained using AutoDock 4.2. [b]From Gavara 2020. [c]Docking was performed on VIM-2 devoid of the hydroxide anion between the two zincs letting two positive charges without screening. For this reason, about 90% of solutions placed the carboxylate between the two zincs.

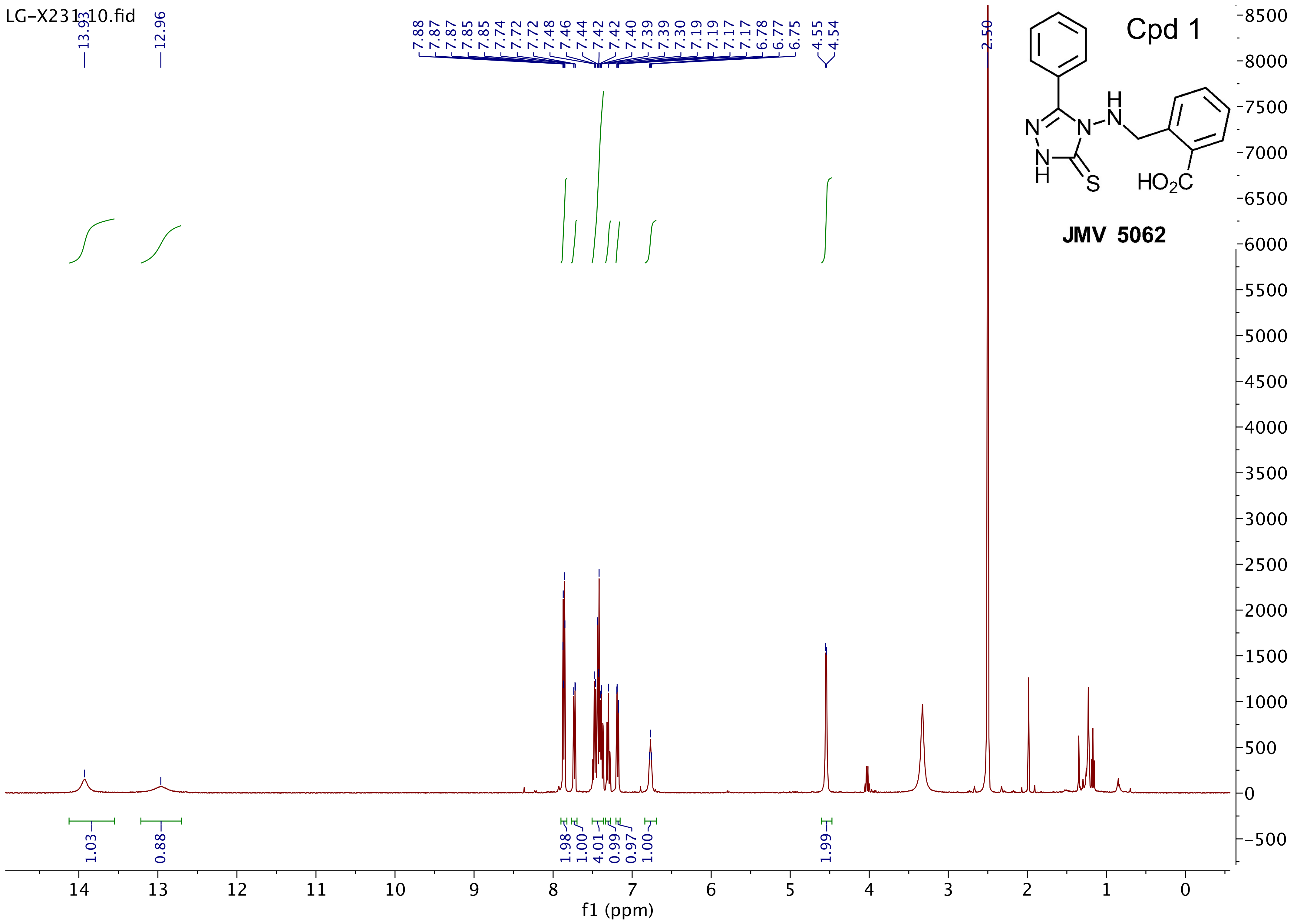

LG-X231-10.fid
Cpd 1
JMV 5062
HO2C
13.93
12.96
7.88 7.87 7.87 7.85 7.85 7.74 7.72 7.72 7.48 7.46 7.44 7.42 7.42 7.40 7.39 7.39 7.30 7.19 7.19 7.17 7.17 6.78 6.77 6.75
4.55 4.54
2.50
1.03
0.88
1.98
1.00
4.01
0.99
0.97
1.00
1.99
f1 (ppm)

LG-X231.11.fid
LG-X231 / dmso

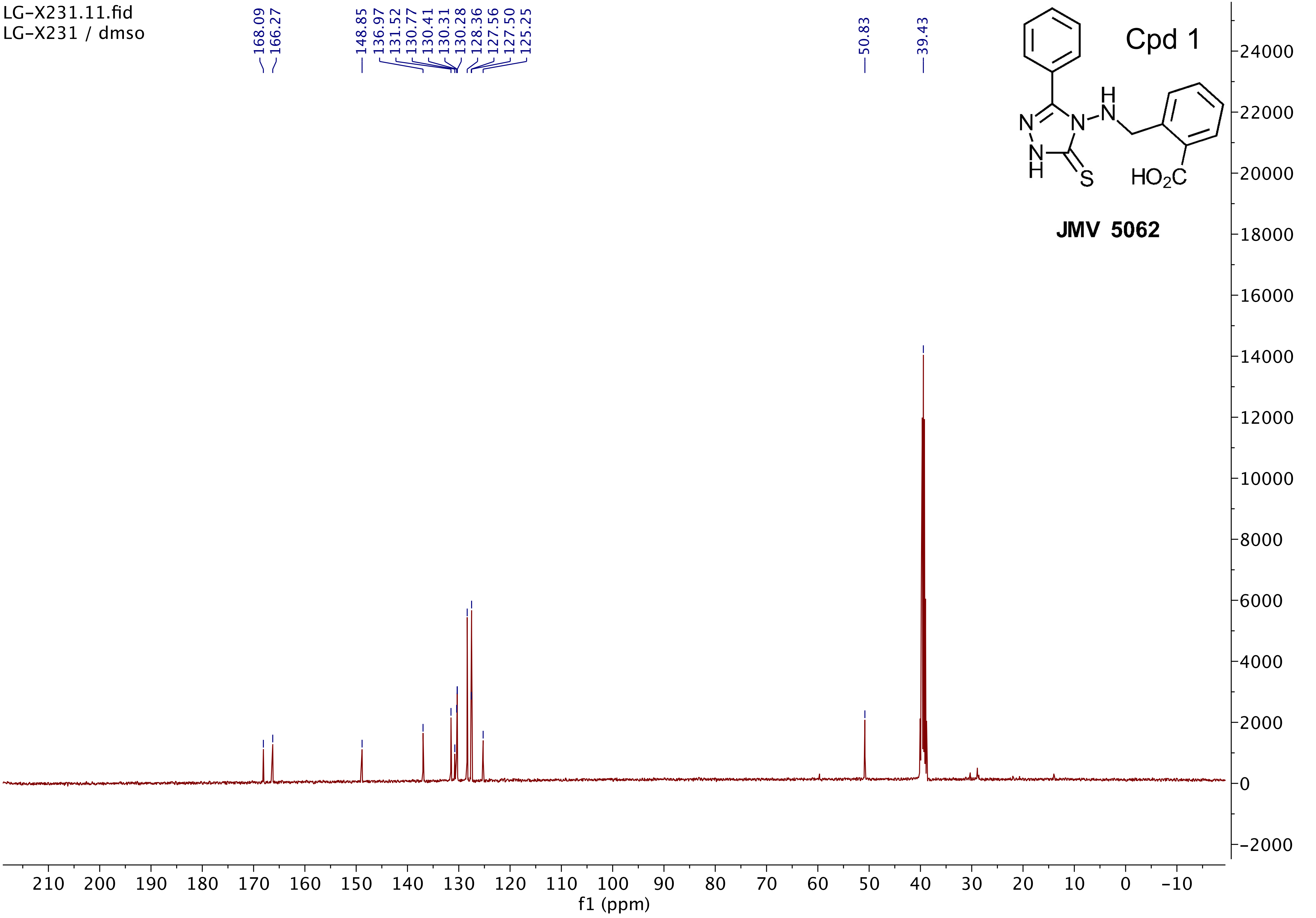

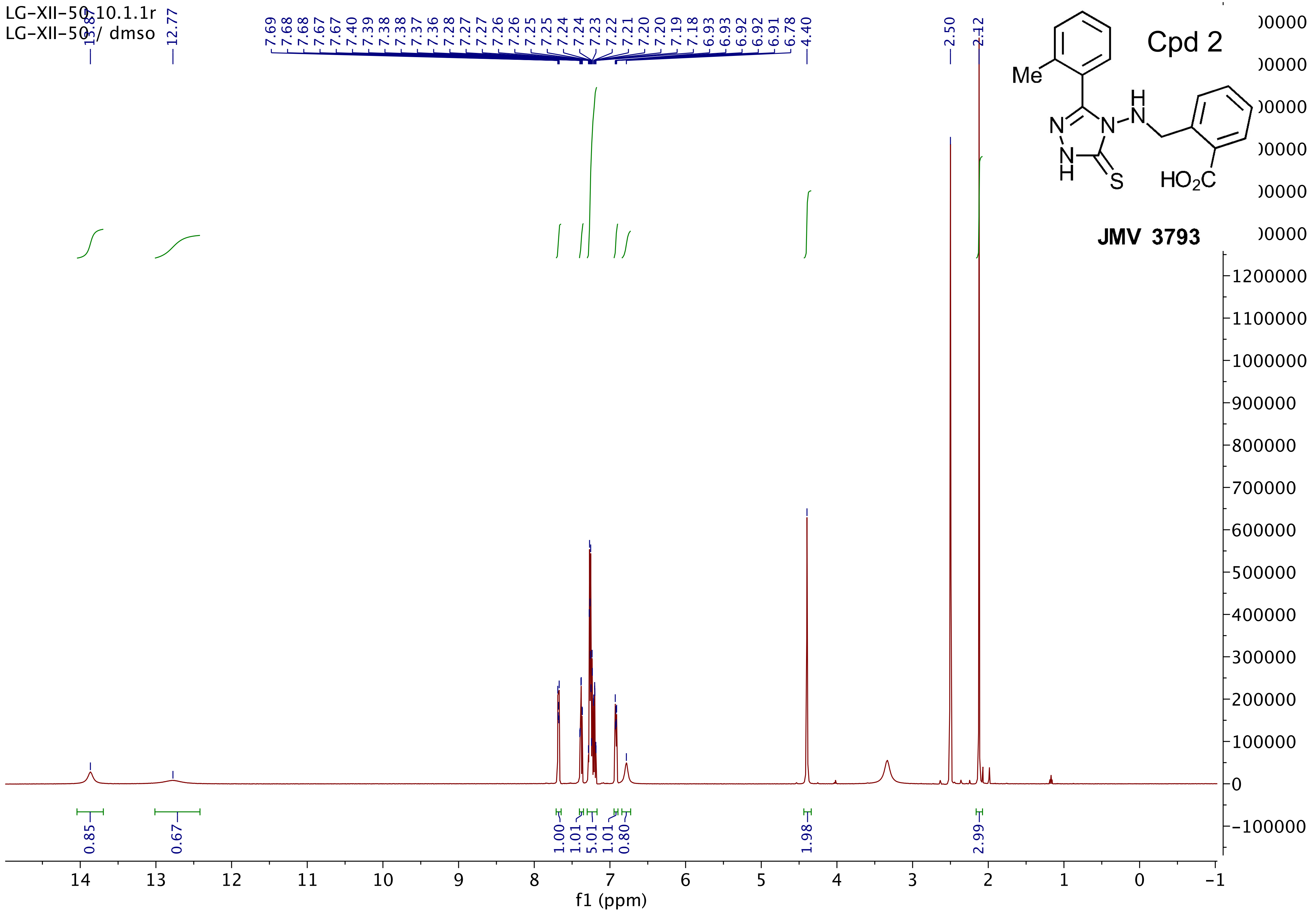

LG-XII-50.10.1.1r
LG-XII-50 / dmso
13.87
12.77
7.69
7.68
7.68
7.67
7.67
7.40
7.39
7.38
7.38
7.37
7.36
7.28
7.27
7.27
7.26
7.26
7.25
7.25
7.24
7.24
7.23
7.22
7.21
7.20
7.20
7.19
7.18
6.93
6.93
6.92
6.92
6.91
6.78
4.40
2.50
2.12
Cpd 2
Me
N
N
H
N
H
N
S
HO2C
JMV 3793
0.85
0.67
1.00
1.01
5.01
1.01
0.80
1.98
2.99
f1 (ppm)

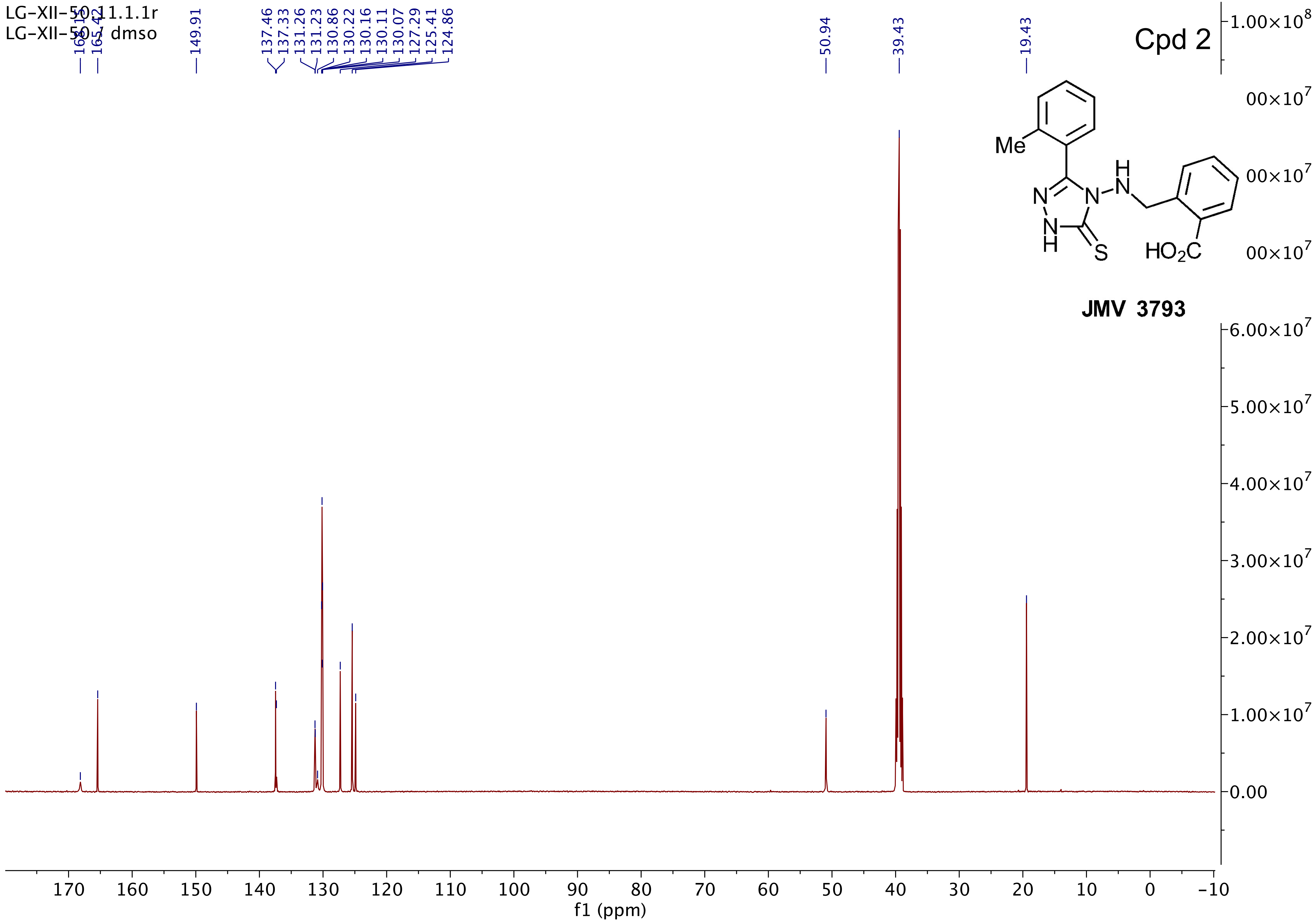

LG-XII-50.11.1.1r
LG-XII-50.7 dmso
165.42
149.91
137.46
137.33
131.26
131.23
130.86
130.22
130.16
130.11
130.07
127.29
125.41
124.86
50.94
39.43
19.43
Cpd 2
Me
N
N
H
N
H
S
HO2C
JMV 3793
1.00×10^8
6.00×10^7
5.00×10^7
4.00×10^7
3.00×10^7
2.00×10^7
1.00×10^7
0.00
170
160
150
140
130
120
110
100
90
80
70
60
50
40
30
20
10
0
−10
f1 (ppm)

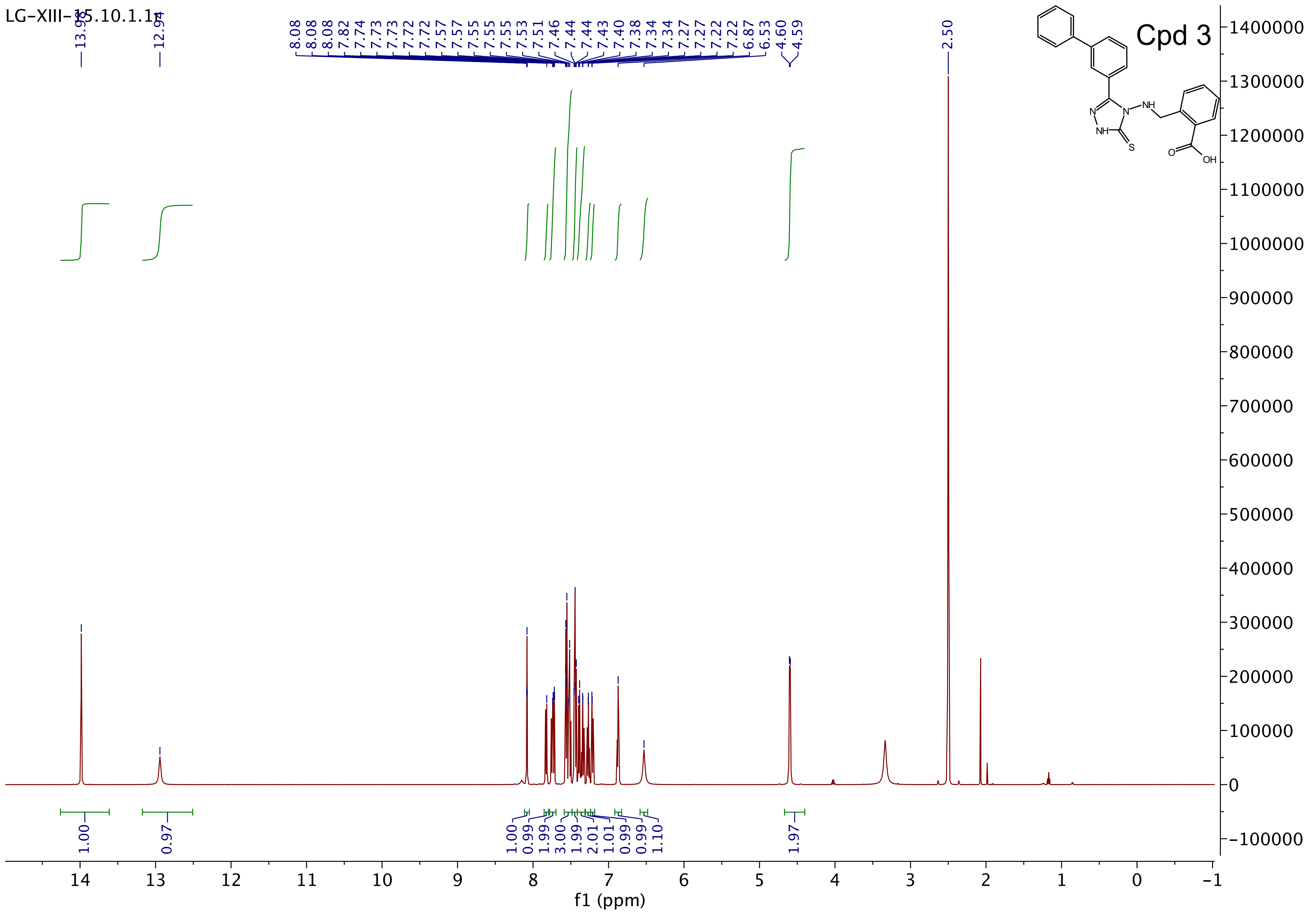

LG-XIII-15.10.1.1r
Cpd 3
13.98
12.94
8.08
8.08
8.08
7.82
7.74
7.73
7.73
7.72
7.72
7.57
7.57
7.55
7.55
7.55
7.53
7.51
7.46
7.44
7.44
7.43
7.40
7.38
7.34
7.34
7.27
7.27
7.22
7.22
6.87
6.53
4.60
4.59
2.50
1.00
0.97
1.00
0.99
1.99
3.00
1.99
2.01
1.01
0.99
0.99
1.10
1.97
f1 (ppm)

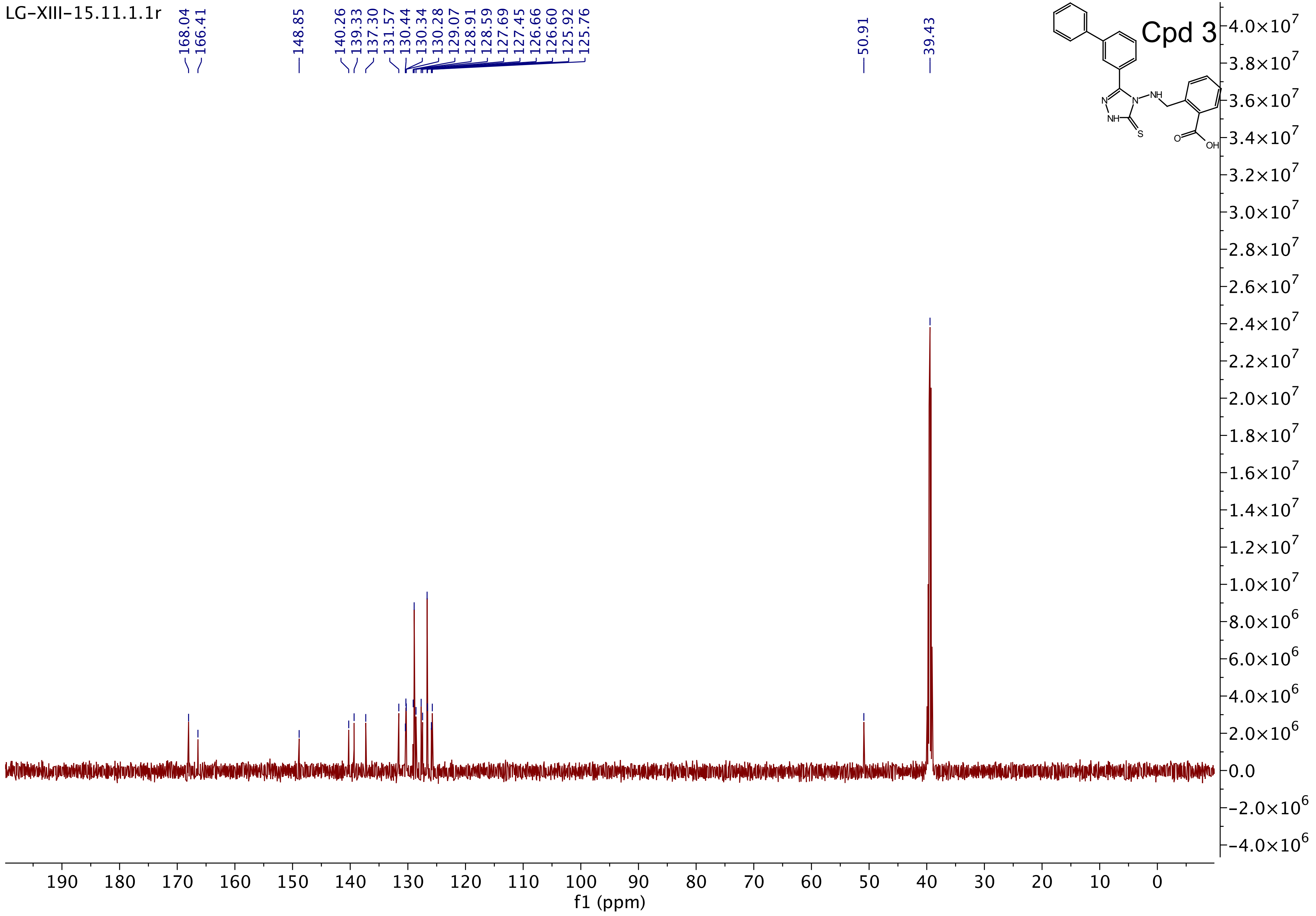

LG-XIII-15.11.1.1r
168.04
166.41
148.85
140.26
139.33
137.30
131.57
130.44
130.34
130.28
129.07
128.91
128.59
127.69
127.45
126.66
126.60
125.92
125.76
50.91
39.43
Cpd 3
N
NH
N
NH
S
O
OH
4.0×10^7
3.8×10^7
3.6×10^7
3.4×10^7
3.2×10^7
3.0×10^7
2.8×10^7
2.6×10^7
2.4×10^7
2.2×10^7
2.0×10^7
1.8×10^7
1.6×10^7
1.4×10^7
1.2×10^7
1.0×10^7
8.0×10^6
6.0×10^6
4.0×10^6
2.0×10^6
0.0
-2.0×10^6
-4.0×10^6
190 180 170 160 150 140 130 120 110 100 90 80 70 60 50 40 30 20 10 0
f1 (ppm)

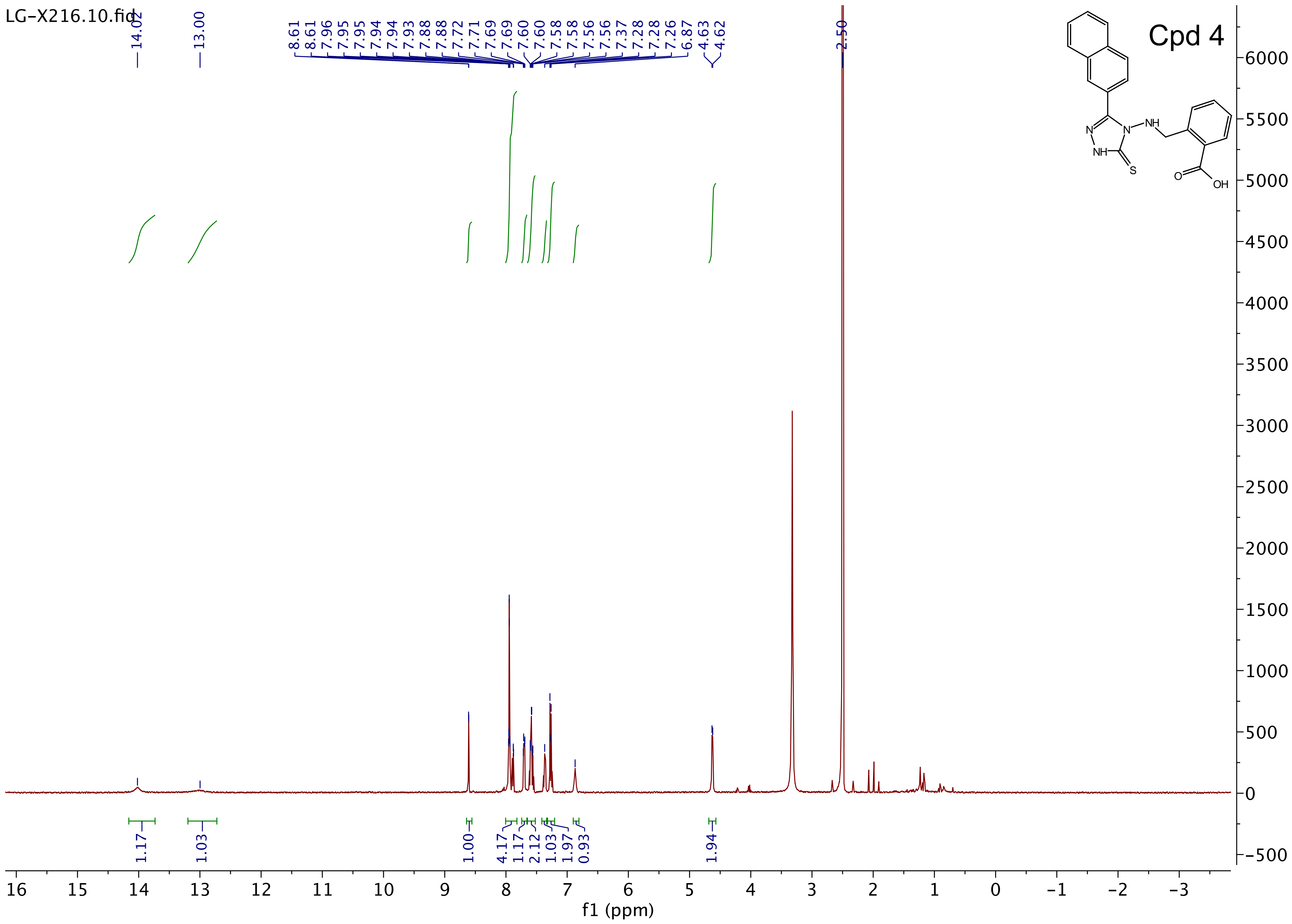
LG-X216.10.fid
Cpd 4
14.02
13.00
8.61 8.61 7.96 7.95 7.95 7.94 7.94 7.93 7.88 7.88 7.72 7.71 7.69 7.69 7.60 7.60 7.58 7.58 7.56 7.56 7.37 7.28 7.28 7.26 6.87 4.63 4.62
2.50
N
NH
N
NH
S
O
OH
1.17
1.03
1.00
4.17
1.17
2.12
1.03
1.97
0.93
1.94
16 15 14 13 12 11 10 9 8 7 6 5 4 3 2 1 0 −1 −2 −3
f1 (ppm)
6000 5500 5000 4500 4000 3500 3000 2500 2000 1500 1000 500 0 −500

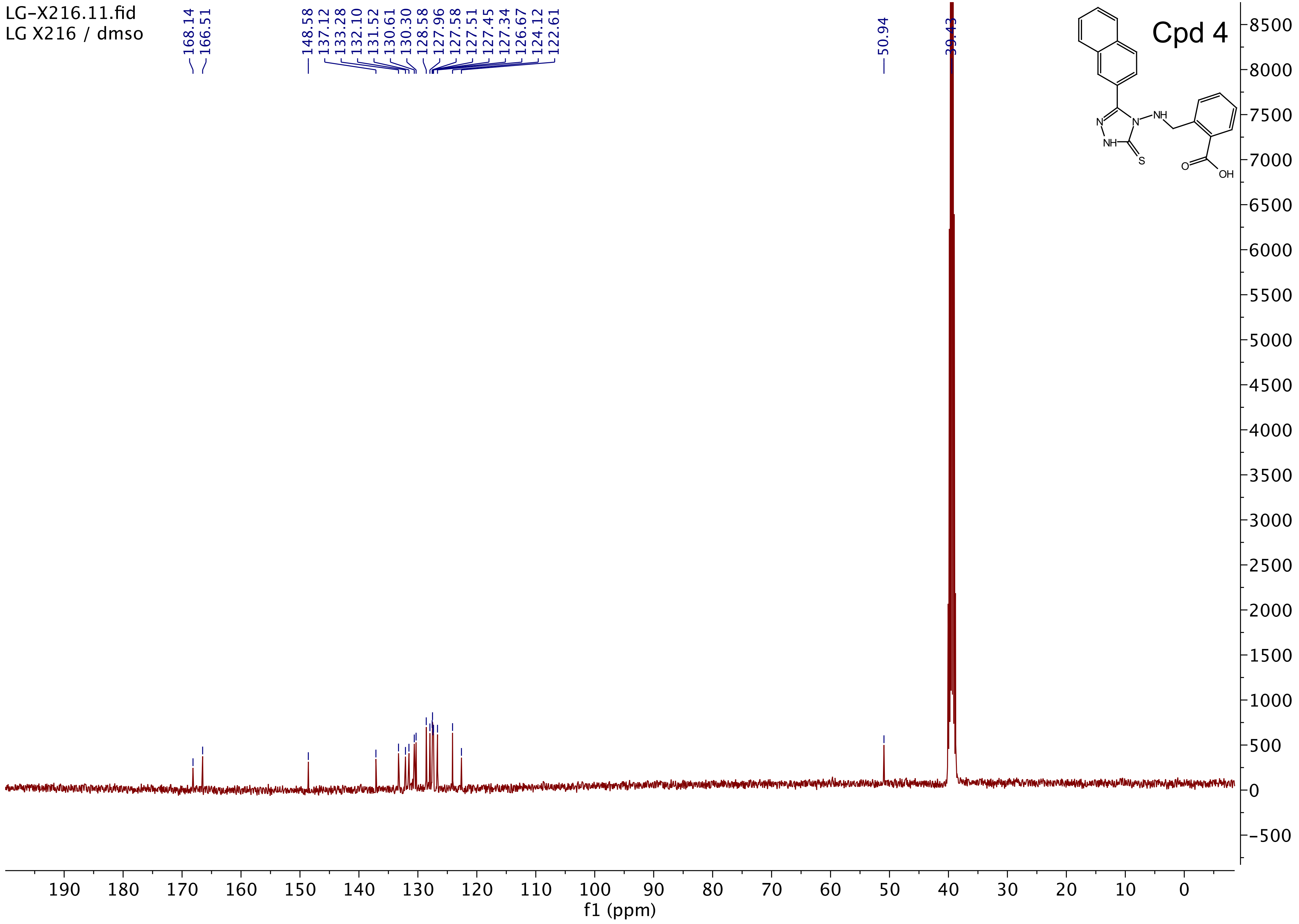

LG-X216.11.fid
LG X216 / dmso
168.14
166.51
148.58
137.12
133.28
132.10
131.52
130.61
130.30
128.58
127.96
127.58
127.51
127.45
127.34
126.67
124.12
122.61
50.94
39.43
Cpd 4
NH
N
NH
S
O
OH
f1 (ppm)

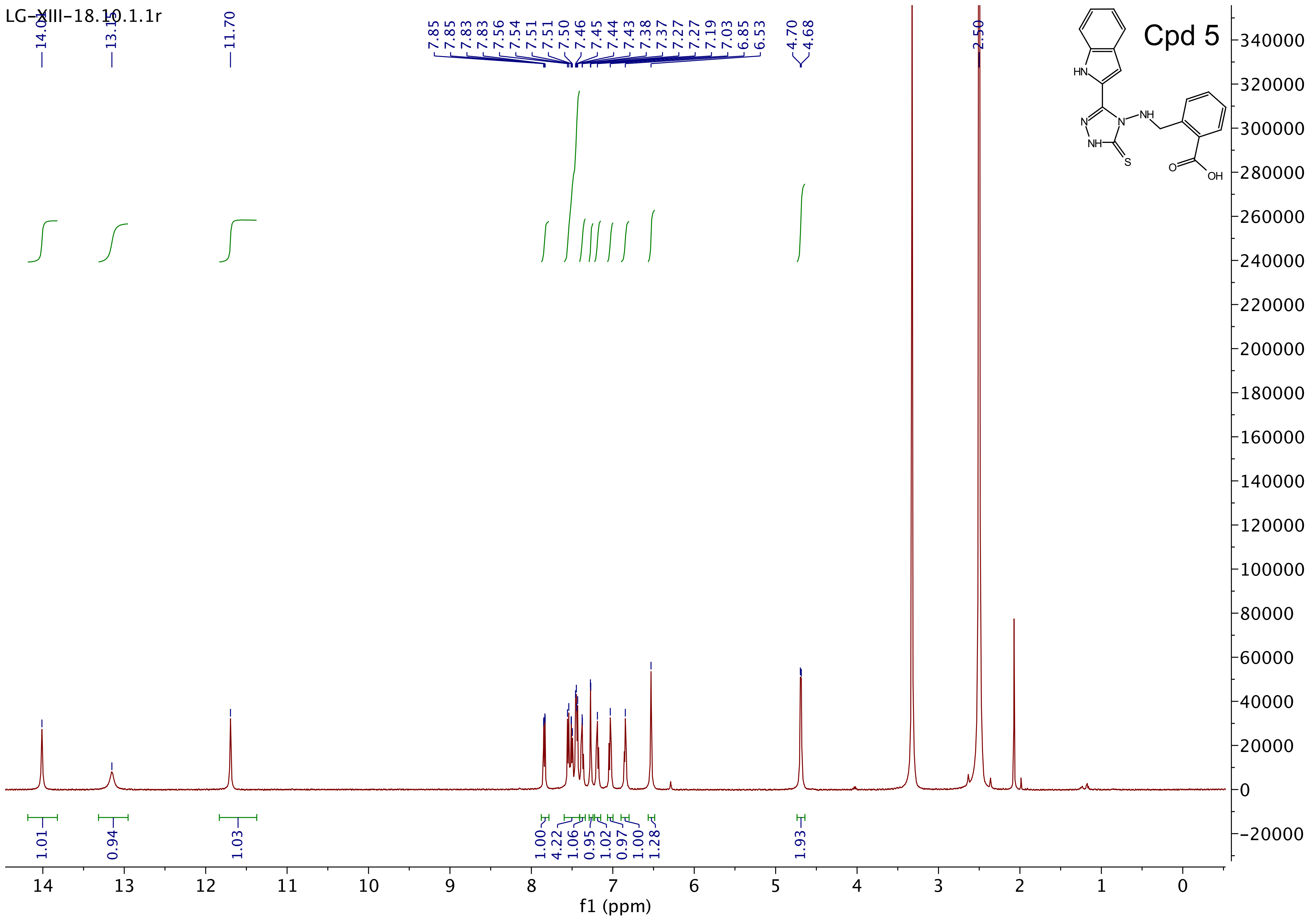

LG-XIII-18.10.1.1r
Cpd 5
14.01
13.15
11.70
7.85 7.85 7.83 7.83 7.56 7.54 7.51 7.51 7.50 7.46 7.45 7.44 7.43 7.38 7.37 7.27 7.27 7.19 7.03 6.85 6.53
4.70 4.68
2.50
HN
N
NH
N
NH
S
O
OH
1.01 0.94 1.03 1.00 4.22 1.06 0.95 1.02 0.97 1.00 1.28 1.93
f1 (ppm)

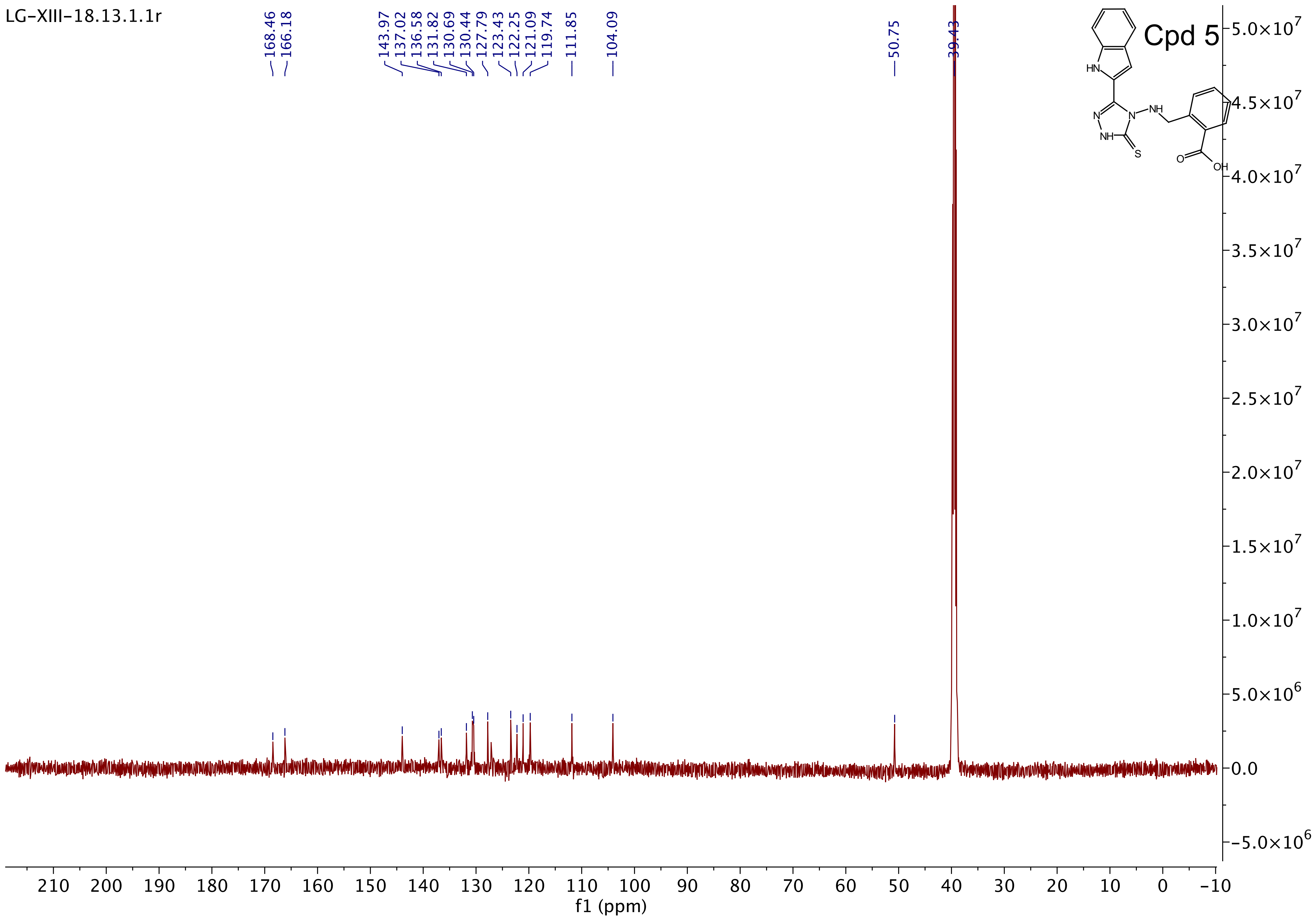

LG-XIII-18.13.1.1r
168.46
166.18
143.97
137.02
136.58
131.82
130.69
130.44
127.79
123.43
122.25
121.09
119.74
111.85
104.09
50.75
39.43
Cpd 5
HN
N
N
NH
S
NH
O
OH
5.0×10^7
4.5×10^7
4.0×10^7
3.5×10^7
3.0×10^7
2.5×10^7
2.0×10^7
1.5×10^7
1.0×10^7
5.0×10^6
0.0
−5.0×10^6
210 200 190 180 170 160 150 140 130 120 110 100 90 80 70 60 50 40 30 20 10 0 −10
f1 (ppm)

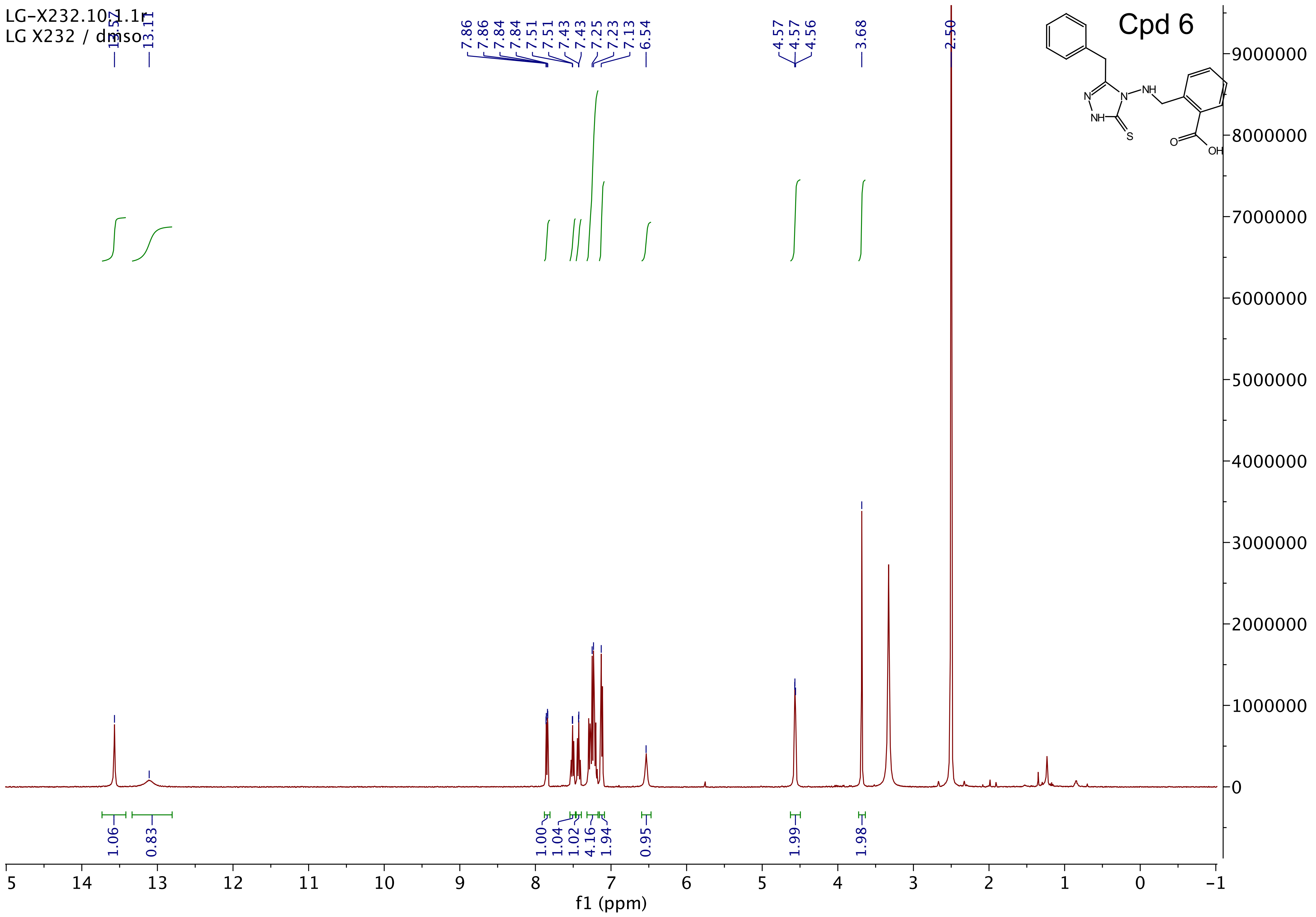

LG-X232.10.1.1r
LG X232 / dmso
13.57
13.11
7.86
7.86
7.84
7.84
7.51
7.51
7.43
7.43
7.25
7.23
7.13
6.54
4.57
4.57
4.56
3.68
2.50
Cpd 6
N
NH
N
NH
S
O
OH
9000000
8000000
7000000
6000000
5000000
4000000
3000000
2000000
1000000
0
1.06
0.83
1.00
1.04
1.02
4.16
1.94
0.95
1.99
1.98
5
14
13
12
11
10
9
8
7
6
5
4
3
2
1
0
–1
f1 (ppm)

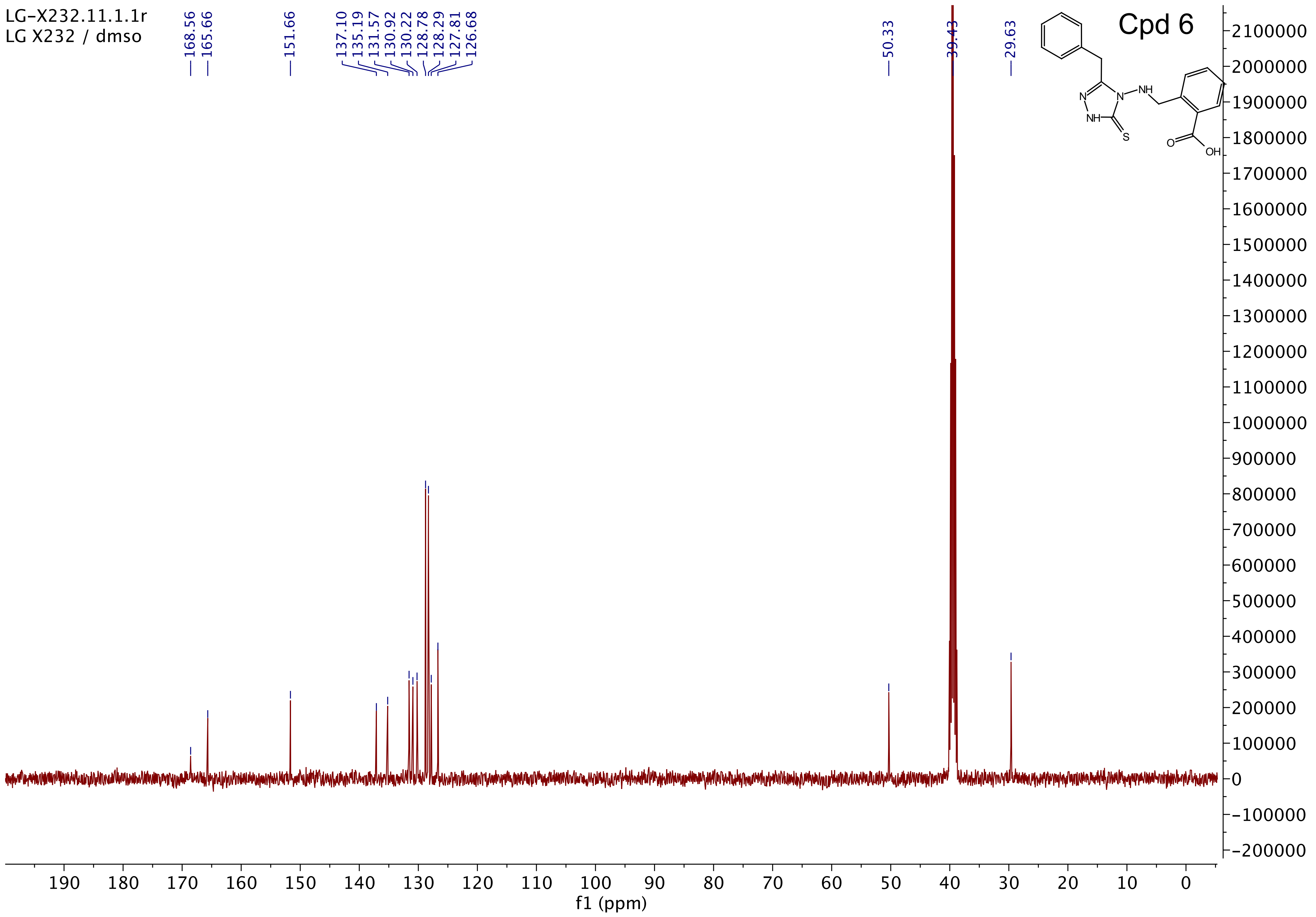

LG-X232.11.1.1r
LG X232 / dmso
168.56
165.66
151.66
137.10
135.19
131.57
130.92
130.22
128.78
128.29
127.81
126.68
50.33
39.43
29.63
Cpd 6
N
NH
N
NH
S
O
OH
f1 (ppm)

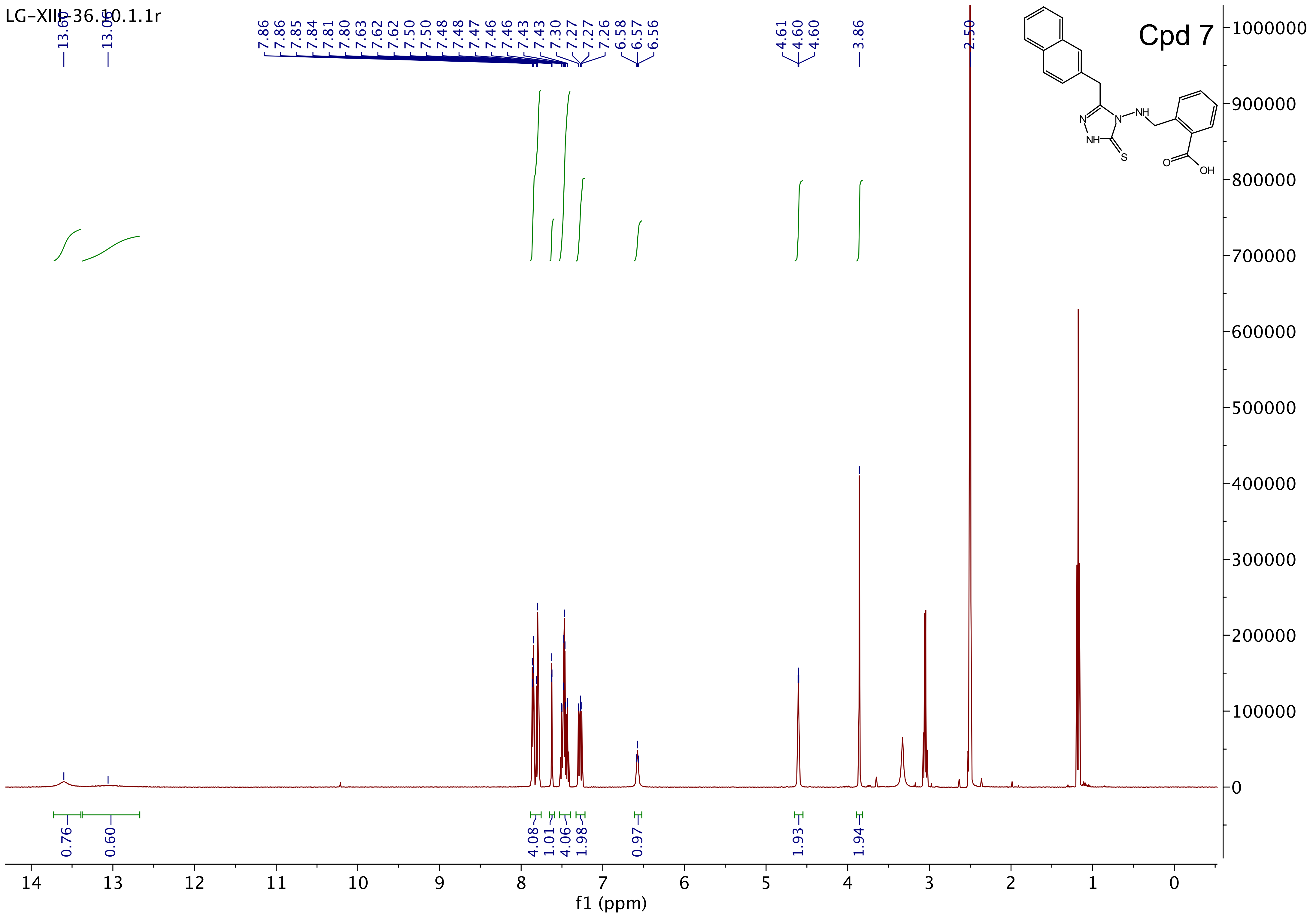
LG-XIII-36.10.1.1r
Cpd 7
13.60
13.08
7.86
7.86
7.85
7.84
7.81
7.80
7.63
7.62
7.62
7.50
7.50
7.48
7.48
7.47
7.46
7.46
7.43
7.43
7.30
7.27
7.27
7.26
6.58
6.57
6.56
4.61
4.60
4.60
3.86
2.50
0.76
0.60
4.08
1.01
4.06
1.98
0.97
1.93
1.94
f1 (ppm)

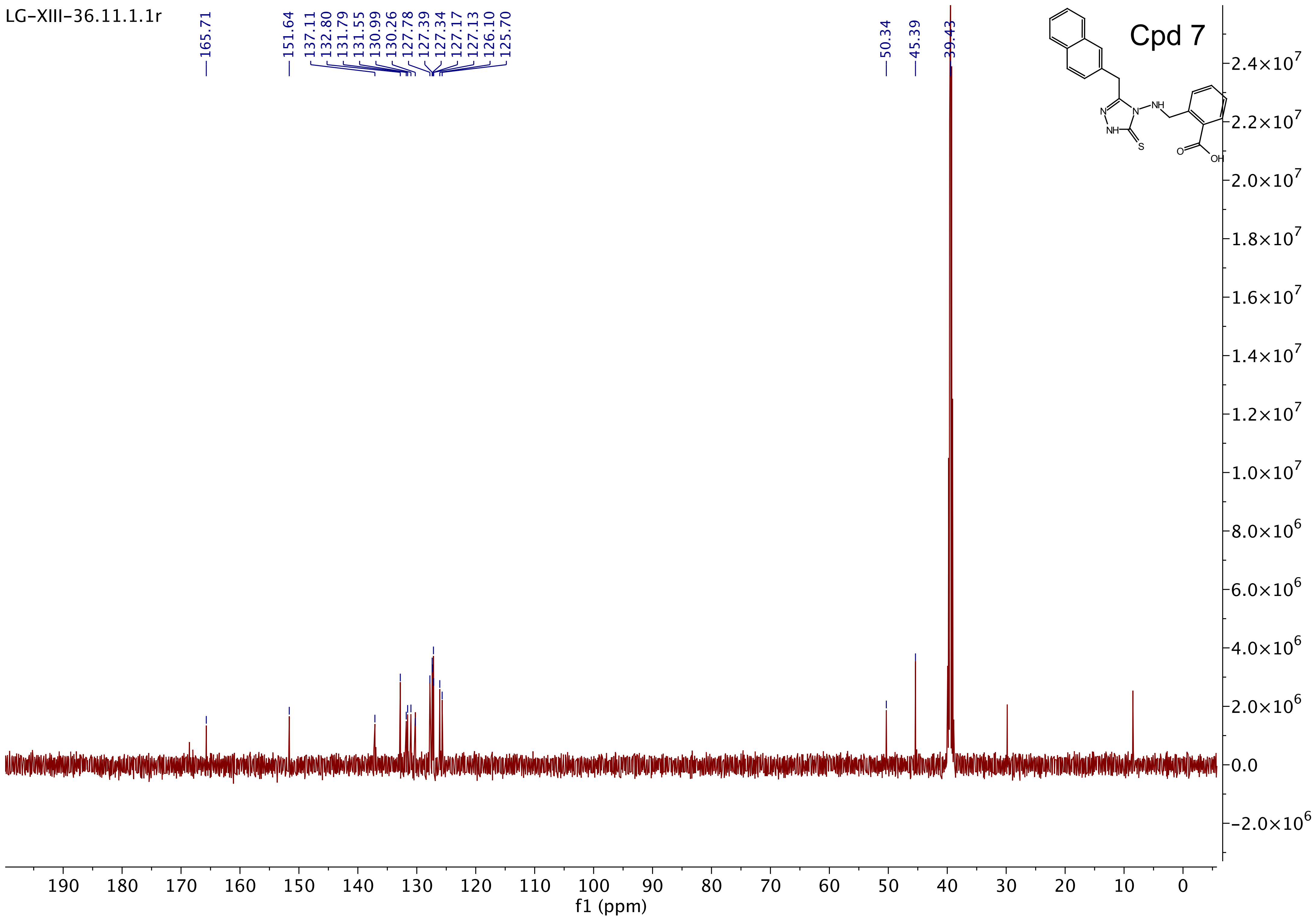
LG-XIII-36.11.1.1r
165.71
151.64
137.11
132.80
131.79
131.55
130.99
130.26
127.78
127.39
127.34
127.17
127.13
126.10
125.70
50.34
45.39
39.43
Cpd 7
N
NH
N
NH
S
O
OH
2.4×10^7
2.2×10^7
2.0×10^7
1.8×10^7
1.6×10^7
1.4×10^7
1.2×10^7
1.0×10^7
8.0×10^6
6.0×10^6
4.0×10^6
2.0×10^6
0.0
−2.0×10^6
190 180 170 160 150 140 130 120 110 100 90 80 70 60 50 40 30 20 10 0
f1 (ppm)

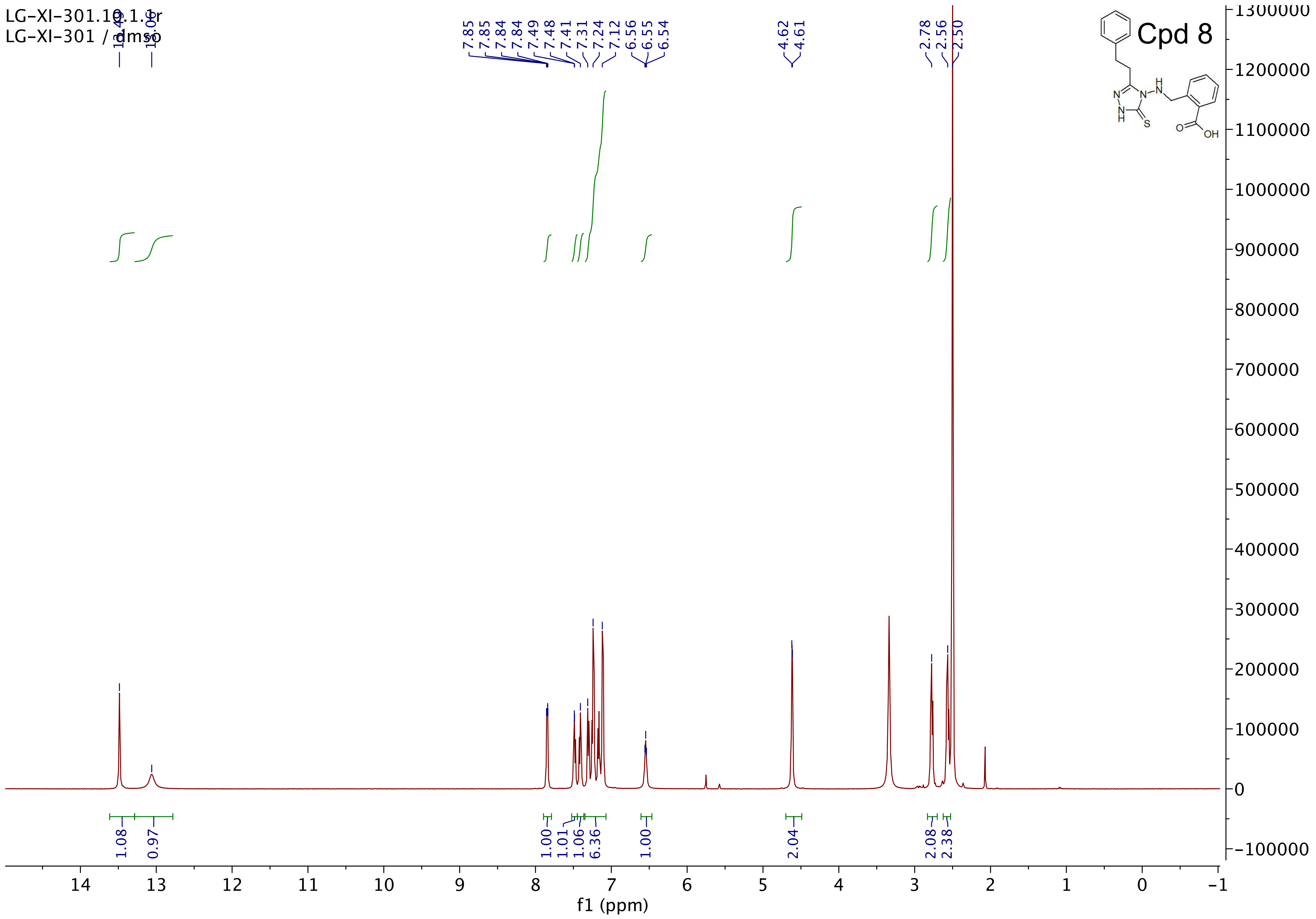
LG–XI–301 / dmso
Cpd 8
7.85 7.85 7.84 7.84 7.49 7.48 7.41 7.31 7.24 7.12 6.56 6.55 6.54
4.62 4.61
2.78 2.56 2.50
1.08 0.97 1.00 1.01 1.06 6.36 1.00 2.04 2.08 2.38
f1 (ppm)

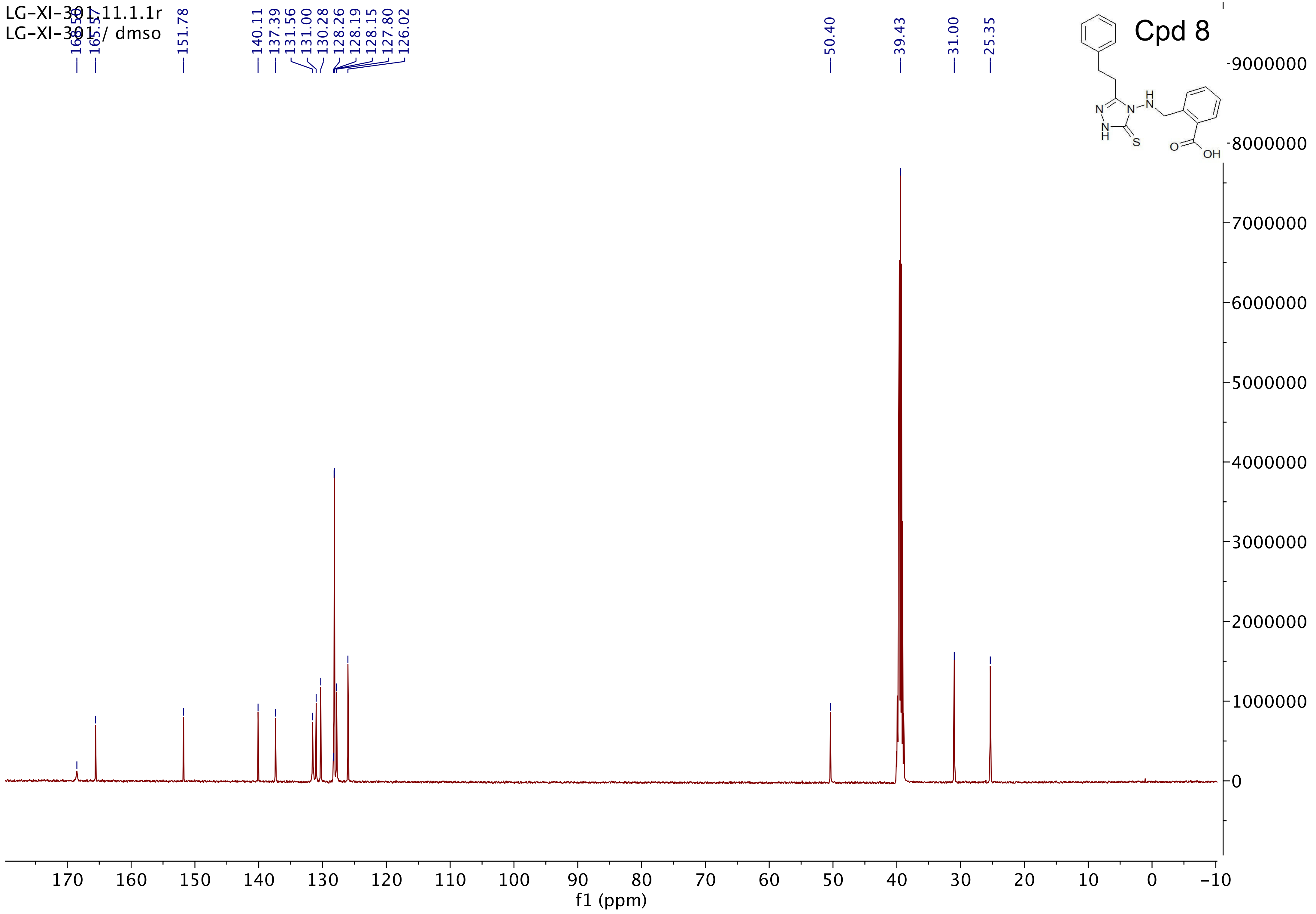

LG-XI-301.11.1.1r
LG-XI-301 / dmso
168.50
165.57
151.78
140.11
137.39
131.56
131.00
130.28
128.26
128.19
128.15
127.80
126.02
50.40
39.43
31.00
25.35
Cpd 8
N
N
H
N
H
N
S
O
OH
9000000
8000000
7000000
6000000
5000000
4000000
3000000
2000000
1000000
0
170
160
150
140
130
120
110
100
90
80
70
60
50
40
30
20
10
0
–10
f1 (ppm)

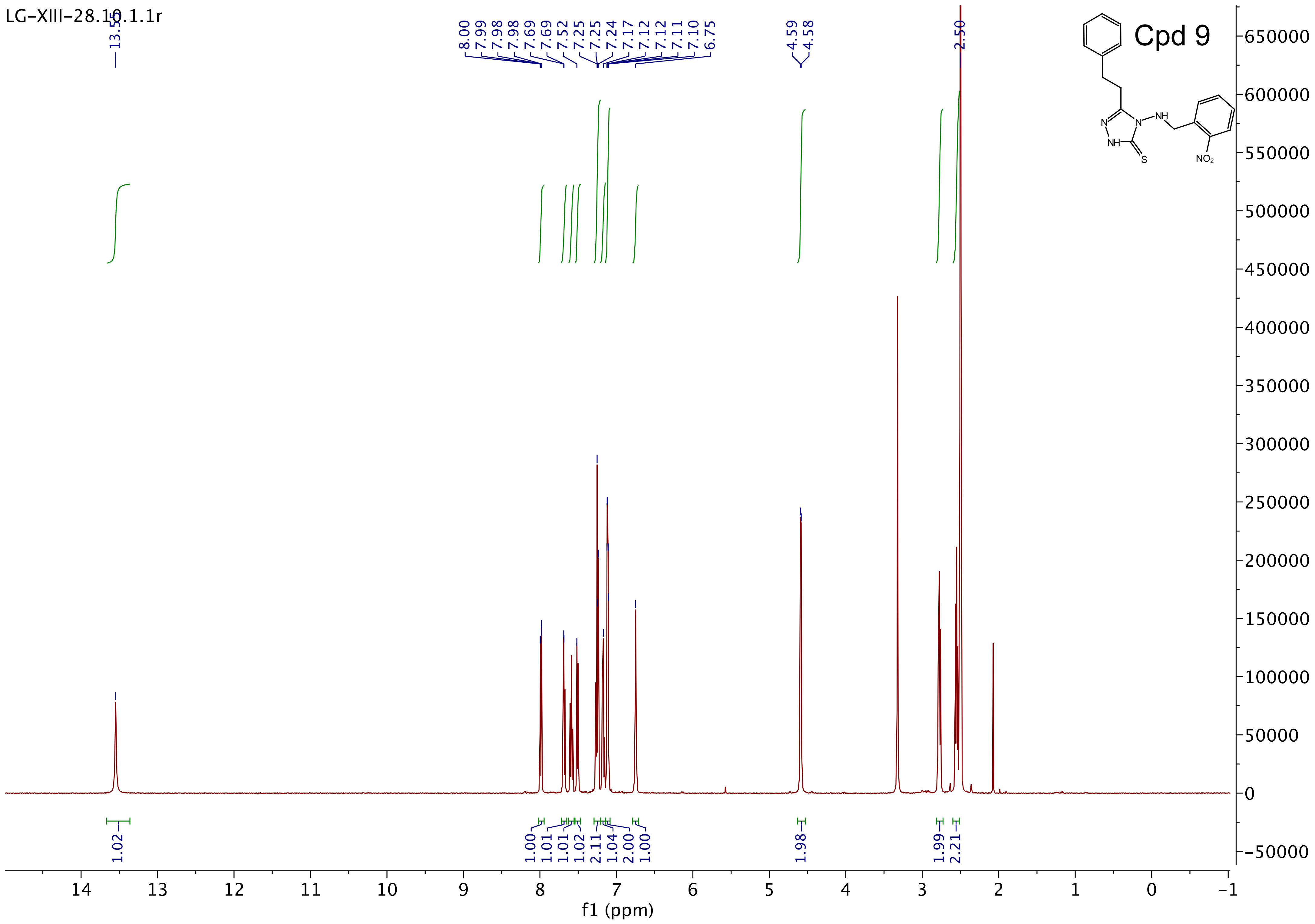
LG-XIII-28.10.1.1r
Cpd 9
13.55
8.00
7.99
7.98
7.98
7.69
7.69
7.52
7.25
7.25
7.24
7.17
7.12
7.12
7.11
7.10
6.75
4.59
4.58
2.50
1.02
1.00
1.01
1.01
1.02
2.11
1.04
2.00
1.00
1.98
1.99
2.21
f1 (ppm)
NH
N
NH
S
NO2

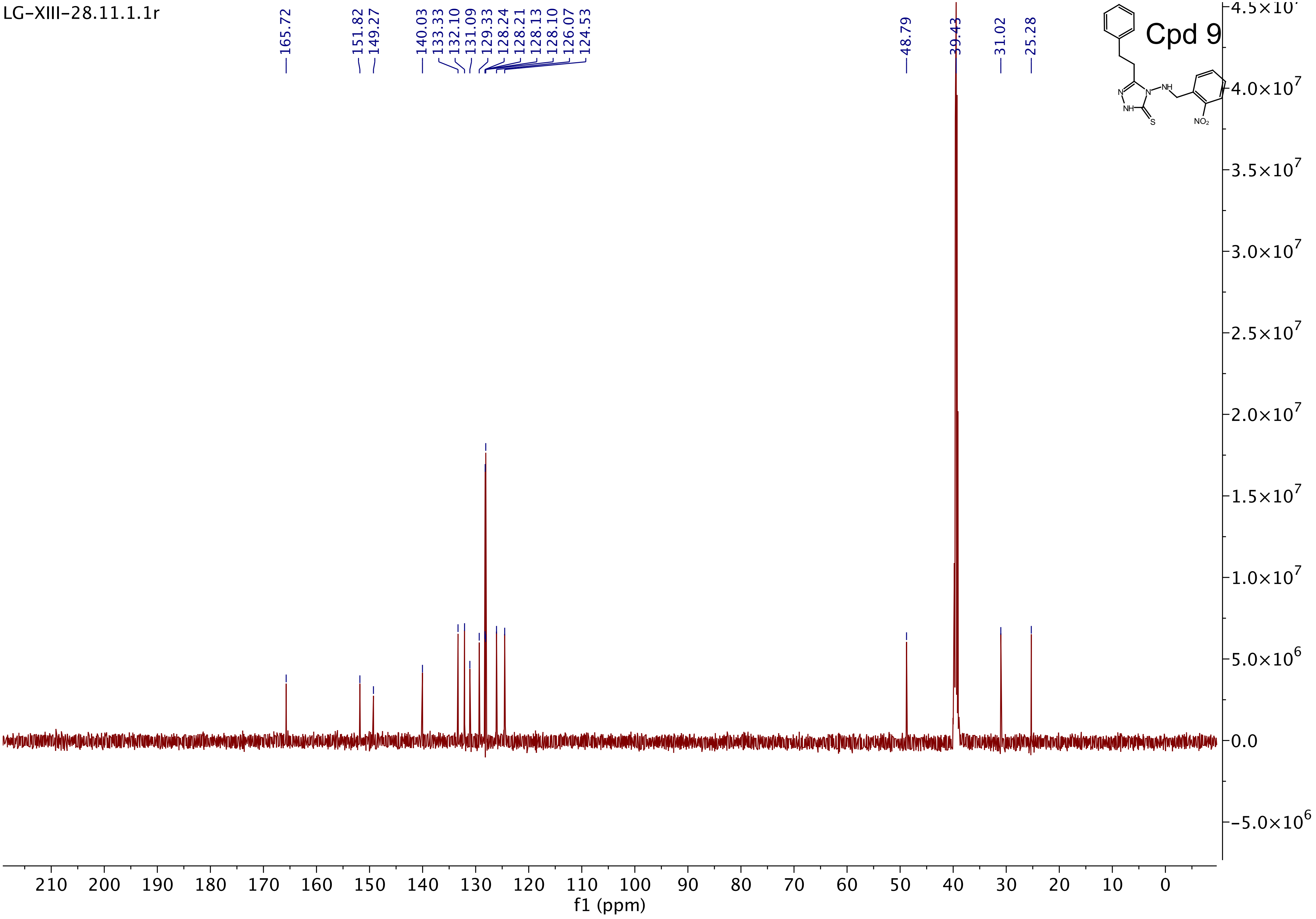
LG-XIII-28.11.1.1r
165.72
151.82
149.27
140.03
133.33
132.10
131.09
129.33
128.24
128.21
128.13
128.10
126.07
124.53
48.79
39.43
31.02
25.28
Cpd 9
NH
N
N
NH
S
NO2
f1 (ppm)

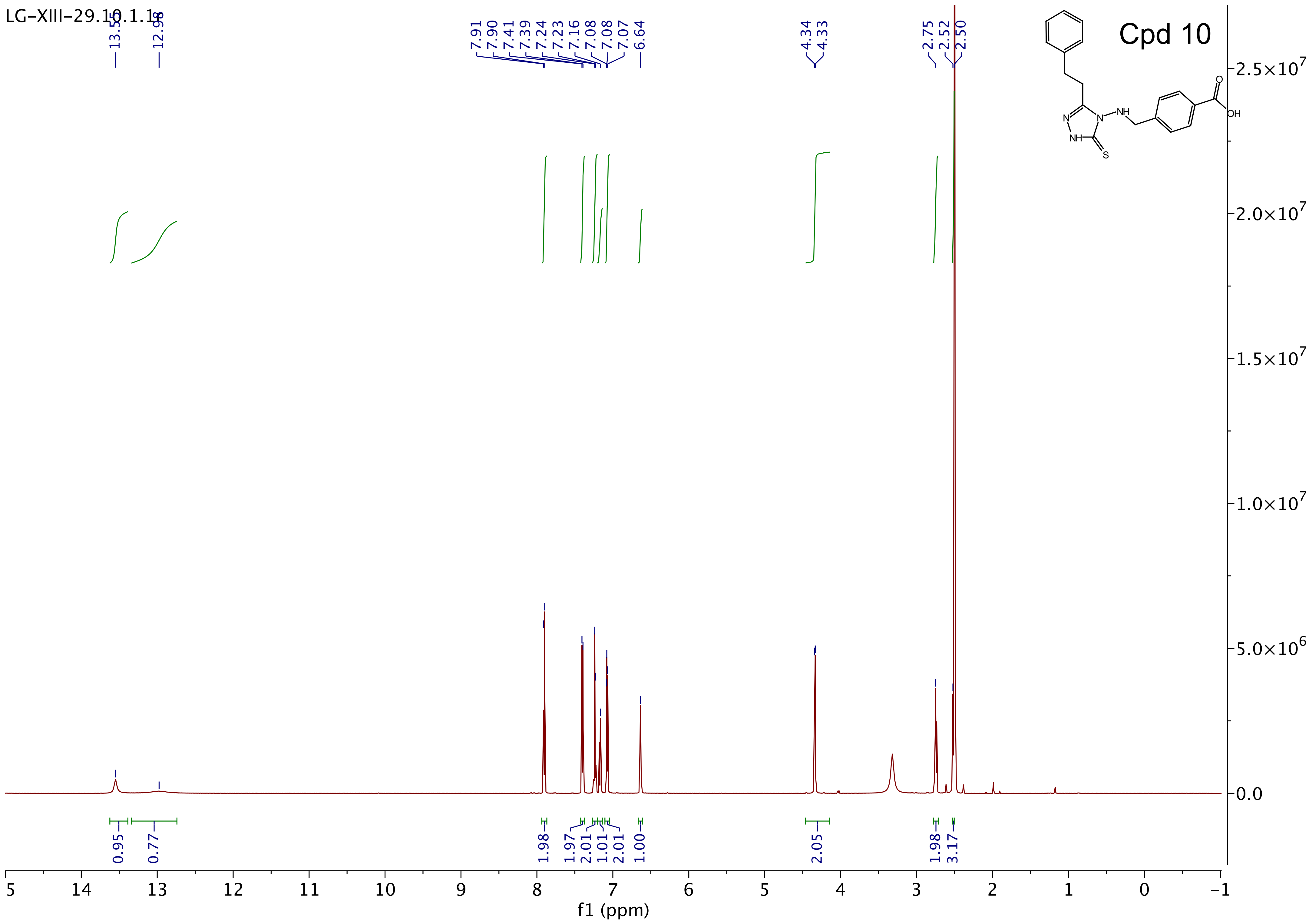

LG-XIII-29.10.1.1
Cpd 10
13.55
12.98
7.91
7.90
7.41
7.39
7.24
7.23
7.16
7.08
7.08
7.07
6.64
4.34
4.33
2.75
2.52
2.50
0.95
0.77
1.98
1.97
2.01
1.01
2.01
1.00
2.05
1.98
3.17
NH
N
N
NH
S
O
OH
f1 (ppm)

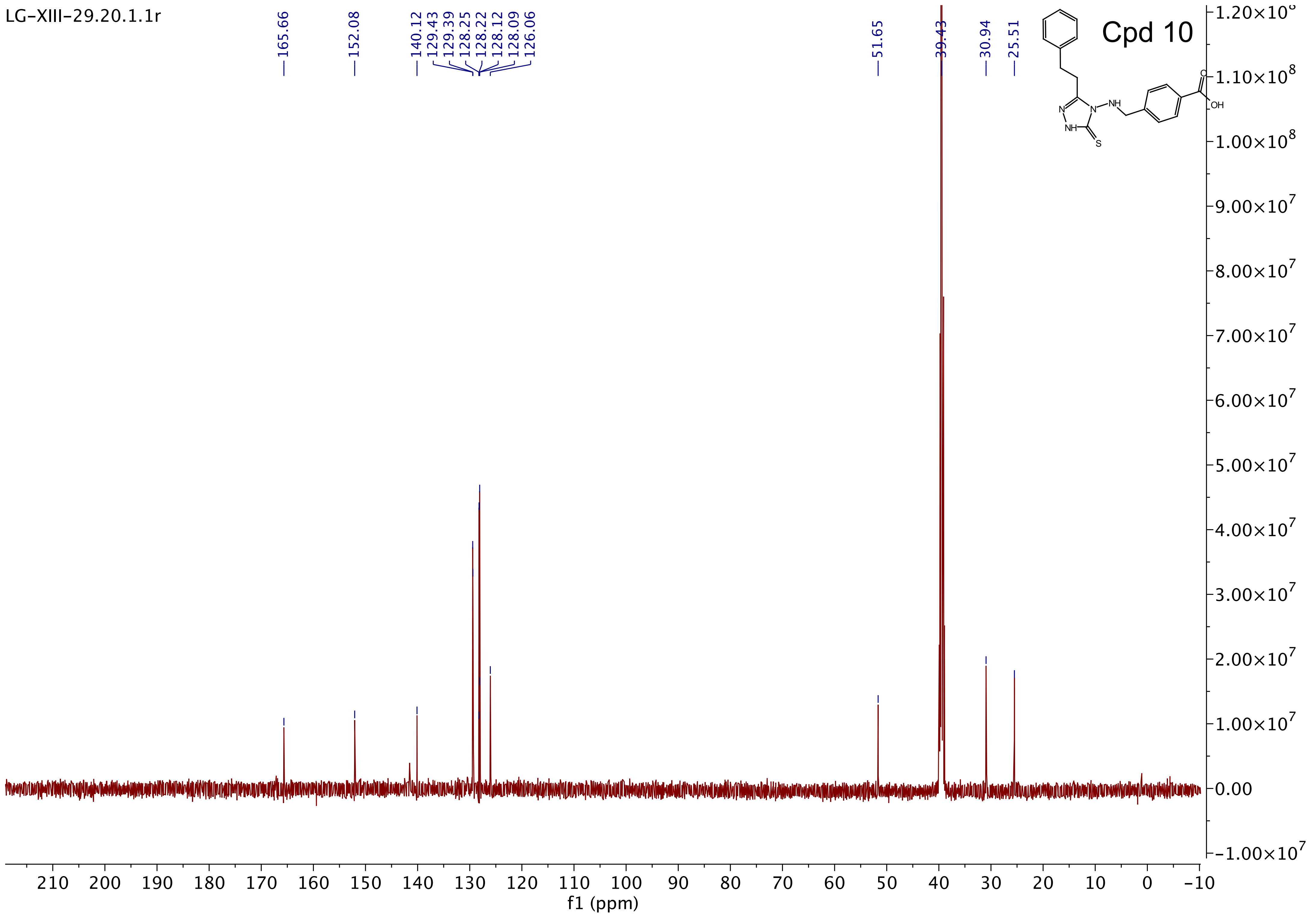
LG-XIII-29.20.1.1r
Cpd 10
165.66
152.08
140.12
129.43
129.39
128.25
128.22
128.12
128.09
126.06
51.65
39.43
30.94
25.51
N
NH
N
NH
S
O
OH
f1 (ppm)

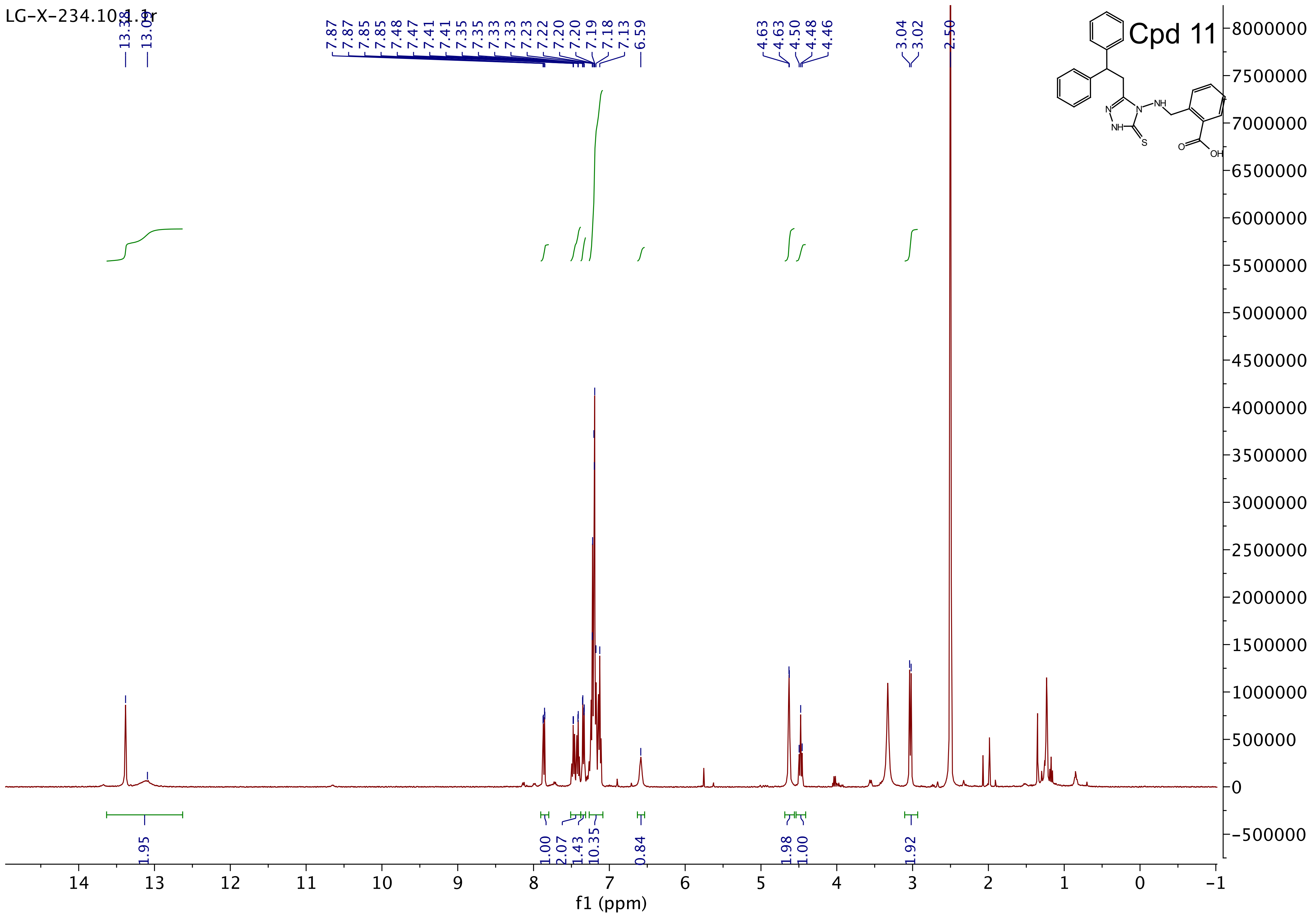

LG-X-234.10.1.1r
Cpd 11
13.38
13.09
7.87
7.87
7.85
7.85
7.48
7.47
7.41
7.41
7.35
7.35
7.33
7.33
7.23
7.22
7.20
7.20
7.19
7.18
7.13
6.59
4.63
4.63
4.50
4.48
4.46
3.04
3.02
2.50
N
NH
N
NH
S
O
OH
1.95
1.00
2.07
1.43
10.35
0.84
1.98
1.00
1.92
f1 (ppm)

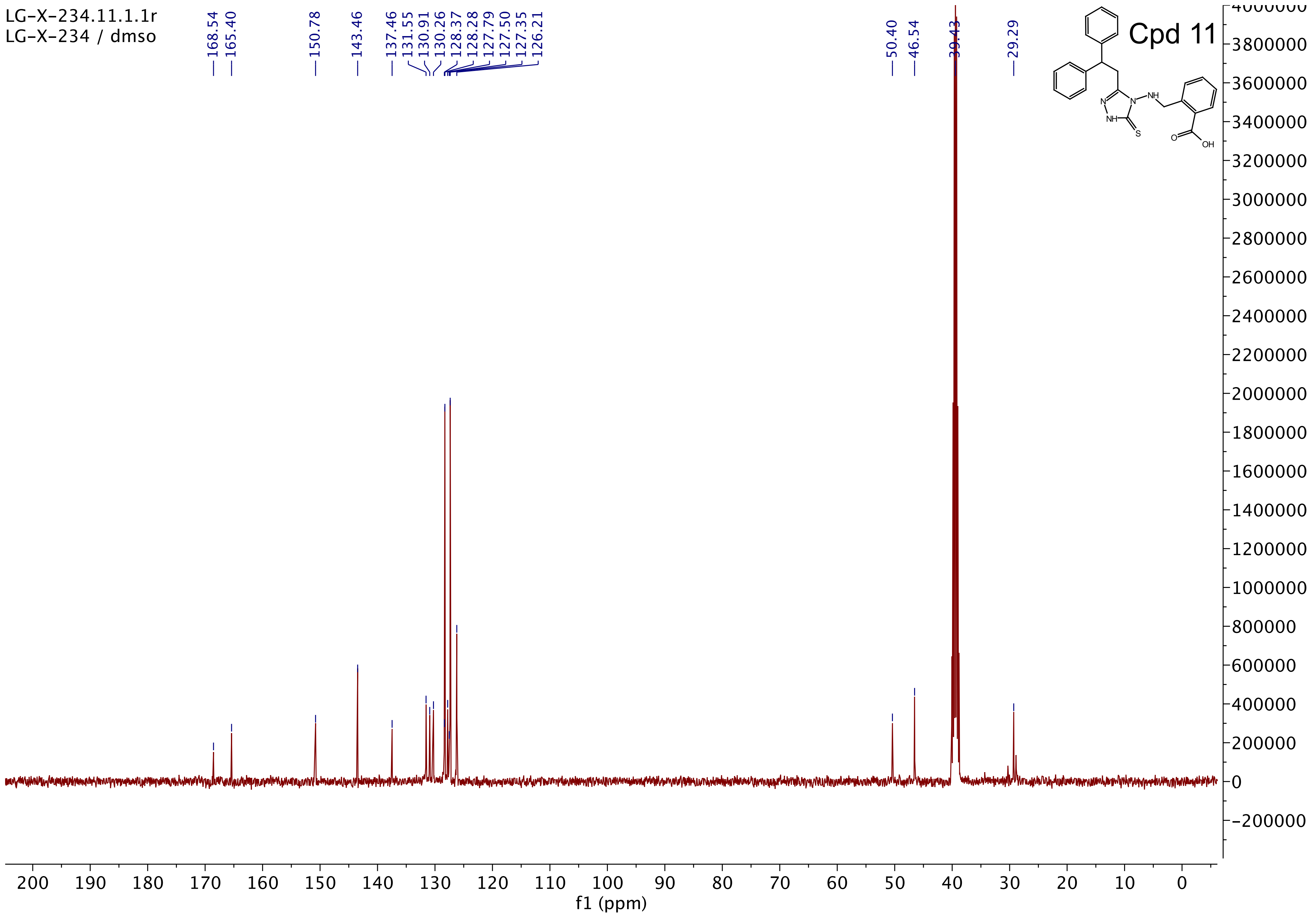

LG-X-234.11.1.1r
LG-X-234 / dmso
168.54
165.40
150.78
143.46
137.46
131.55
130.91
130.26
128.37
128.28
127.79
127.50
127.35
126.21
50.40
46.54
39.43
29.29
Cpd 11
f1 (ppm)

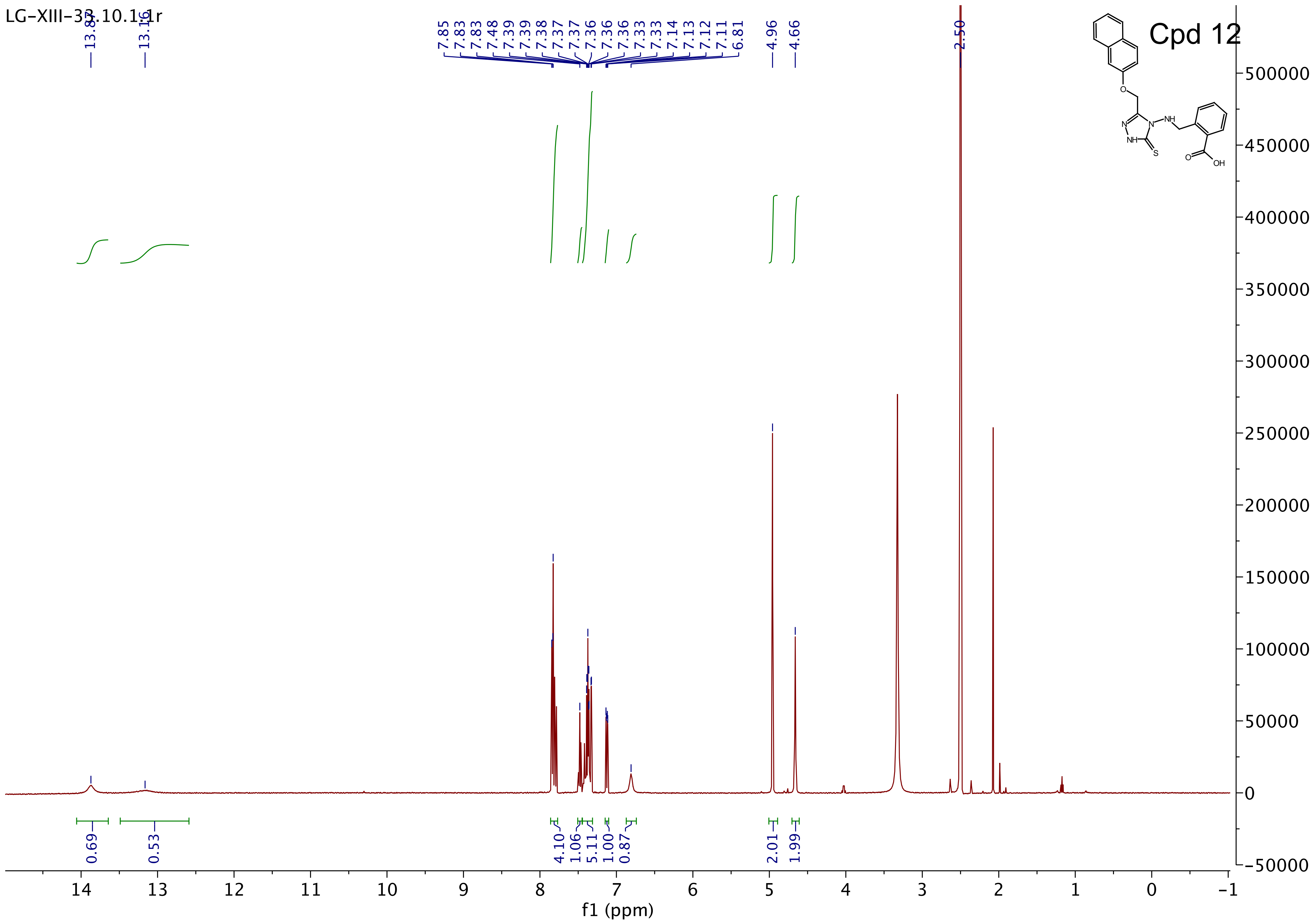

LG-XIII-33.10.1.1r
Cpd 12
13.87
13.16
7.85
7.83
7.83
7.48
7.39
7.39
7.38
7.37
7.37
7.36
7.36
7.36
7.33
7.33
7.14
7.13
7.12
7.11
6.81
4.96
4.66
2.50
0.69
0.53
4.10
1.06
5.11
1.00
0.87
2.01
1.99
f1 (ppm)

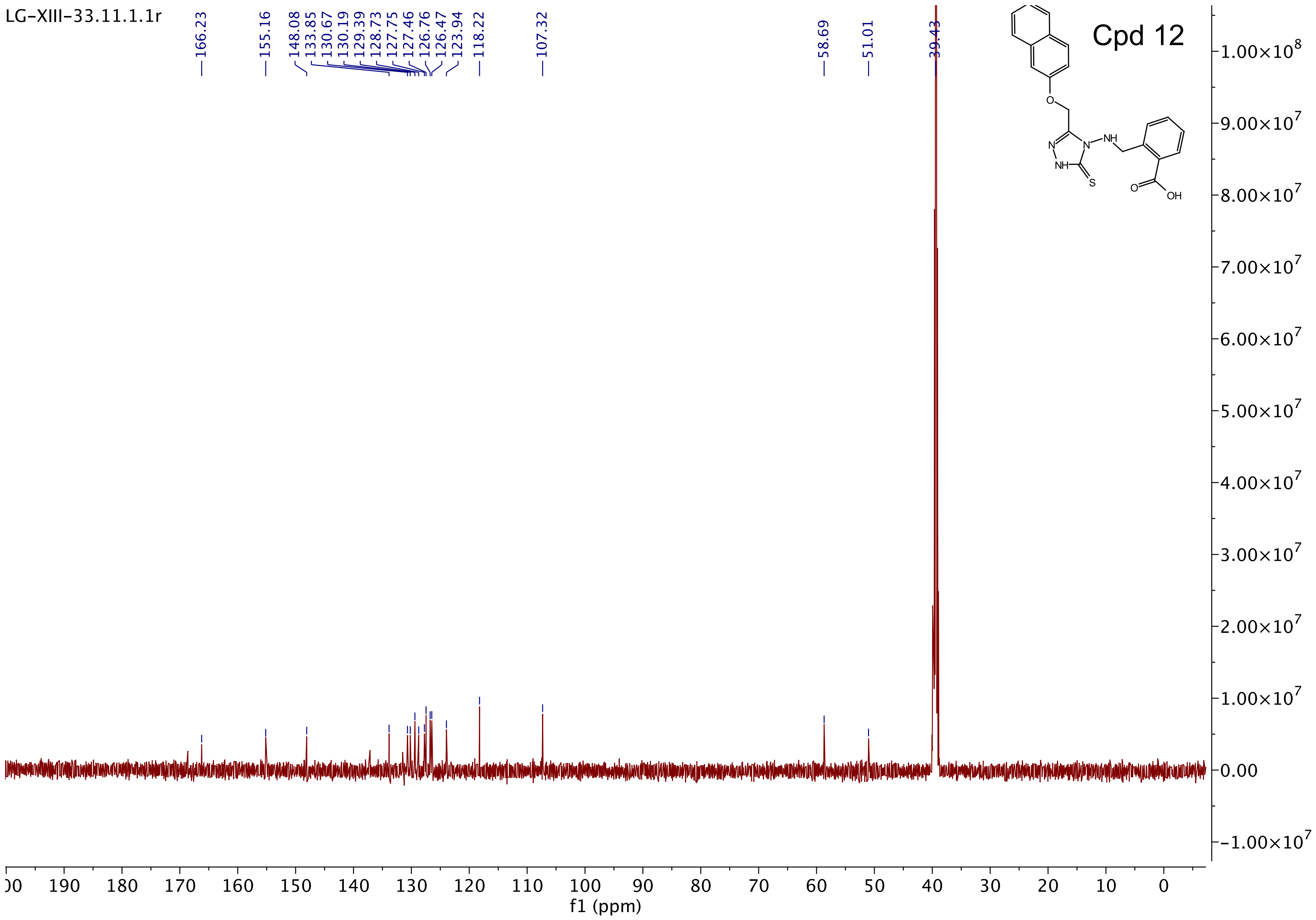
LG-XIII-33.11.1.1r
Cpd 12
166.23
155.16
148.08
133.85
130.67
130.19
129.39
128.73
127.75
127.46
126.76
126.47
123.94
118.22
107.32
58.69
51.01
39.43
f1 (ppm)

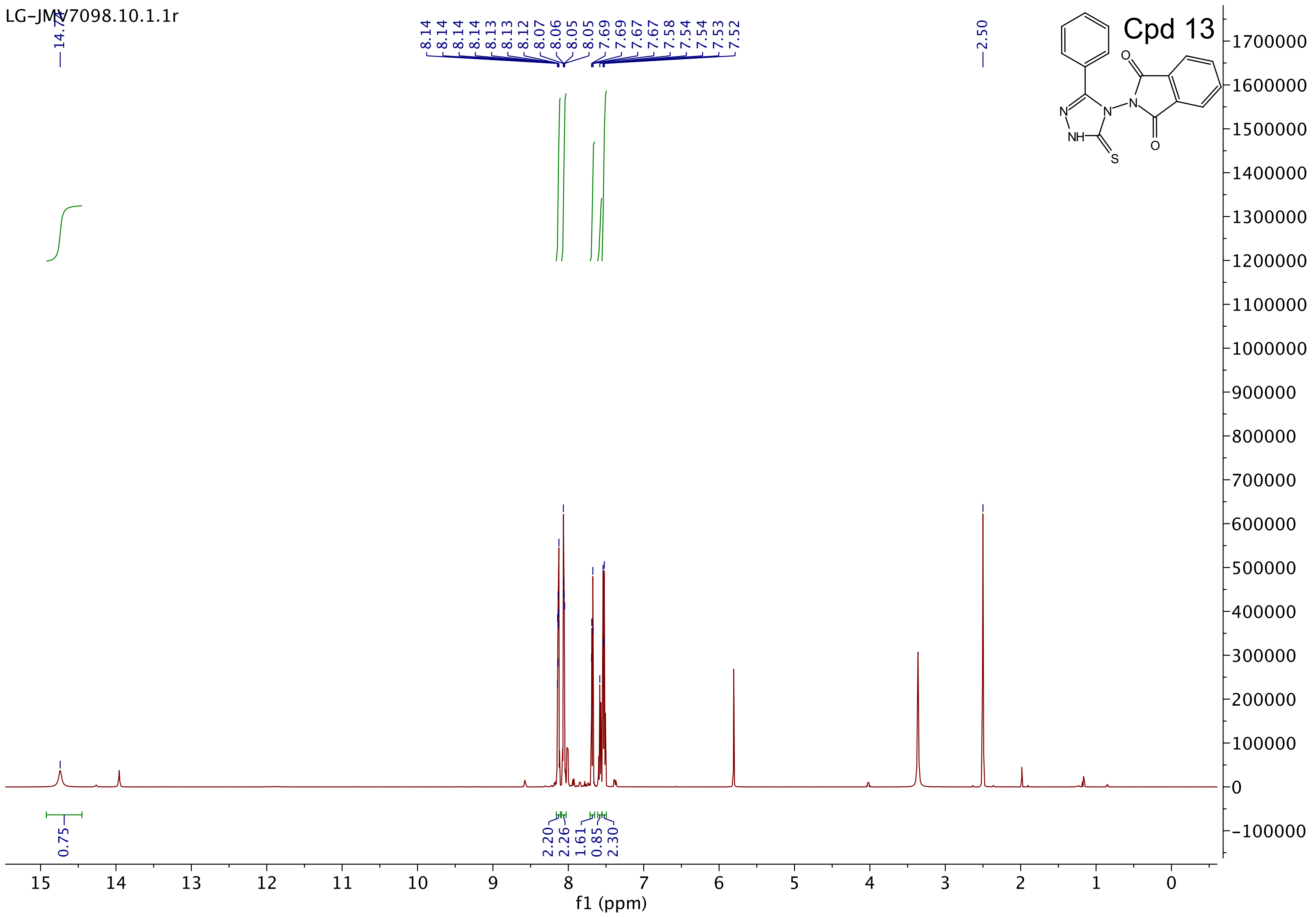

LG-JMV7098.10.1.1r
Cpd 13
14.74
8.14
8.14
8.14
8.14
8.13
8.13
8.12
8.07
8.06
8.05
8.05
7.69
7.69
7.67
7.67
7.58
7.54
7.54
7.53
7.52
2.50
0.75
2.20
2.26
1.61
0.85
2.30
f1 (ppm)

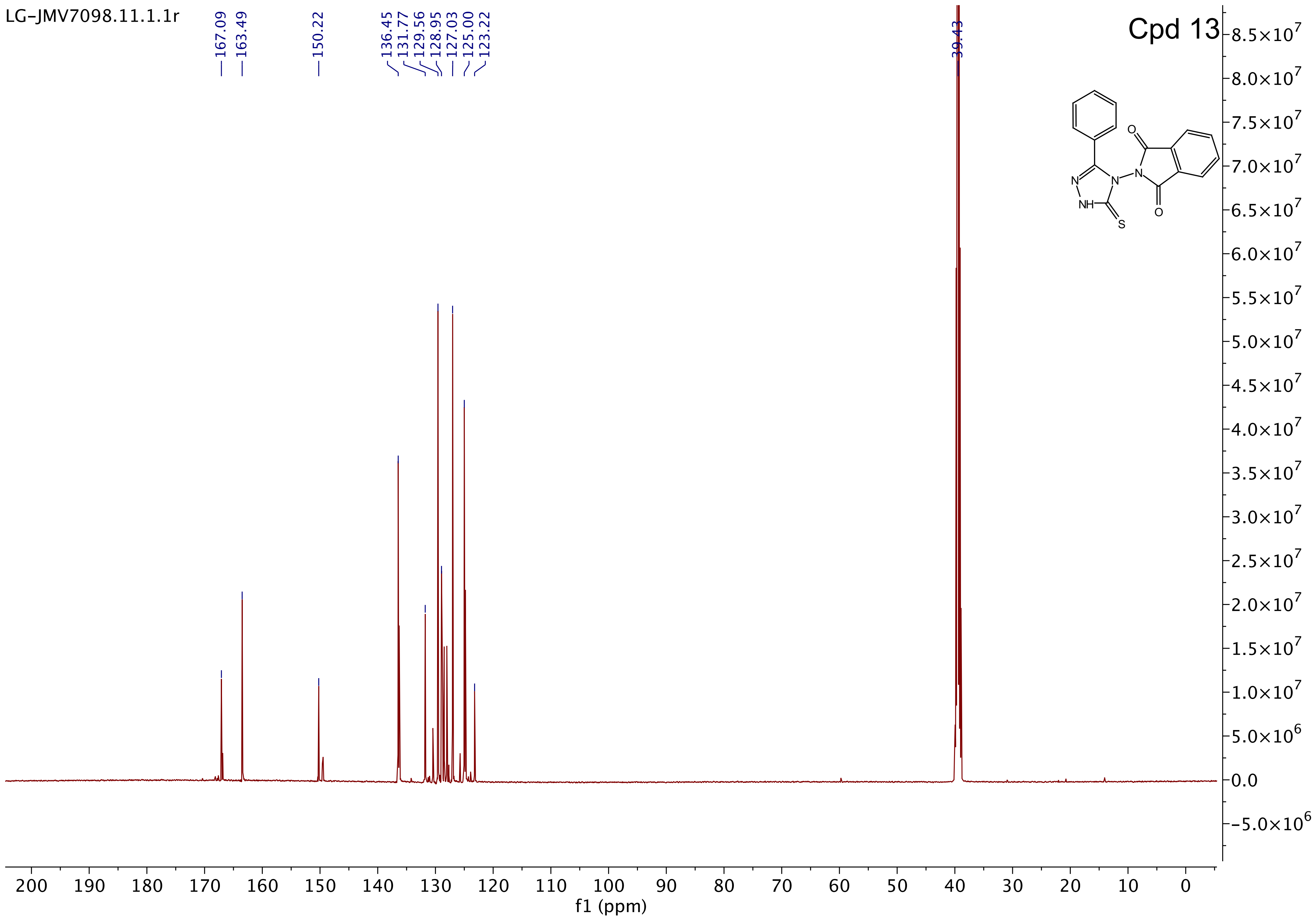
LG-JMV7098.11.1.1r
167.09
163.49
150.22
136.45
131.77
129.56
128.95
127.03
125.00
123.22
39.43
Cpd 13
N
NH
N
S
N
O
O
8.5×10^7
8.0×10^7
7.5×10^7
7.0×10^7
6.5×10^7
6.0×10^7
5.5×10^7
5.0×10^7
4.5×10^7
4.0×10^7
3.5×10^7
3.0×10^7
2.5×10^7
2.0×10^7
1.5×10^7
1.0×10^7
5.0×10^6
0.0
−5.0×10^6
200
190
180
170
160
150
140
130
120
110
100
90
80
70
60
50
40
30
20
10
0
f1 (ppm)

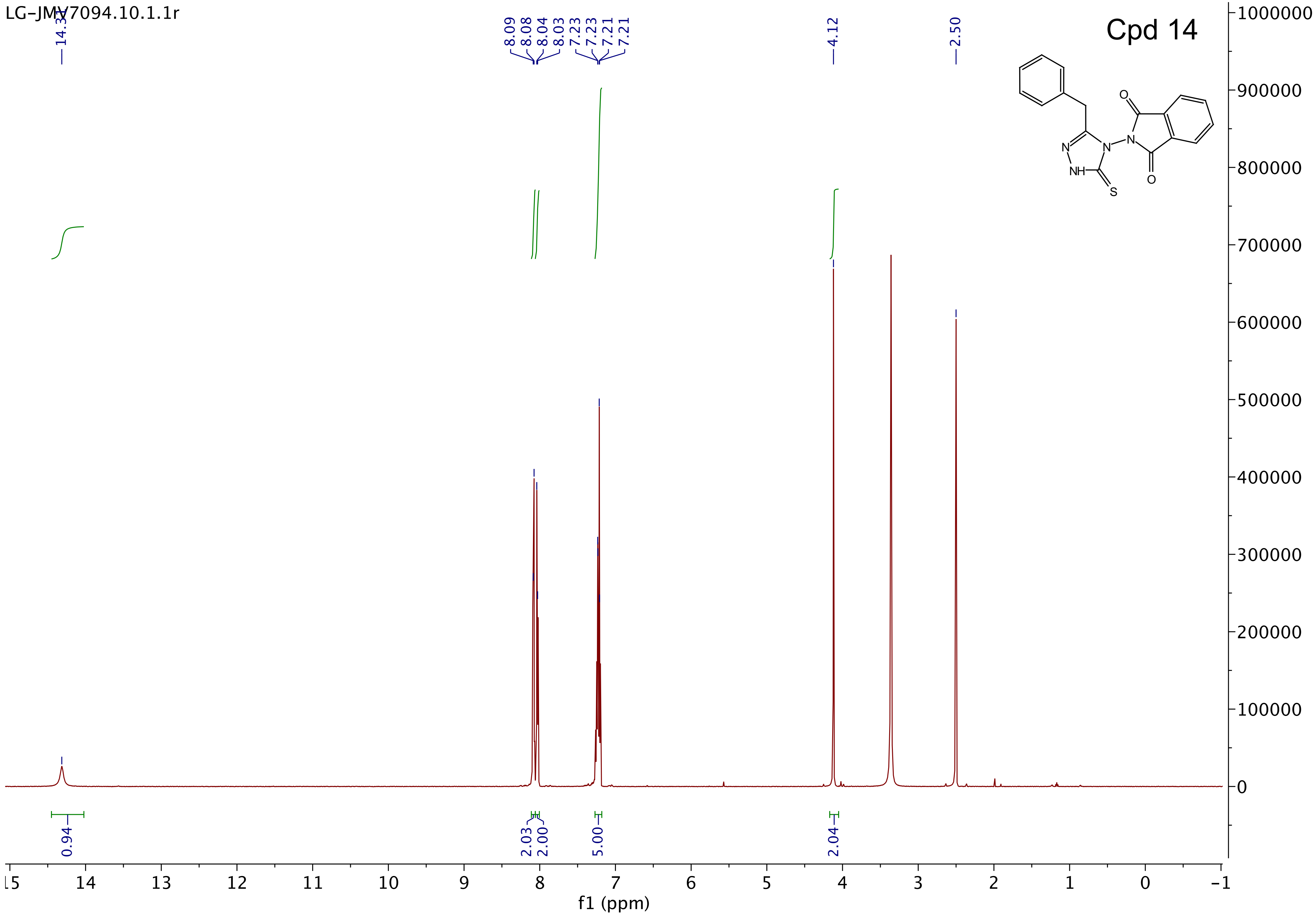

LG-JMV7094.10.1.1r
Cpd 14
14.31
8.09
8.08
8.04
8.03
7.23
7.23
7.21
7.21
4.12
2.50
O
N
N
NH
S
N
O
0.94
2.03
2.00
5.00
2.04
1000000
900000
800000
700000
600000
500000
400000
300000
200000
100000
0
15
14
13
12
11
10
9
8
7
6
5
4
3
2
1
0
−1
f1 (ppm)

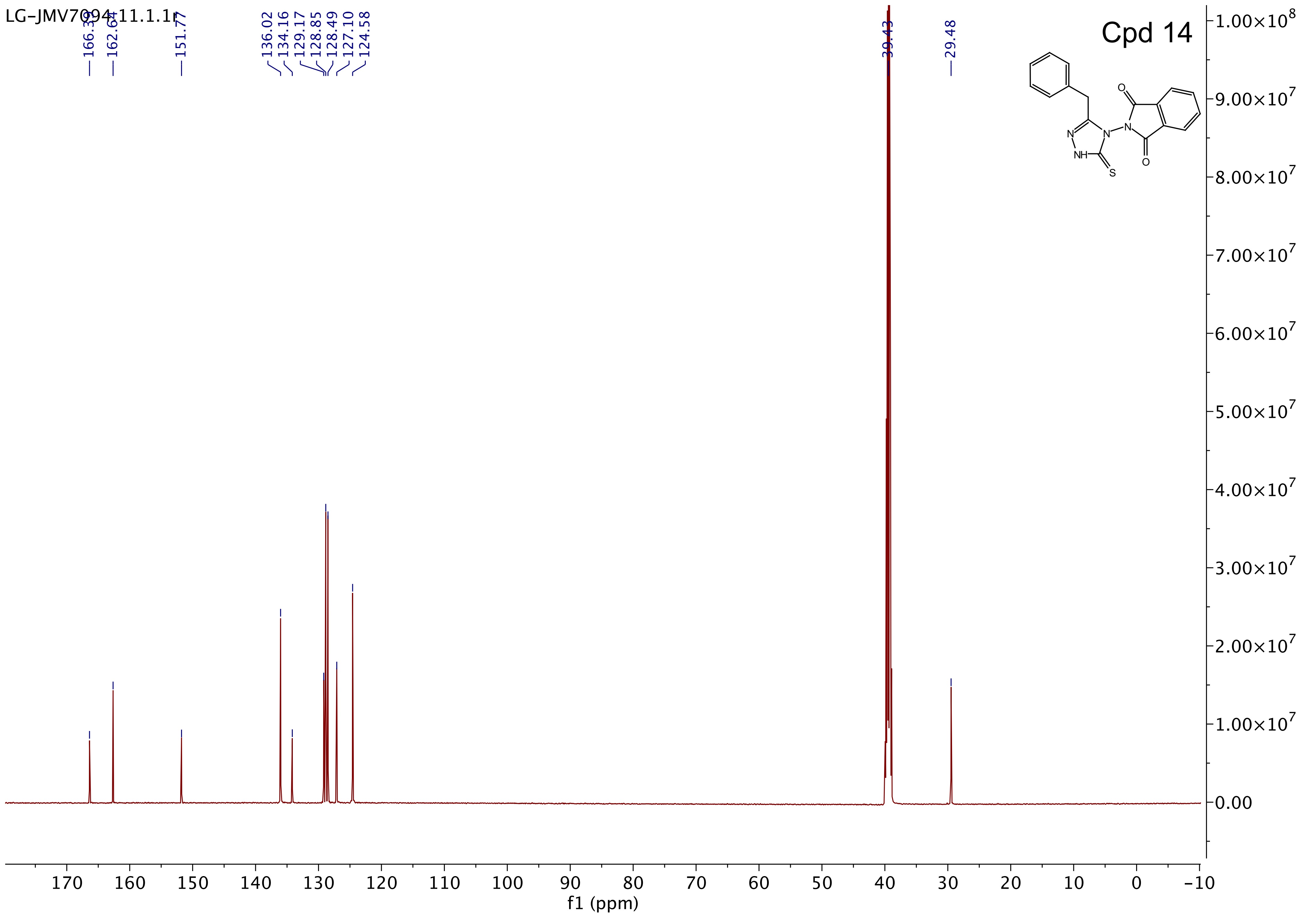

LG-JMV7094-11.1.1r
Cpd 14
151.77
136.02
134.16
129.17
128.85
128.49
127.10
124.58
29.48
O
N
N
NH
S
f1 (ppm)
170 160 150 140 130 120 110 100 90 80 70 60 50 40 30 20 10 0 −10

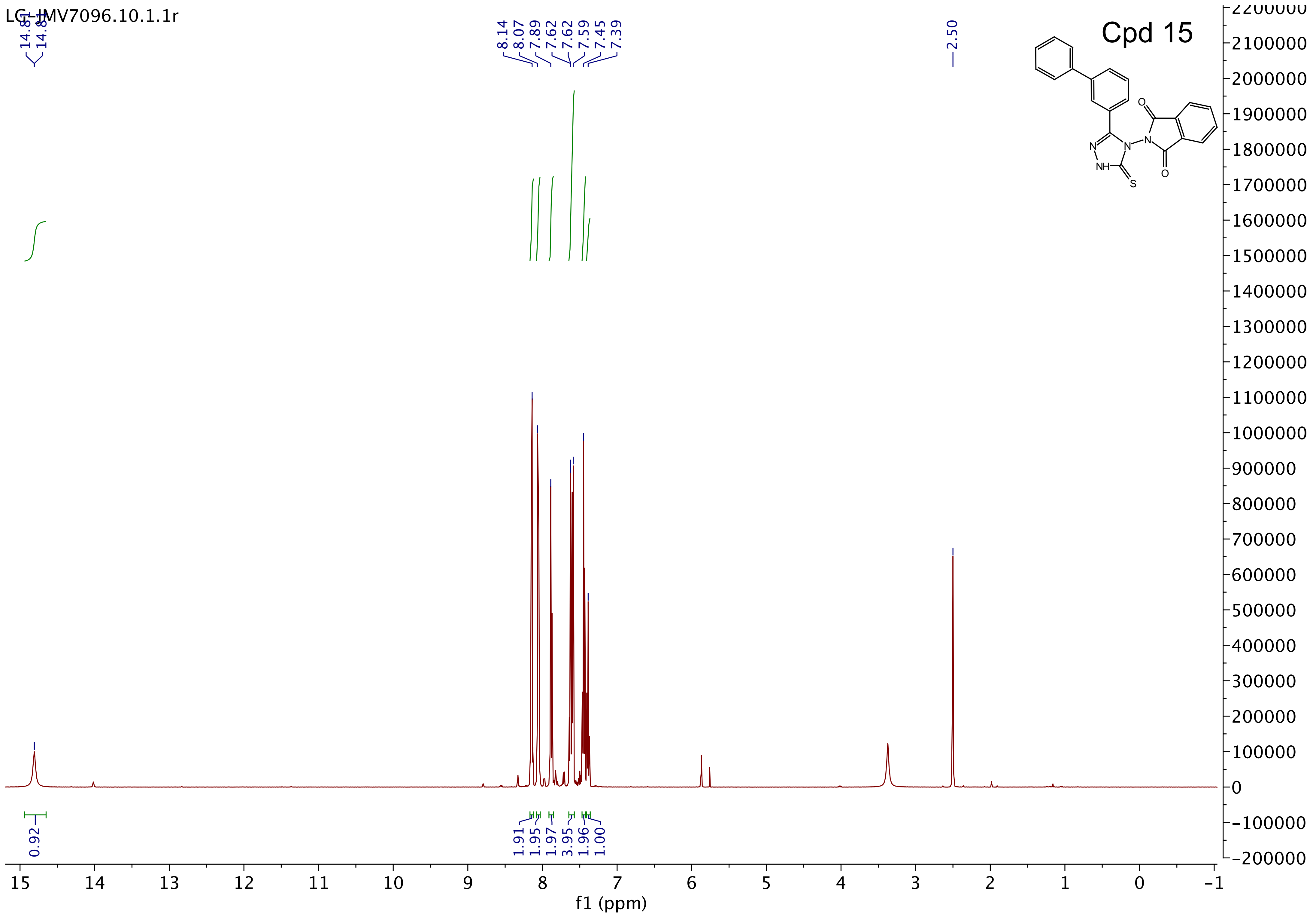
LG-JMV7096.10.1.1r
Cpd 15
14.81
14.81
8.14
8.07
7.89
7.62
7.62
7.59
7.45
7.39
2.50
0.92
1.91
1.95
1.97
3.95
1.96
1.00
f1 (ppm)

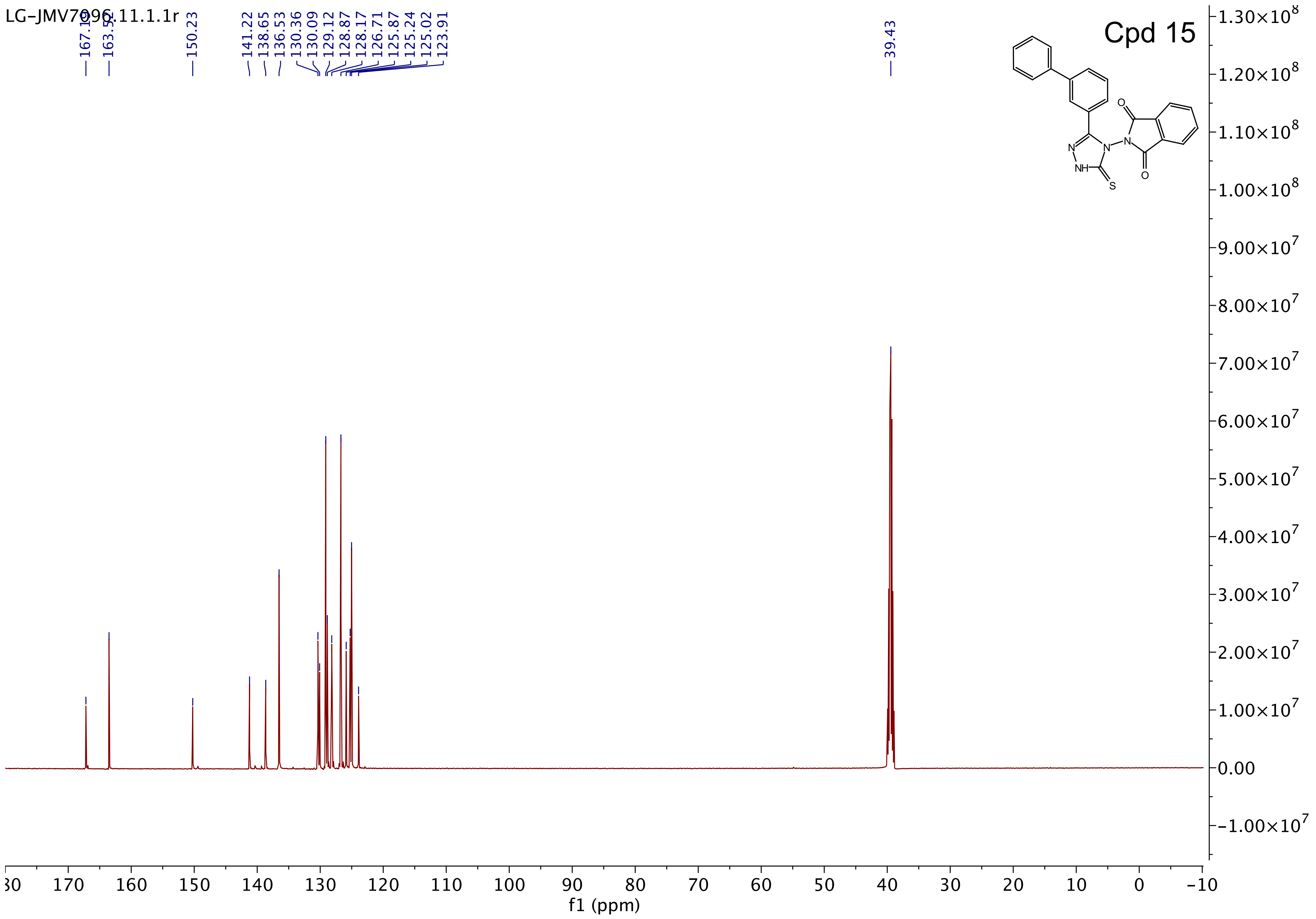

LG-JMV7096.11.1.1r
150.23
141.22
138.65
136.53
130.36
130.09
129.12
128.87
128.17
126.71
125.87
125.24
125.02
123.91
39.43
Cpd 15
O
N
N
NH
S
f1 (ppm)

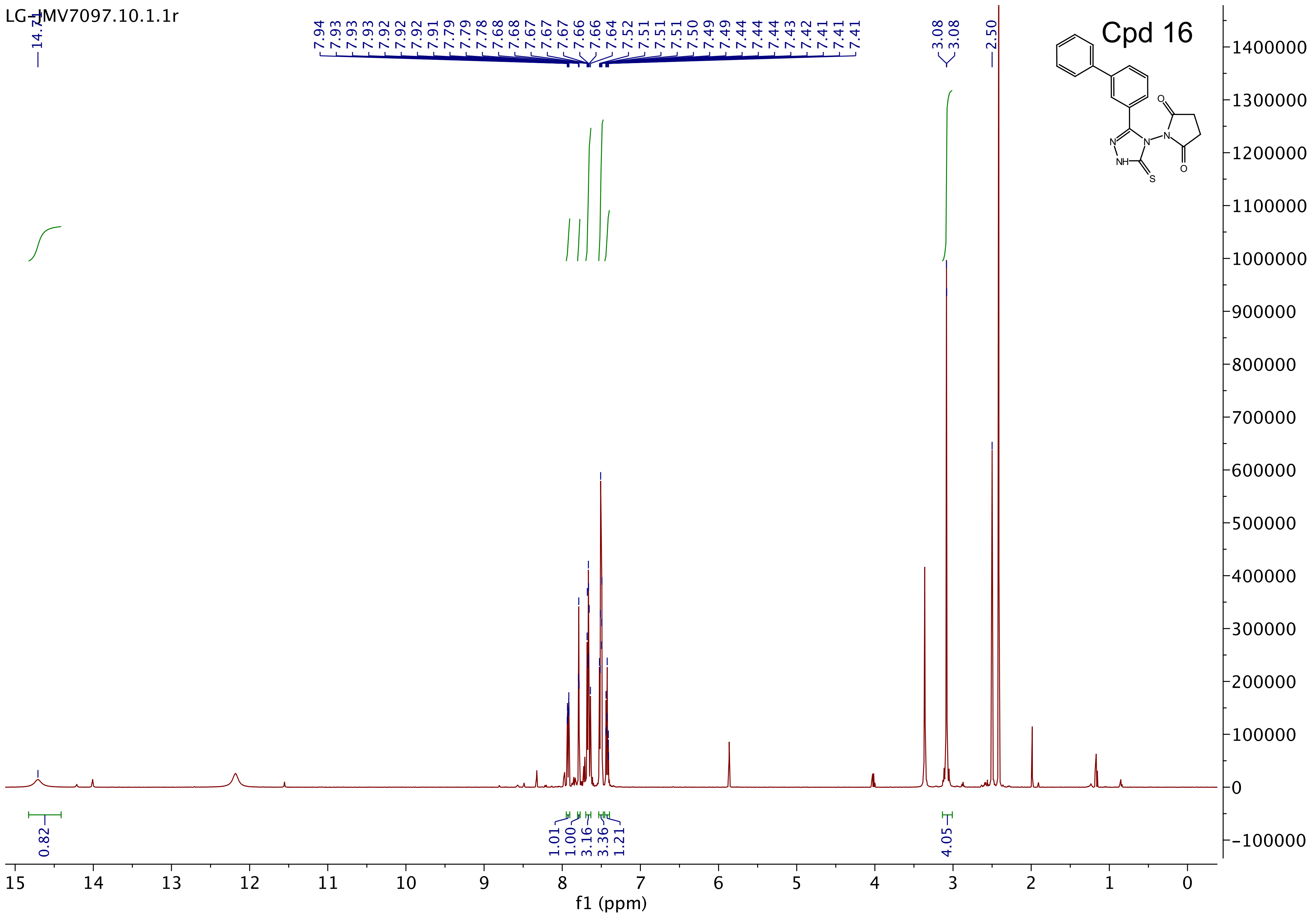

LG-IMV7097.10.1.1r
Cpd 16
14.71
7.94 7.93 7.93 7.93 7.92 7.92 7.92 7.91 7.79 7.79 7.78 7.68 7.68 7.67 7.67 7.67 7.66 7.66 7.64 7.52 7.51 7.51 7.51 7.50 7.49 7.49 7.44 7.44 7.44 7.43 7.42 7.41 7.41 7.41
3.08 3.08
2.50
0.82
1.01 1.00 3.16 3.36 1.21
4.05
f1 (ppm)

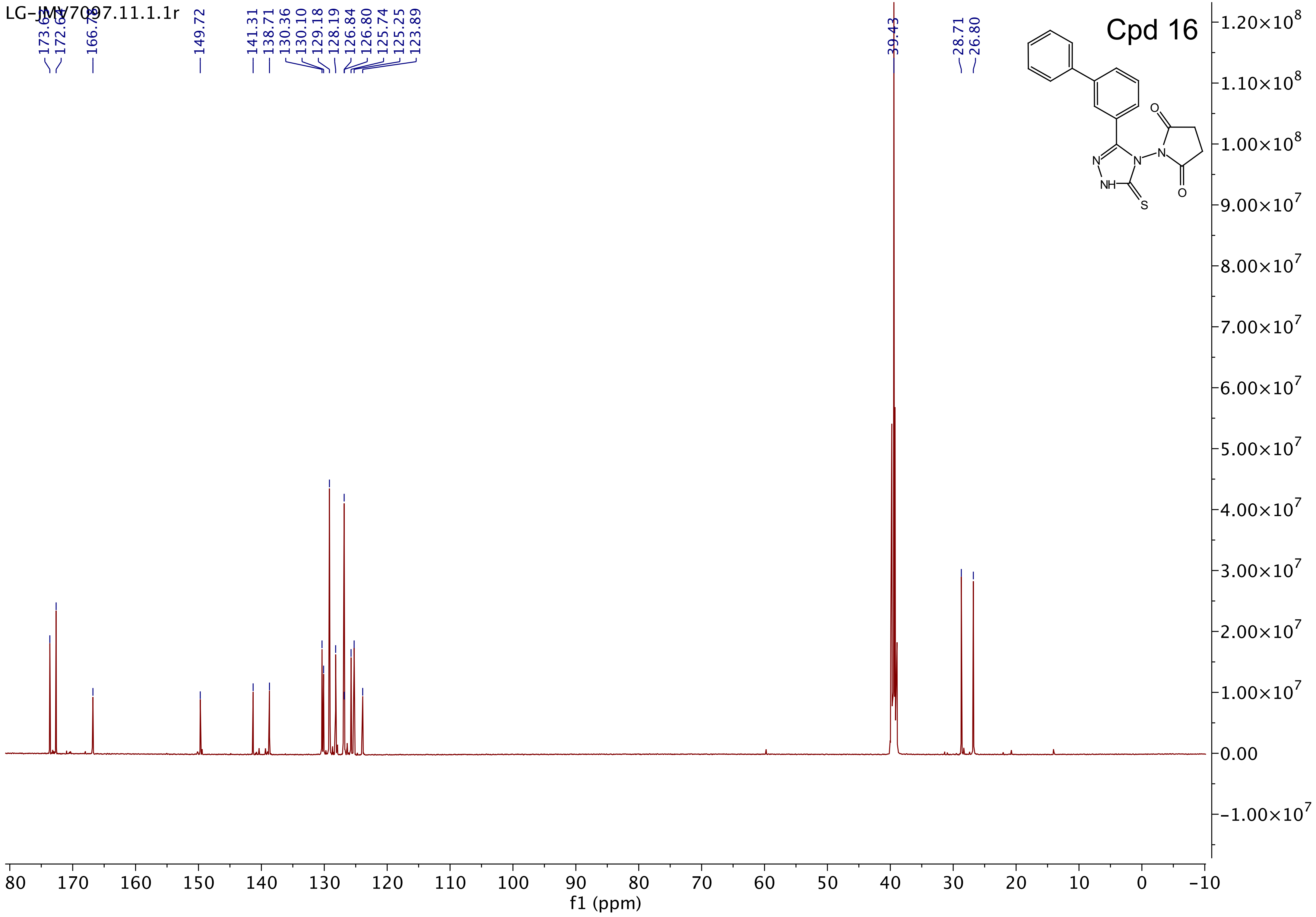

LG-JMV7097.11.1.1r
149.72
141.31
138.71
130.36
130.10
129.18
128.19
126.84
126.80
125.74
125.25
123.89
39.43
28.71
26.80
Cpd 16
O
N
N
NH
S
f1 (ppm)

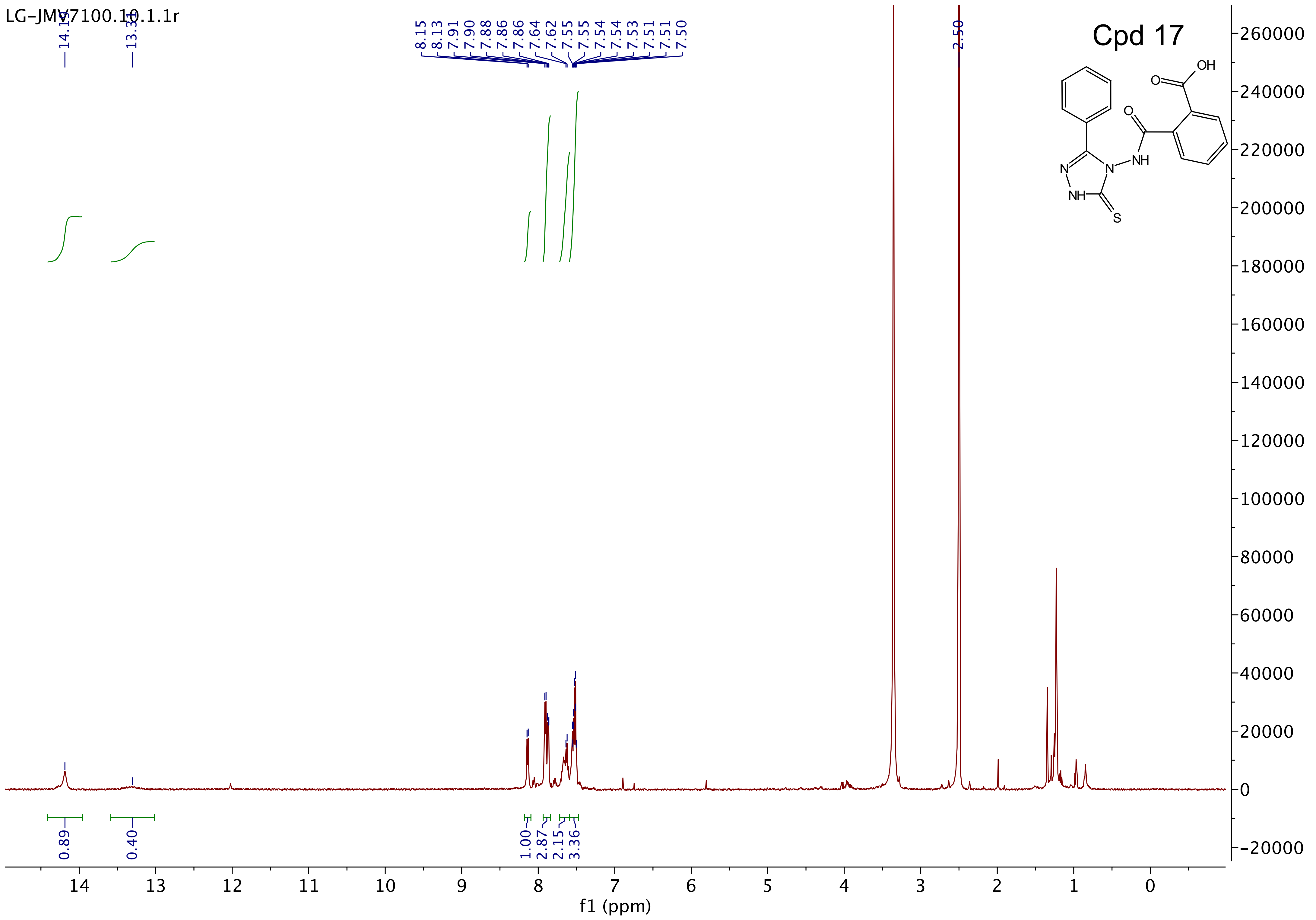

LG-JMV7100.10.1.1r
Cpd 17
8.15
8.13
7.91
7.90
7.88
7.86
7.86
7.64
7.62
7.55
7.55
7.54
7.54
7.53
7.51
7.51
7.50
2.50
OH
O
O
NH
N
N
NH
S
0.89
0.40
1.00
2.87
2.15
3.36
f1 (ppm)

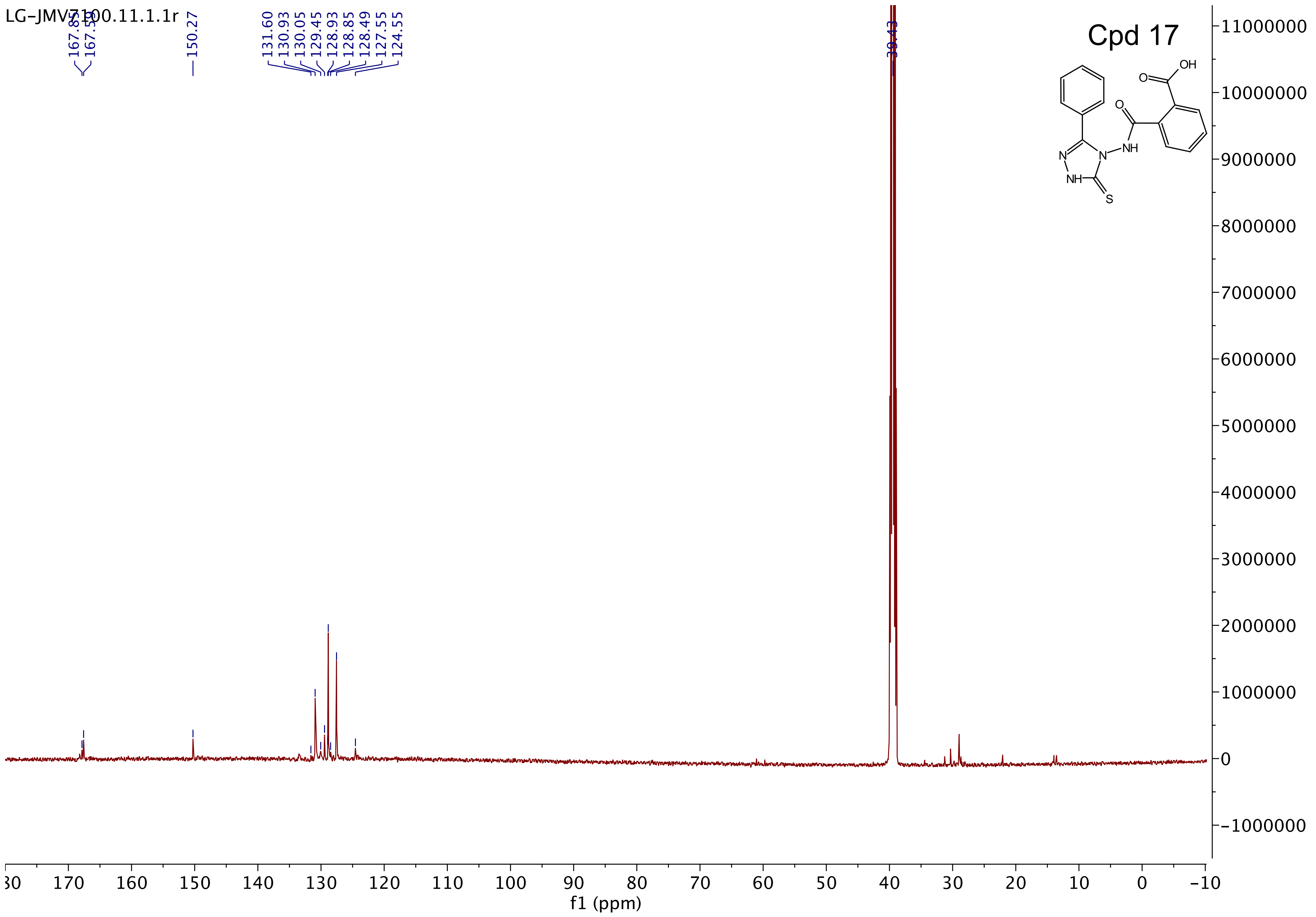
LG-JMV7100.11.1.1r
167.85
167.50
150.27
131.60
130.93
130.05
129.45
128.93
128.85
128.49
127.55
124.55
39.43
Cpd 17
OH
O
O
N
NH
N
NH
S
11000000
10000000
9000000
8000000
7000000
6000000
5000000
4000000
3000000
2000000
1000000
0
-1000000
30
170
160
150
140
130
120
110
100
90
80
70
60
50
40
30
20
10
0
-10
f1 (ppm)

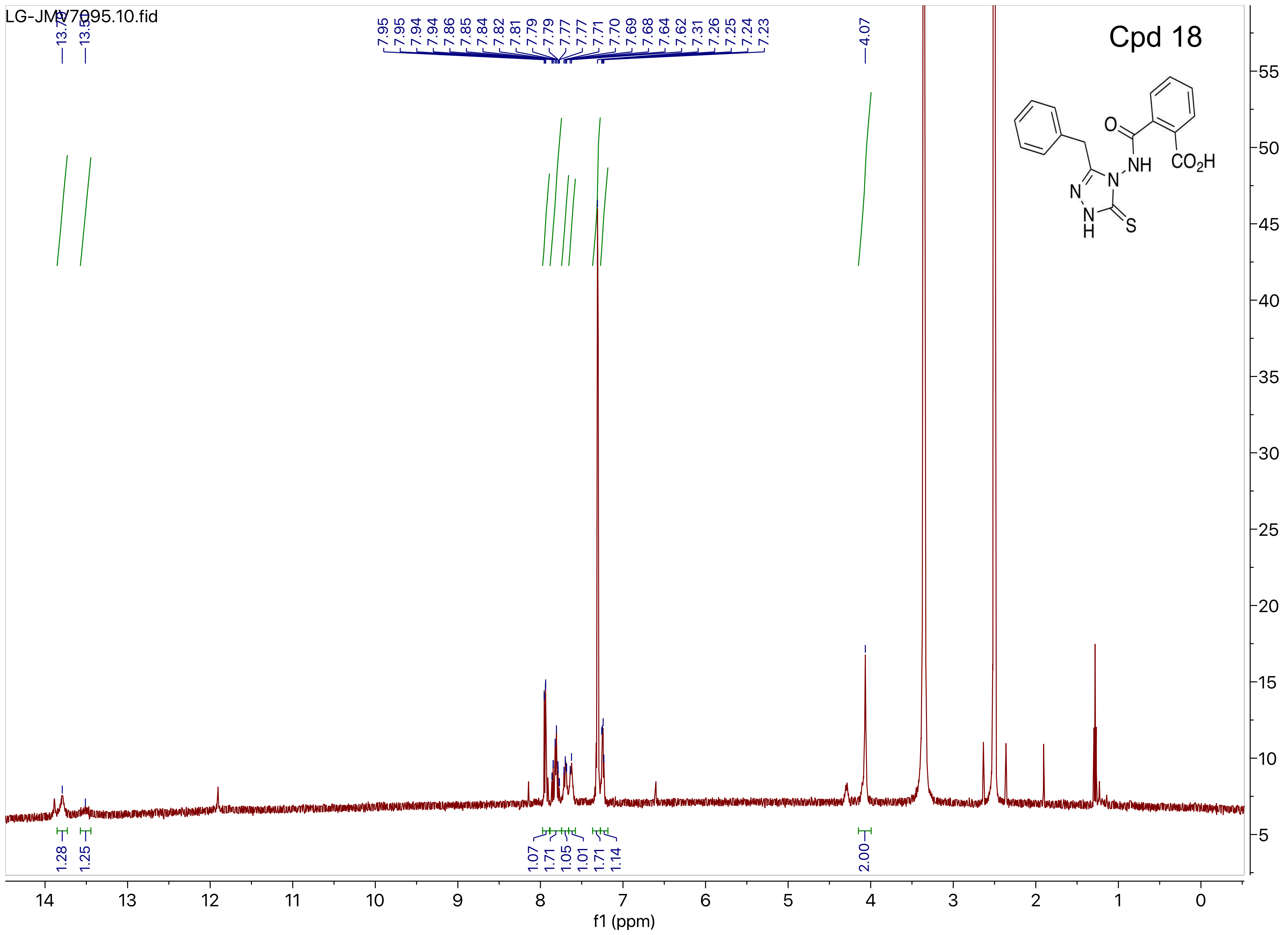

LG-JMV7095.10.fid
Cpd 18
7.95 7.95 7.94 7.94 7.86 7.85 7.84 7.82 7.81 7.79 7.79 7.77 7.77 7.71 7.70 7.69 7.68 7.64 7.62 7.31 7.26 7.25 7.24 7.23
4.07
1.28 1.25
1.07 1.71 1.05 1.01 1.71 1.14
2.00
O
NH
CO_2H
N
N
N
H
S
55 50 45 40 35 30 25 20 15 10 5
14 13 12 11 10 9 8 7 6 5 4 3 2 1 0
f1 (ppm)

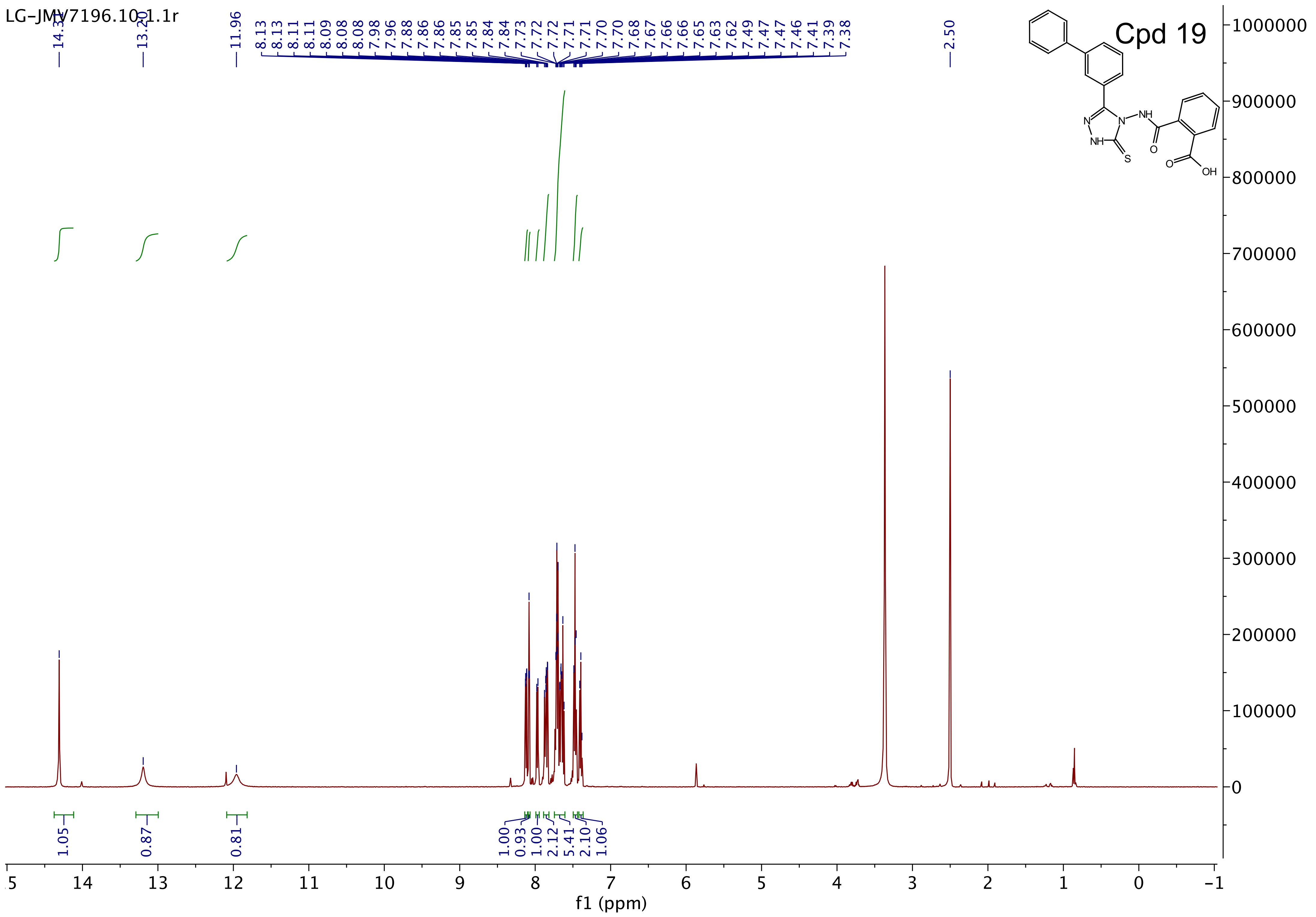

LG-JMV7196.10.1.1r
Cpd 19
14.31
13.20
11.96
8.13
8.13
8.11
8.11
8.09
8.08
8.08
7.98
7.96
7.88
7.86
7.86
7.85
7.85
7.84
7.84
7.73
7.72
7.72
7.71
7.71
7.70
7.70
7.68
7.67
7.66
7.66
7.65
7.63
7.62
7.49
7.47
7.47
7.46
7.41
7.39
7.38
2.50
1.05
0.87
0.81
1.00
0.93
1.00
2.12
5.41
2.10
1.06
f1 (ppm)

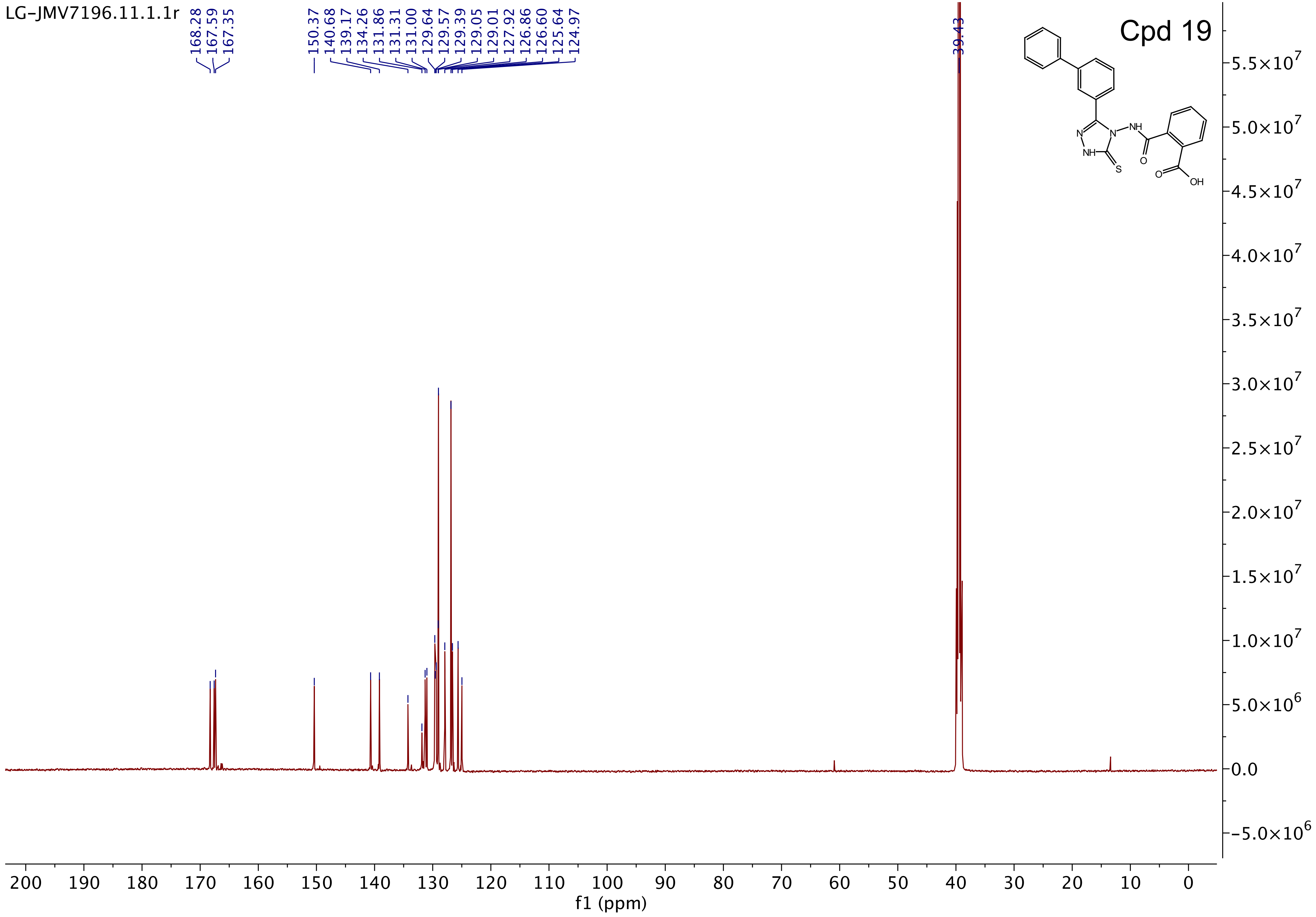
LG-JMV7196.11.1.1r
168.28
167.59
167.35
150.37
140.68
139.17
134.26
131.86
131.31
131.00
129.64
129.57
129.39
129.05
129.01
127.92
126.86
126.60
125.64
124.97
39.43
Cpd 19
NH
N
NH
N
S
O
O
OH
5.5×10^7
5.0×10^7
4.5×10^7
4.0×10^7
3.5×10^7
3.0×10^7
2.5×10^7
2.0×10^7
1.5×10^7
1.0×10^7
5.0×10^6
0.0
−5.0×10^6
200 190 180 170 160 150 140 130 120 110 100 90 80 70 60 50 40 30 20 10 0
f1 (ppm)

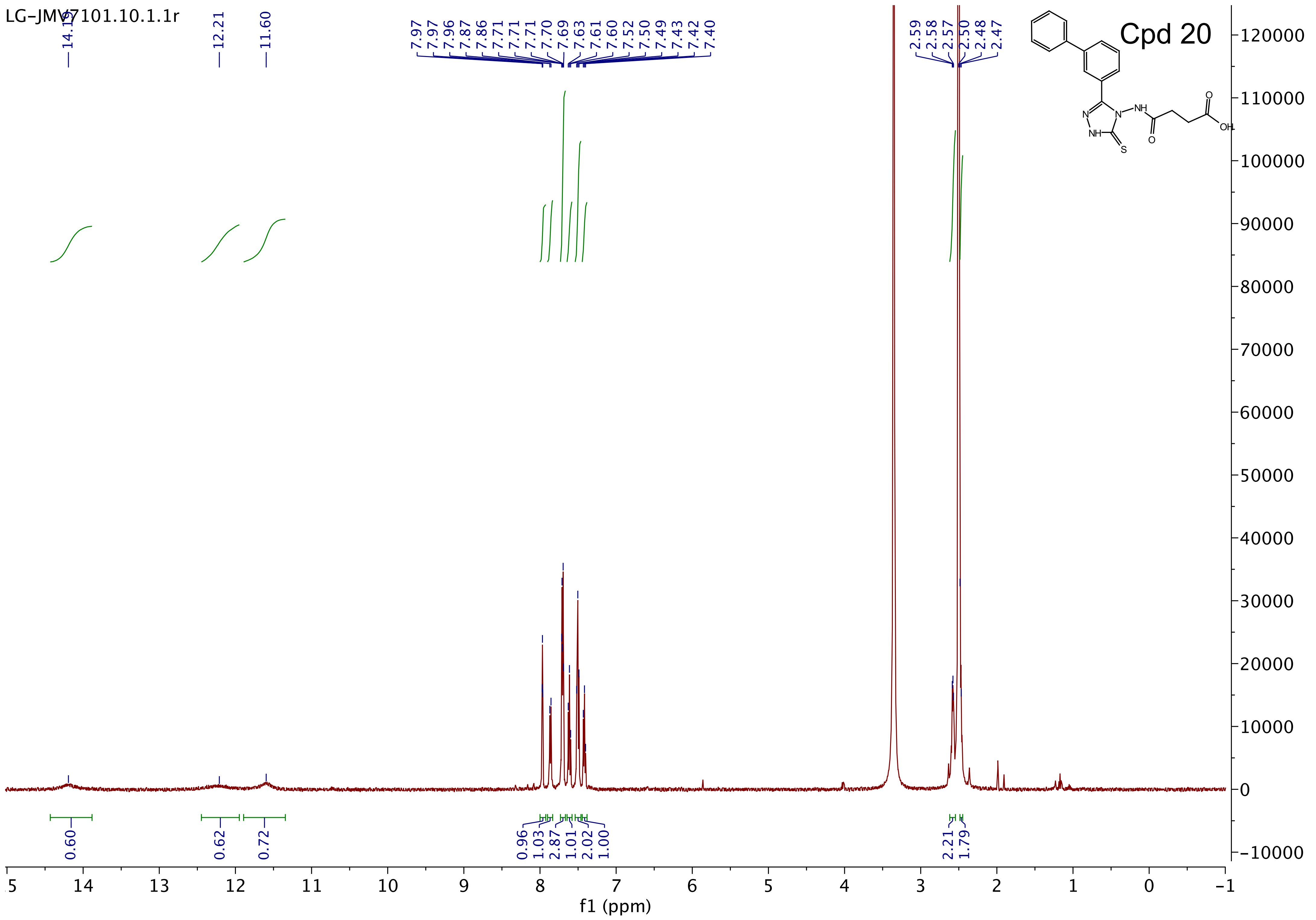

LG–JMV7101.10.1.1r
Cpd 20
14.19
12.21
11.60
7.97
7.97
7.96
7.87
7.86
7.71
7.71
7.71
7.70
7.69
7.63
7.61
7.60
7.52
7.50
7.49
7.43
7.42
7.40
2.59
2.58
2.57
2.50
2.48
2.47
0.60
0.62
0.72
0.96
1.03
2.87
1.01
2.02
1.00
2.21
1.79
N
NH
N
NH
S
O
O
OH
f1 (ppm)

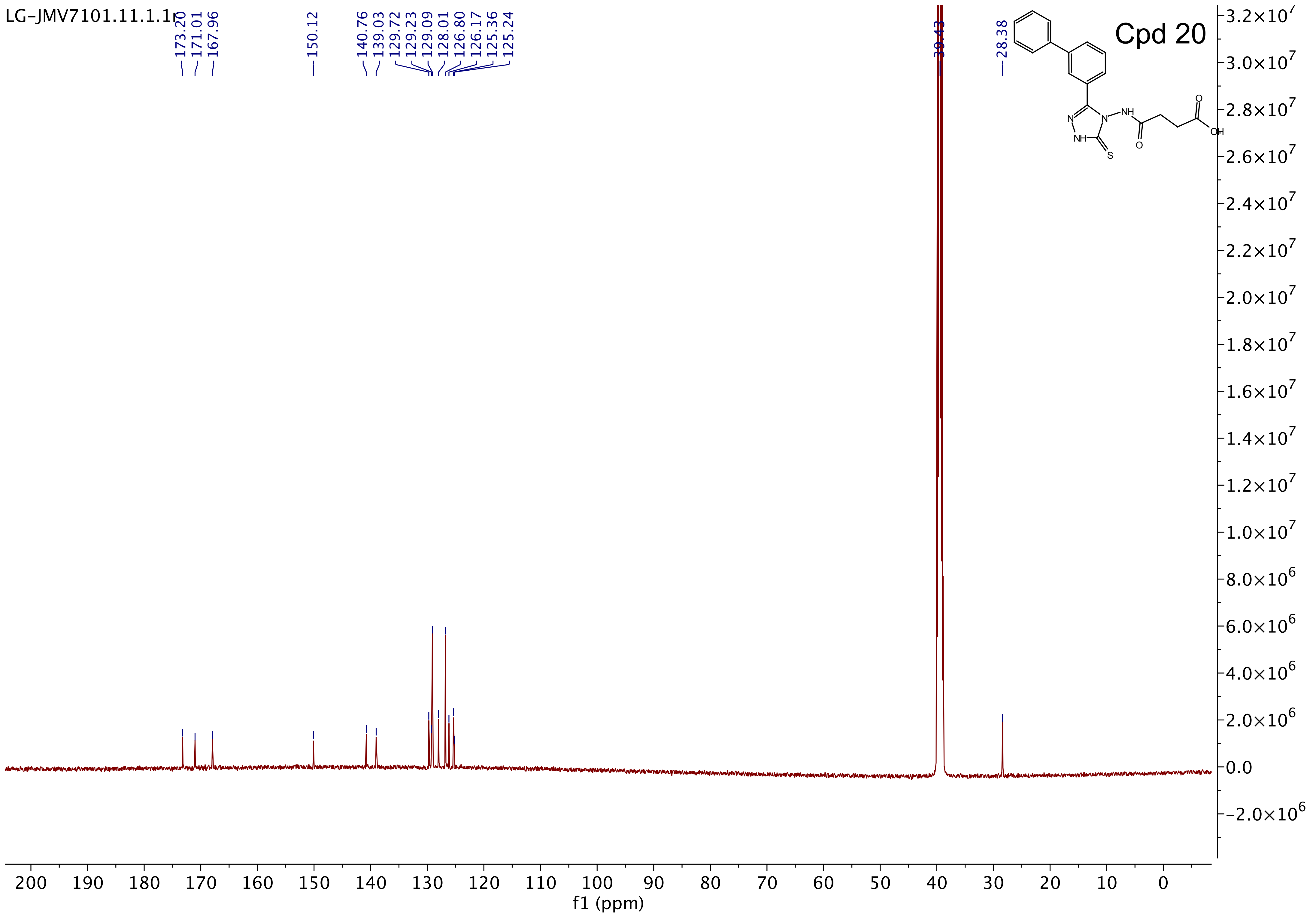

LG-JMV7101.11.1.1r
173.20
171.01
167.96
150.12
140.76
139.03
129.72
129.23
129.09
128.01
126.80
126.17
125.36
125.24
39.43
28.38
Cpd 20
NH
N
N
NH
S
O
O
OH
200 190 180 170 160 150 140 130 120 110 100 90 80 70 60 50 40 30 20 10 0
f1 (ppm)
3.2×10^7 3.0×10^7 2.8×10^7 2.6×10^7 2.4×10^7 2.2×10^7 2.0×10^7 1.8×10^7 1.6×10^7 1.4×10^7 1.2×10^7 1.0×10^7 8.0×10^6 6.0×10^6 4.0×10^6 2.0×10^6 0.0 −2.0×10^6
